# Monocarbonyl Analogs of Curcumin Based on the Pseudopelletierine Scaffold: Synthesis and Anti-Inflammatory Activity

**DOI:** 10.3390/ijms222111384

**Published:** 2021-10-21

**Authors:** Damian Pawelski, Alicja Walewska, Sylwia Ksiezak, Dariusz Sredzinski, Piotr Radziwon, Marcin Moniuszko, Ramesh Gandusekar, Andrzej Eljaszewicz, Ryszard Lazny, Krzysztof Brzezinski, Marta E. Plonska-Brzezinska

**Affiliations:** 1 Department of Organic Chemistry, Faculty of Pharmacy with the Division of Laboratory Medicine, Medical University of Bialystok, Mickiewicza 2A, 15-222 Bialystok, Poland; damian.pawelski@umb.edu.pl; 2 Department of Regenerative Medicine and Immune Regulation, Medical University of Bialystok, Waszyngtona 13, 15-269 Bialystok, Poland; alicja.walewska@umb.edu.pl (A.W.); sylwia.ksiezak@icloud.com (S.K.); marcin.moniuszko@umb.edu.pl (M.M.); ramesh.gandusekar@umb.edu.pl (R.G.); 3 Regional Blood Donation and Blood Treatment Center in Bialystok, M. Sklodowskiej-Curie 23, 15-950 Bialystok, Poland; dsredzinski@rckik.bialystok.pl (D.S.); piotr.radziwon@umb.edu.pl (P.R.); 4 Department of Hematology, Medical University of Bialystok, M. Sklodowskiej-Curie 24A, 15-276 Bialystok, Poland; 5 Department of Allergology and Internal Medicine, Medical University of Bialystok, M. Sklodowskiej-Curie 24A, 15-276 Bialystok, Poland; 6 Faculty of Chemistry, University of Bialystok, Ciolkowskiego 1K, 15-245 Bialystok, Poland; lazny@uwb.edu.pl; 7 Department of Structural Biology of Prokaryotic Organisms, Institute of Bioorganic Chemistry, Polish Academy of Sciences, Noskowskiego 12/14, 61-074 Poznan, Poland

**Keywords:** curcumin, pseudopelletierine, granatanone, granatane, anti-inflammatory property, cytotoxicity, cytokines

## Abstract

Curcumin (CUR) is a natural compound that exhibits anti-inflammatory, anti-bacterial, and other biological properties. However, its application as an effective drug is problematic due to its poor oral bioavailability, solubility in water, and poor absorption from the gastrointestinal tract. The aim of this work is to synthesize monocarbonyl analogs of CUR based on the 9-methyl-9-azabicyclo[3.2.1]nonan-3-one (pseudopelletierine, granatanone) scaffold to improve its bioavailability. Granatane is a homologue of tropane, whose structure is present in numerous naturally occurring alkaloids, e.g., l-cocaine and l-scopolamine. In this study, ten new pseudopelletierine-derived monocarbonyl analogs of CUR were successfully synthesized and characterized by spectral methods and X-ray crystallography. Additionally, in vitro test of the cytotoxicity and anti-inflammatory properties of the synthesized compounds were performed.

## 1. Introduction

**Scheme 1:**
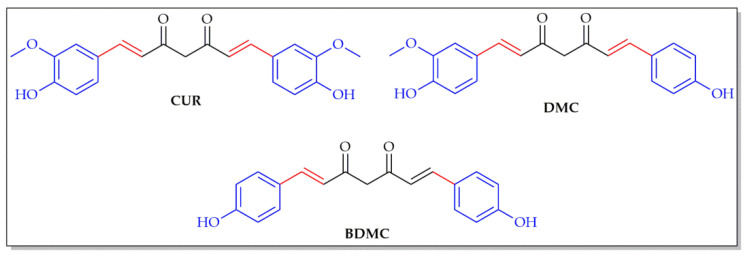
Structures of natural curcuminoids found in the *Curcuma longa* rhizome.

Curcuminoids are biologically active drug candidates that include such compounds as curcumin (CUR), demethoxycurcumin (DMC), and bisdemethoxycurcumin (BDMC) (Scheme 1). They occur naturally in the rhizome of *Curcuma longa* [1]. Among the natural curcuminoids, the greatest medical importance is found on CUR [2,3,4,5]. Comprehensive preclinical studies on tissue cultures and animal models indicate that natural curcuminoids reveal a number of biological activities, including antioxidant [6,7,8,9], antimicrobal [10,11,12,13], anti-inflammatory [14,15,16], anticancer [17], and neuroprotective [18,19] properties.

**Scheme 2:**
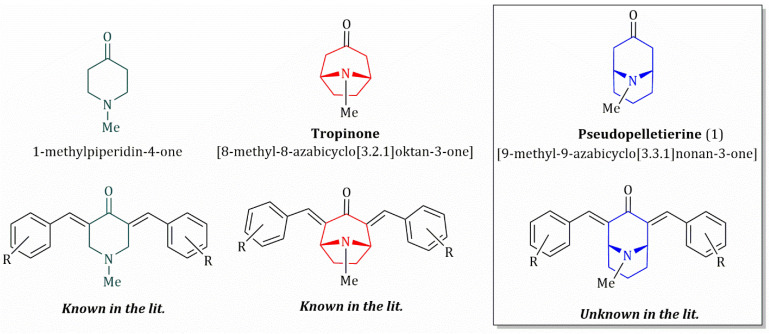
Monocarbonyl analogs of CUR containing the heterocyclic scaffold.

Inflammation is a complex response of the body’s tissues to different stressors, such as pathogen damage, cell-derived factors, allergens, and pollutants [20,21]. The anti-inflammatory compounds can decrease the inflammatory cascade by a variety of different mechanisms [22]. The anti-inflammatory effect of curcuminoids may be mediated by the upregulation of peroxisome proliferator-activated receptor-*γ* activation [23,24], by inhibiting arachidonic acid metabolism [25], and by reducing inflammatory mucosal cytokine production [26]. In addition, CUR acts as a non-stressful and non-cytotoxic compound protecting epithelial cells against oxidative stress by inducing heme oxygenase-1 expression and reducing reactive oxygen [27]. In the literature, monocarbonyl analogs of CUR based on non-heterocyclic and heterocyclic (piperidin-4-one and tropinone) scaffolds are also known [28,29,30] (Scheme 2). In vitro tests have demonstrated the several anti-cancer activities of monocarbonyl analogs of CUR [31,32,33,34]. However, reports on monocarbonyl analogs of CUR, based on the granatanone scaffold, are elusive [35].

It is a well known phenomenon that the increase in drug solubility is correlated with its better absorption from gastrointestinal tract [36]. Accordingly, CUR analogs based on the granatanone scaffold should be more soluble in water due to the presence of a basic heterocyclic nitrogen atom, especially in the form of a salt. Granatanone is a tropinone homologue (Scheme 2). A number of naturally occurring alkaloids, such as l-cocaine, l-scopolamine, or l-hyoscyamine, contain the tropane scaffold [37], which has the ability to cross the blood–brain barrier and interact with nervous system receptors (muscarinic, nicotinic, and cholinergic) [38]. Studies with mice medicated with radiolabeled CUR have shown that the highest concentration of the compound after ingestion occurs in the small intestine, liver, kidneys, and lungs [39]. Relatively low levels of CUR were found in the brain tissue. A number of studies on the pharmacodynamics and pharmacokinetics of CUR have shown that the main disadvantage of this compound as a potential drug is its poor water solubility [40,41]. Thus, one of the ways to increase the attractiveness of a potential CUR-derived drug is increasing water solubility and absorption from the gastrointestinal tract [42,43,44].

Several methods have been developed to prepare various dosage forms of CUR—for example, nanoparticles [43,45], nanodisks [42], liposomes [46], micelles [47], complexes with other compounds [48,49,50], or incorporation within the polymeric matrix [51]—to increase CUR solubility and specific CUR delivery. Another way to increase the solubility of CUR is to synthesize its derivatives, such as CUR glucuronide [52]. Unfortunately, the derivatives of CUR frequently exhibit a lower biological activity and capacity than the non-modified form of CUR [52]. Natural curcuminoids are not stable, especially in solutions. CUR easily decomposes in the alkaline environment, e.g., by solvolysis, while it retains moderate stability in acidic solutions [5]. In neutral and slightly acidic environment, CUR may undergo cyclization reactions, e.g., into a bicyclopentanedione derivative [27]. Heating and exposure to light also lead to its decomposition [28,29]. From a pharmacological point of view, CUR is a non-specific and pleiotropic compound [53,54]. Thus, CUR in unmodified form should not be taken into consideration as a drug [55].

**Scheme 3:**
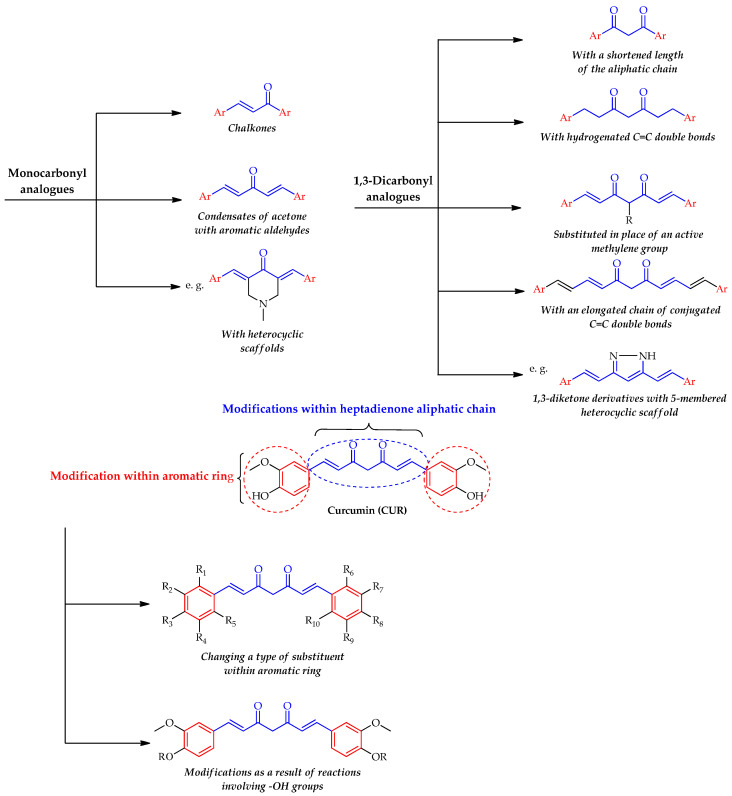
Types of analogs of CUR.

Despite the many limitations of a usage of natural CUR as a drug, new methods are still being sought to apply CUR in its more bioavailable form for pharmacological studies. The most appropriate way to eliminate the disadvantages of CUR is to synthesize numerous analogs of CUR [56]. Chemical modifications of the CUR molecule can be divided into two major groups. These include modifications within the aliphatic chain and/or the aromatic rings (Scheme 3).

**Scheme 4:**
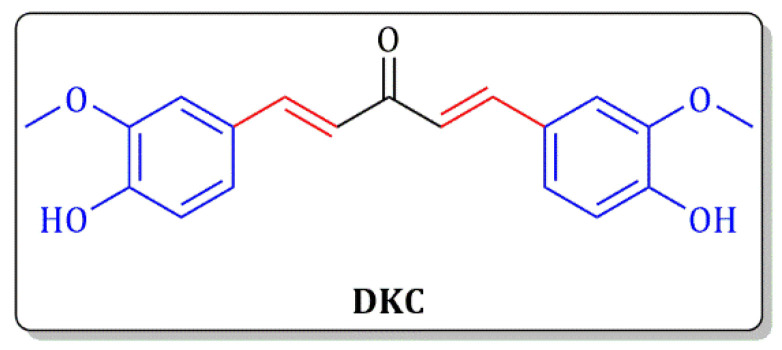
Structure of decetocurcumin (DKC).

Monocarbonyl analogs of CUR are more chemically stable and easily synthetized in comparison to its corresponding 1,3-diketones [34,57]. One of the compounds formed during the thermal decomposition of CUR is decetocurcumin (DKC) (Scheme 4), which is a monocarbonyl analog of CUR. A cell viability test proved that DKC induced the apoptosis of B78H1 murine melanoma cells [33]. Moreover, DKC protected cells against deposition of harmful *β*-amyloid and it reduced allergy induced by mast cells. The studies indicate that CUR analogs may display at least similar bioactivity without the numerous disadvantages of CUR [56].

In this study, monocarbonyl analogs of CUR based on the 9-methyl-9-azabicyclo[3.2.1]nonan-3-one (granatanone, pseudopelletierine, **1**) scaffold were synthesized. The compounds obtained by us are structurally similar to DKC. The synthesis of ten unknown monocarbonyl analogs of CUR with an improved solubility in polar solvents, including water, were performed and the anti-inflammatory properties of the selected compounds were studied. Additionally, these compounds were characterized spectroscopically and their crystal structures were determined.

## 2. Results and Discussion

### 2.1. Synthesis of the Monocarbonyl Analogs of CUR and Their Characteristics

**Scheme 5:**
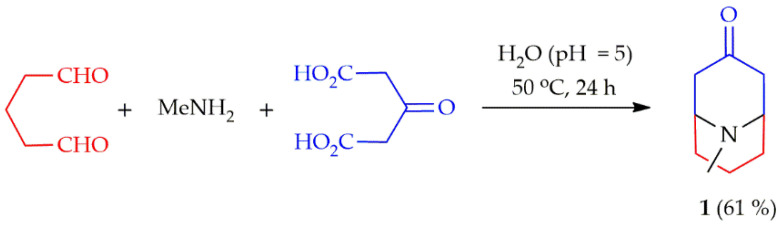
Synthesis of pseudopelletierine (**1**).

The precursor for the synthesis of the monocarbonyl analogs of CUR was 9-methyl-8-azabicyclo[3.3.1]nonan-3-one (granatanone, **1**), which was synthesized by a multi-component condensation reaction of an equimolar mixture of acetone-1,3-dicarboxylic acid, glutaraldehyde, and methylamine (Scheme 5). The reaction was carried out by heating the aqueous solution with a pH of 4–5, at the temperature of 50 °C, and for over 24 h. The appropriate reaction of the mixture was obtained by the addition of sodium acetate, which created a reaction buffer environment. The isolated crude compound was purified by crystallization followed by vacuum distillation in a Kugelrohr apparatus. The crude compound **1** easily crystallized from toluene, and its low melting point (54 °C) allowed for the solvent-free transfer of the compound in the form of a liquid. This facilitated the subsequent use of compound **1** in water-free conditions and contamination-sensitive reactions [58]. The reaction used for a single-step synthesis of compound **1** was discovered by Robinson in 1917 [59], and it is currently the most popular method of constructing the tropane skeleton and its granatanone homologue [60]. The advantage of compound **1** as a starting material for the synthesis of pharmaceuticals is its moderate toxicity to humans [61], the possibility of effective purification without the use of chromatographic methods, and the ability to carry out the synthesis without the use of transition metal compounds. These benefits increase the attractiveness of aza-bicyclic derivatives in the selection of compounds that may become drug components from among the considered candidates with similar treatment efficacy.

**Scheme 6:**
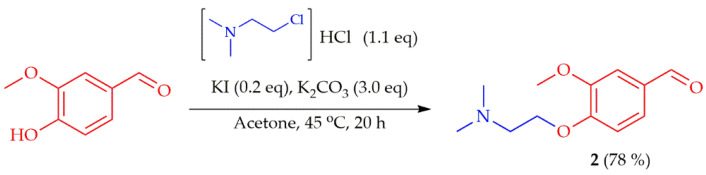
Synthesis of aldehyde (**2**).

The next step in the preparation of the monocarbonyl analogs of CUR was to perform a series of Claisen–Schmidt aldol condensation reactions with a number of aromatic aldehydes [57]. A condensation reaction between compound **1** and an excess of the aromatic aldehyde was carried out in a water–ethanol medium. A 1 M NaOH aqueous solution was used as the catalyst. After two days, the corresponding 2,6-bis(benzylidene)granatanone derivatives were isolated and purified. The synthesis of aldehyde **2** was performed according to the literature procedure (Scheme 6) [62]. The reaction consisted of a typical *S_N_*2 substitution, and iodide was used as a typical reagent in Finkleinstein reactions [63]. The small amounts of aldehyde **2** impurities prevented it from solidifying. This aldehyde was obtained in solid form by purification by chromatography or by distillation in a Kugerlohr apparatus (160 °C, 133 Pa).

**Scheme 7:**
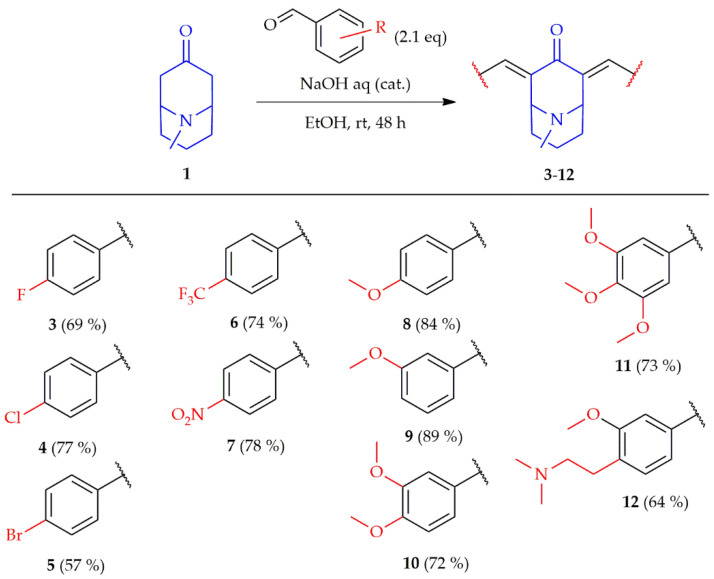
Synthesis of 2,6-bis(benzylidene)pseudopelletierine derivatives.

During the course of the reaction, compounds **3**–**11** separated out of the reaction mixture as precipitates, which were filtered and purified by crystallization. In some cases, the phenomenon of co-crystallization of impurities was observed (compounds **7** and **11**). In order to obtain products of good purity, these compounds were purified by crystallization, first from ethyl acetate or toluene and then from ethanol. In most cases, the crude reaction products were purified by chromatography. Finally, compounds **3**–**12** were obtained in moderate-to-good (57–89%) yields (Scheme 7). Compound **12**, especially its citrate salt, is highly soluble in water. The salt of compound **12** was purified by crystallization from EtOH/H_2_O (1/1; *v*/*v*). All obtained compounds were water soluble in the form of their corresponding hydrochlorides.

**Scheme 8:**
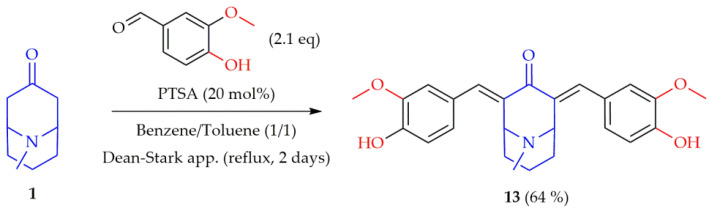
Synthesis of compound **13** containing free -OH groups.

In order to obtain the monocarbonyl analog of CUR containing -OH groups in the aromatic rings, a condensation reaction between compound **1** and vanillin was performed (Scheme 8). This reaction was not influenced by base catalysis (NaOH). The reason was probably a very low electrophilicity of the formed phenolate salt of the aldehyde. The condensation reaction was unsuccessful using SOCl_2_ in anhydrous EtOH [64], molecular iodine (I_2_) in DCM at room temperature [65], Cu(OTf)_2_ in solvent-free conditions at 80 °C [66], and NaOAc in solvent-free conditions [67]. Attempts were also made to heat compound **1** with vanillin under microwave irradiation at 120 °C in trifluoroacetic acid, unfortunately also without success. Compound **13** was successfully obtained by heating compound **1** with vanillin for two days at the boiling point of the benzene/toluene mixture (1/1; *v*/*v*) with *p*-toluenesulfonic acid (PTSA) as an effective catalyst (Scheme 8).

All obtained compounds (**3**–**11**), except compounds **12** and **13**, were poorly soluble in water. In contrast, all the obtained monocarbonyl analogs of CUR (**3**–**13**) were readily soluble in water when they were in the form of salt, e.g., hydrochloride of the corresponding amine. Moreover, all compounds (**3**–**13**) were well soluble in an acetate buffer. The highly soluble form of compound **12** is its citrate or trihydrochloride salt. Compound **12** as citrate salt can be obtained by the crystallization of an equimolar mixture amine and citric acid from an EtOH/H_2_O solution (1/1; *v*/*v*). Compound **12** as trihydrochloride salt was prepared by saturating an ethereal-layer-free amine solution with hydrogen chloride, followed by the crystallization of the precipitated salt from hot isopropanol. Attempts to obtain the crystalline form of compound **12** as sulfate (VI) salt were unsuccessful. The obtained trihydrochloride has excellent water solubility (Table S2).

**Figure 1:**
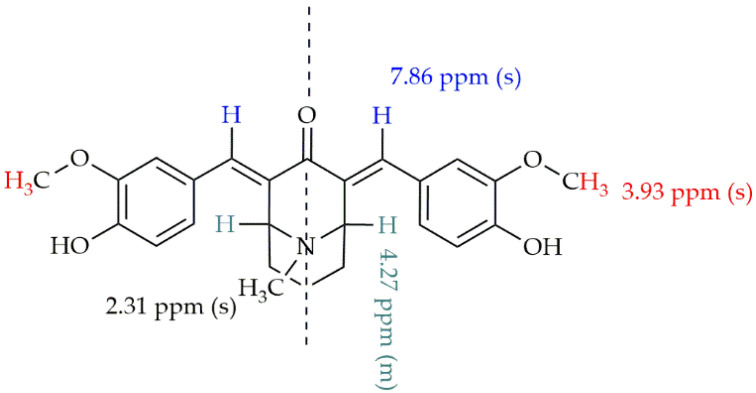
Characteristic signals on the ^1^H NMR spectrum of compound **13**.

**Figure 2:**
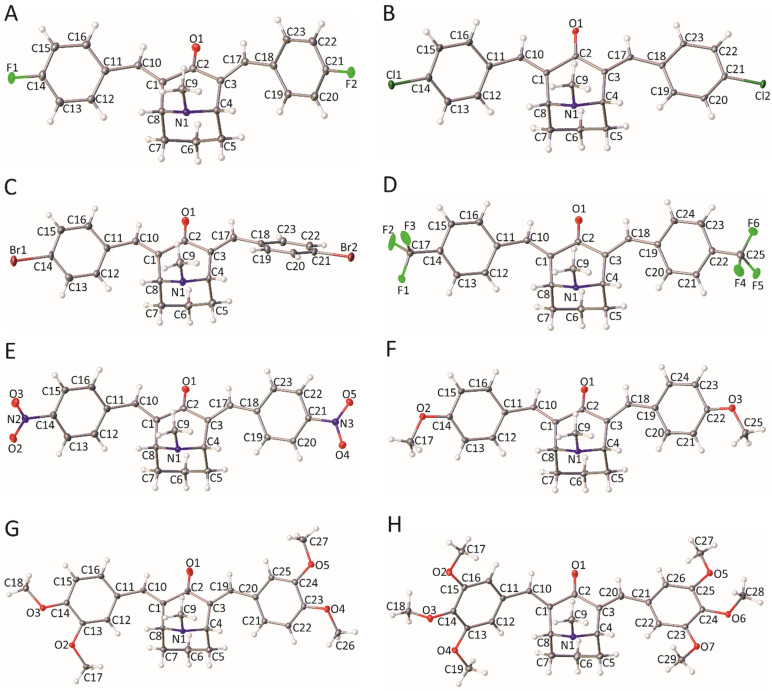
The molecular structure of compounds (**A**) **3**, (**B**) **4**, (**C**) **5**, (**D**) **6**, (**E**) **7**, (**F**) **8**, (**G**) **10**, and (**H**) **11**. Displacement ellipsoids are drawn at the 30% probability level. For clarity, for (**D**) compound **6** only, the more populated location of the disordered fluorine atoms, and, for (**E**) only, one of the total two molecules of compound **7** present in the asymmetric unit are shown.

The structures of the obtained compounds were confirmed by NMR spectroscopy and X-ray crystallography analysis. Representative characteristic signals of the ^1^H NMR spectrum are presented on Figure 1, based on compound **13**. Crystal structures of compounds **3**–**8**, **10,** and **11** (Figure 2) and the high values of shifts of signals from vinyl atoms (8.29–7.78 ppm) indicate that all obtained compounds (**3**–**13**) have double bonds with *E* geometry. Signals originating from vinyl protons in *α*,*β*-unsaturated ketones with *Z* geometry usually show lower shift values in the range of 6.0–6.5 ppm [68].

### 2.2. Limited Cytotoxicity of Monocarbonyl Analogs of CUR, CUR, and Naproxen

**Figure 3:**
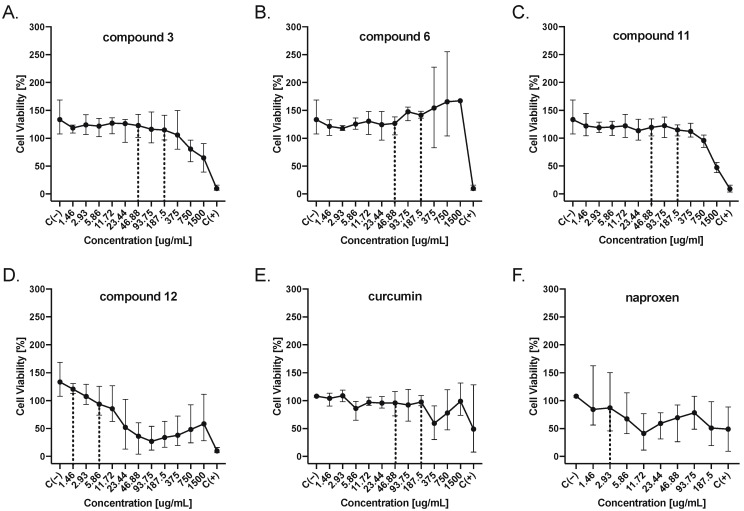
Summary of cytotoxicity analyzes and endotoxin level evaluation. (**A**–**F**) MTT assay was performed to determine cell viability. Frequency of viable cells after treatment with the monocarbonyl analogs of CUR (namely (**A**) **3**, (**B**) **6**, (**C**) **11**, and (**D**) **12**), (**E**) CUR, and (**F**) naproxen at different concentrations are presented; C(−) negative control—cells in vehicle; C(+) positive control—cells in DMSO; dashed lines—range of concentrations selected for functional experiments, *n* = 3.

First, we aimed to analyze the effects of the novel monocarbonyl analogs of CUR and CUR on the viability of peripheral blood mononuclear cells (PBMCs). To compare our results with other well-known anti-inflammatory drugs, naproxen was included in our study. We found that compounds **3**, **6**, and **11**, as well as CUR, showed no cytotoxicity in concentrations ranging from 187.5 to 1.46 μg/mL (Figure 3A–C,E). However, cells incubated with compounds **3** and **11** in the higher concentrations (in the range between 375 and 1500 μg/mL) caused decreasing leukocyte viability in a dose-dependent manner (Figure 3A,C). In contrast, compound **12** presented high cytotoxicity to peripheral blood leukocytes in concentrations ranging from 23.44 to 1500 μg/mL (Figure 3D). Additionally, naproxen showed no cytotoxicity at lower concentrations, ranging from 1.46 to 2.93 μg/mL (Figure 3F). Therefore, for functional experiments, we selected the highest concentrations with non-cytotoxic effects for peripheral blood leukocytes, namely 187.5, 93.75, and 46.88 μg/mL for compounds **3**, **6**, and **11**, and CUR; 5.86, 2.93, and 1.46 μg/mL for compound **12**; and 2,93, 1.46, and 0.73 μg/mL for naproxen.

### 2.3. Monocarbonyl Analogs of CUR, CUR, and Naproxen Solutions Possess Low Endotoxin Contamination

**Figure 4:**
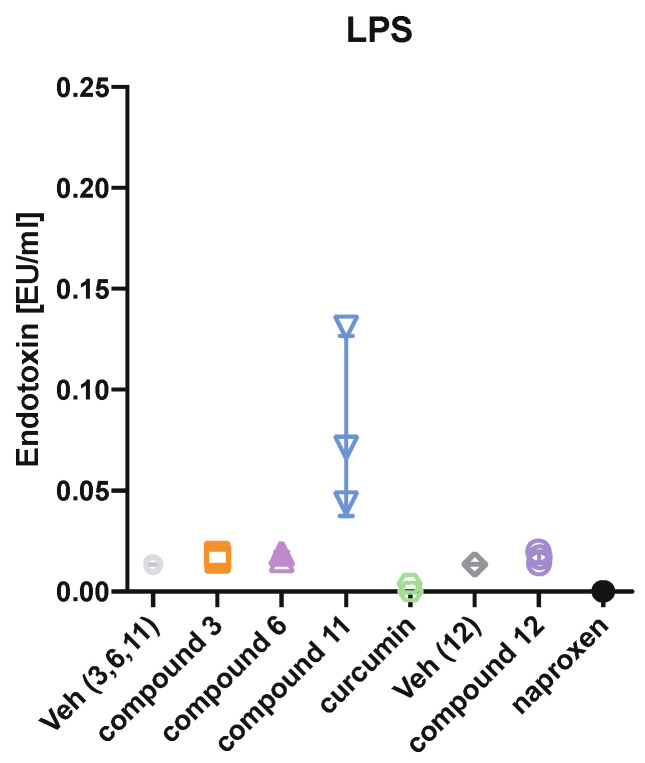
Summary of the endotoxin level evaluation in the highest non-cytotoxic concentration of selected compounds. Veh (**3**, **6**, and **11**)—solvent for compounds **3**, **6**, and **11**, and CUR; veh (**12**)—solvent for compound **12** and naproxen, *n* = 3.

Having found that analyzed monocarbonyl analogs of CUR exerted low cytotoxicity to human peripheral blood mononuclear leukocytes, we aimed to investigate endotoxin contamination in prepared solutions. Endotoxins (lipopolysaccharide, LPS) are glycolipids found in the membrane of Gram-negative bacteria, causing potent activation of the inflammatory cascade. Thus, to analyze the anti-inflammatory effects of the compounds, the endotoxin levels should be lower than 0.25 EU/mL, as admitted by the FDA for injections. In this study, we found that all analyzed compounds and their vehicles showed endotoxin levels below 0.25 EU/mL (Figure 4) in their highest non-cytotoxic concentrations. However, we observed that compound **11** has a relatively higher, but still safe, endotoxin level compared to the remaining counterparts. Therefore, all selected compounds were subjected to further analyzes.

### 2.4. Anti-Inflammatory Properties of the Monocarbonyl Analogs of CUR

**Figure 5:**
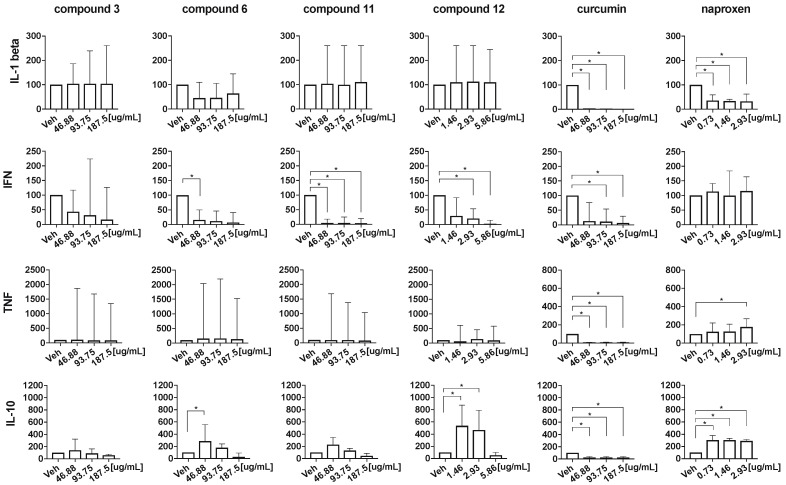
Summary of IL-1*β*, IFN-*γ*, TNF-*α*, and IL-10 analyzes in cell culture supernatants from the PBMC stimulation with LPS in the presence or absence of the monocarbonyl analogs of CUR, CUR and naproxen. PBMCs were stimulated with LPS for 24 h in the presence or absence (veh) of selected compounds (**3**, **6**, **11**, **12**, CUR, and naproxen). The levels of Il-1*β*, IFN-*γ*, TNF-*α*, and IL-10 were measured in cell culture supernatants using commercially available ELISA sets. The results are presented as a percentage of LPS stimulated control (veh) *n* = 5; the Wilcoxon test was used, * *p* < 0.05.

To assess the anti-inflammatory effects of the selected monocarbonyl analogs of CUR, we aimed to analyze their effect on cytokine secretion in a monocyte activation assay. PBMCs were activated with LPS for 24 h in the presence or absence of the selected compounds (Figure 5).

We found no significant effects of compound **3** on the secretion of proinflammatory IL-1*β*, IFN-*γ*, TNF, and anti-inflammatory IL-10 after LPS stimulation (Figure 5). In contrast, compound **6** showed a visible trend (at the threshold of statistical significance, 0.1 > *p* > 0.05) to decrease the levels of IFN-*γ* and significantly increase IL-10 levels in the lowest concentration, while no effect was observed on the levels of IL-1*β* and TNF (Figure 5). Moreover, compounds **11** and **12** significantly reduced IFN-*γ* levels; the latter compound significantly increased IL-10 production. Again, no effects of both mentioned compounds were observed on the release of IL-1*β* and TNF levels after LPS stimulation (Figure 5). Furthermore, CUR significantly decreased the level of all analyzed cytokines, both proinflammatory IL-1*β*, IFN-*γ*, TNF, and anti-inflammatory IL-10, in all analyzed concentrations. In contrast, naproxen significantly reduced IL-1*β* levels and significantly increased IL-10 levels in all analyzed concentrations. Furthemore, there were no significant differences in IFN-*γ* levels, while TNF was upregulated only at a 2.93 μg/mL concentration (Figure 5).

Having found the anti-inflammatory effect of the selected compounds on LPS stimulated cells, we aimed to investigate their effect on mitogen-stimulated PBMCs. Mechanistically, phorbol 12-myristate 13-acetate and ionomycin (PMA-IO) provide a potent stimulation of both adaptive (T and B cells) and innate immune (monocytes, dendritic cells, and NK cells) counterparts of PBMCs, thus determining the effects of analyzed compounds on the inflammatory process induced by both adaptive and innate immune responses.

**Figure 6:**
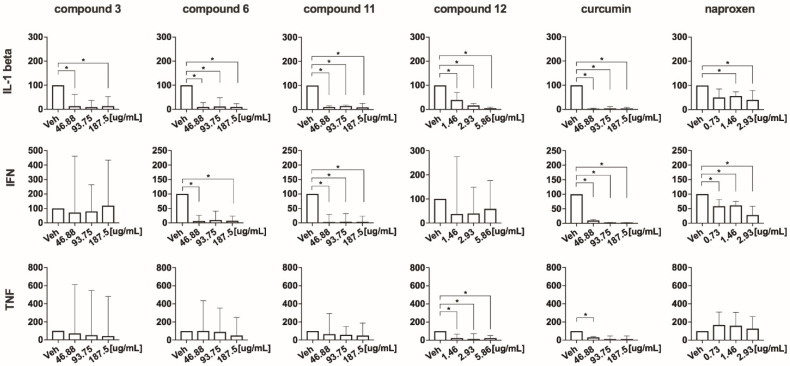
Summary of IL-*1β*, IFN-*γ*, and TNF-*α* analyzes in cell culture supernatants from PBMC stimulation with PMA-IO in the presence or absence of the monocarbonyl analogs of CUR. PBMCs were stimulated with PMA-IO for 24 h in the presence or absence (veh) of selected compounds (**3**, **6**, **11**, **12**, CUR, and naproxen). The levels of Il-*1β*, IFN-*γ*, and TNF-*α*, were measured in cell culture supernatants using commercially available ELISA sets. The results are presented as a percentage of LPS stimulated control (veh) *n* = 5; a Wilcoxon test was used, * *p* < 0.05.

First, we found that, in contrast to LPS stimulation, in mitogen stimulated cells, all analyzed monocarbonyl analogs of CUR effectively decreased the production of proinflammatory IL-1*β* (Figure 6). However, CUR and naproxen show, like in LPS, a significantly lower level of IL-1*β*, except 0.73 μg/mL for naproxen without signification (Figure 6). Moreover, compounds **6**, **11**, CUR, and naproxen, in all analyzed concentrations, effectively limited IFN-*γ* secretion from PMA-IO activated cells, while no significant effect was observed for compounds **3** and **12** (Figure 6). In addition, compound **12** decreased the release of TNF in all concentrations. CUR significantly decreased TNF in a 46.88 μg/mL concentration. Moreover, naproxen shows increased levels of TNF (Figure 6). Furthermore, no effect on IL-10 and IL-17 (data not shown) secretion was observed.

To conclude, we showed, by using two distinct in vitro models, that, from the set of the monocarbonyl analog of CUR, compound **12** is the best candidate that possess anti-inflammatory activity. However, its effectiveness in the limitation of inflammatory responses should be investigated using preclinical in vivo models.

## 3. Materials and Methods

All solvents used for FCC and reactions were purchased from POCH (Poland). Dichloromethane (DCM), ethyl acetate (AcOEt), hexane, toluene, diethyl ether (Et_2_O), and acetone were purified by fractional distillation.. All inorganic compounds were purchased from ChemPure (Poland) and used without prior purification. 2-Chloro-*N*,*N*-dimethylethylamine hydrochloride (99%), 1,3-acetonedicarboxylic acid (tech. grade), glutaraldehyde (50% wt.% in H_2_O), methylamine hydrochloride (≥98%), 4-fluorobenzaldehyde (98%), 4-chlorobenzaldehyde (97%), 4-bromobenzaldehyde (99%), 4-nitrobenzaldehyde (98%), 4-(trifluorometyl)benzaldehyde (97%), 4-methoxybenzaldehyde (≥98%), 3-methoxybenzaldehyde (≥97%), 3,4-dimethoxybenzaldehyde (99%), 3,4,5-trimethoxybenzaldehyde (98%), and vanillin (≥97%) were obtained from Merck and used without prepurification.

### 3.1. Synthesis of the Monocarbonyl Analogs of CUR

**9-Methyl-9-azabicyclo[3.3.1]nonan-3-one** (pseudopelletierine, granatanone, **1**) [69]: To a 1000 mL round bottom flask were added 300 mL of distilled water, methylamine hydrochloride (27.00 g, 0.40 mol), and 1,3-acetonedicarboxylic acid (58.44 g, 0.40 mol). The resulting solution was cooled to 0 °C in an ice bath; then glutaraldehyde (20.02 g, 0.40 mol) was added. Next, a 10% aqueous solution of CH_3_COONa was added, against the indicator paper, until the pH became approximately 5. The flask was then heated at 50 °C for 24 h. During this time, an intense evolution of carbon dioxide bubbles was observed and the reaction mixture turned slightly brown. The contents of the flask were poured into a separating funnel and acidified with 1 M HCl solution to pH 2. The resulting mixture was then washed with AcOEt (2 × 200 mL) and its combined organic layers were discarded. Next, the water phase was basified with 6 M NaOH to pH 10 and the compound was extracted with DCM (5 × 150 mL). The combined organic layers were dried with anhydrous Na_2_SO_4_, filtered through a funnel, and the solvent was removed by rotary evaporator under reduced pressure. To the dry residue, 200 mL of Et_2_O/DCM mixture (3/1; *v*/*v*) was added and the resulting solution was heated to reflux on a heating mantle (1 min). The hot mixture was then filtered through a short pad of compacted Celite on a Schott funnel, and the filtrate was concentrated on a rotary evaporator under reduced pressure. The residue was prepurified by crystallization from toluene followed by Kugelrohr distillation (100 °C, 133 Pa) to obtain the compound as a white solid (36.80 g, 61%). R_F_ [MeOH/DCM (7/95; *v*/*v*)] = 0.33; M_p_ = 53–54 °C; FTIR (ATR), *ῦ* [cm^−1^]: 1693 (C=O), 1466, 1448, 1404, 1355, 1272, 1063; ^1^H NMR (200 MHz, CDCl_3_), δ [ppm]: 3.33–3.23 (m, 2H), 2.74 (dd, *J* = 16.6, 6.6 Hz, 2H), 2.60 (s, 3H), 2.22 (d, *J* = 16.6 Hz, 2H), 2.01–1.83 (m, 2H), 1.62–1.39 (m, 4H).

**4-(2-(Dimethylamino)ethoxy)-3-methoxybenzaldehyde** (**2**) [62]: The reaction was carried out in an Ar atmosphere. To a 250 mL round bottom flask 4-hydroxy-3-methoxybenzaldehyde (3.040 g, 20 mmol), 2-chloro-*N*,*N*-dimethylethan-1-amine hydrochloride (3.169 g, 22 mmol), KI (0.664 g, 4.0 mmol), anhydrous K_2_CO_3_ (8.281 g, 60 mmol), and acetone (150 mL) were added. The contents of the flask were intensively stirred magnetically at 45 °C for 20 h (the mixture turned yellow). The solvent was then evaporated on a rotary evaporator under reduced pressure. The dry residue was transferred with distilled water (200 mL) to a separating funnel and acidified with 1 M HCl solution to pH 2. The resulting solution was washed with Et_2_O (2 × 50 mL) and the combined ethereal layers were discarded. The aqueous layer was then basified with 1 M NaOH solution to pH 10 and the compound was extracted with Et_2_O (3 × 100 mL). The combined organic layers were dried with anhydrous Na_2_SO_4_, filtered through a funnel, and the solvent was evaporated on a rotary evaporator under reduced pressure. The residue was purified by flash column chromatography [ø = 5 cm, 120 g SiO_2_, Et_3_N/EtOH/AcOEt (1/30/69; *v*/*v*/*v*)] to yield an off-white solid (3.492 g, 78%). R_F_ [Et_3_N/AcOEt/DCM (1/40/60; *v*/*v*/*v*)] = 0.52; M_p_ = 47.4–48.4 °C; FTIR (ATR), *ῦ* [cm^−1^]: 1678 (C=O), 1584, 1507, 1463, 1262, 1133, 1023; ^1^H NMR (400 MHz, CDCl_3_), δ [ppm]: 9.58 (s, 1H), 7.17 (dd, *J* = 8.2, 1.7 Hz, 1H), 7.13 (d, *J* = 1.7 Hz, 1H), 6.74 (d, *J* = 8.2 Hz, 1H), 3.93 (t, *J* = 6.0 Hz, 2H), 3.64 (s, 3H), 2.55 (t, *J* = 6.0 Hz, 2H), 2.09 (s, 6H); ^13^C NMR (101 MHz, CDCl_3_), δ [ppm]: 190.1, 153.2, 149.2, 129.5, 125.9, 111.0, 108.6, 66.7, 57.2, 55.2, 45.4.

**General procedure 1 for compounds 3-12A:** To a solution of granatanone (0.153 g, 1 mmol, compound **1**) and the corresponding aromatic aldehyde (2.1 mmol) in 95% EtOH (15 mL), the 1.3 mL of 1 M aqueous NaOH solution was added. Then, the obtained mixtures were intensively stirred magnetically for 2 days at room temperature (20–21 °C). After this time, the reaction mixtures were transferred to a separating funnel and then distilled water (150 mL) was added. The compounds were successfully extracted with AcOEt (3 × 30 mL). The combined organic layers were washed with a saturated aqueous NaCl solution (30 mL) and then dried with anhydrous Na_2_SO_4_. The dry extracts were filtered through a filter paper on a funnel and the solvents were evaporated on a rotary evaporator under reduced pressure. The dry residues were purified by flash column chromatography on silica gel or by crystallization.

**2,4-Bis((*E*)-4-fluorobenzylidene)-9-methyl-9-azabicyclo[3.3.1]nonan-3-one** (**3**) [70]: Compound **3** was prepared according to the general procedure 1 from compound **1** and 4-fluorobenzaldehyde, and purified by flash column chromatography with gradient elution [ø = 3 cm, 50 g SiO_2_, hexane → DCM/AcOEt/hexane (20/40/40; *v*/*v*/*v*)] to yield a light yellow solid (0.251 g, 69%). R_F_ [MeOH/DCM (5/95; *v*/*v*)] = 0.48; M_p_ = 161.2–162.2 °C; FTIR (ATR), *ῦ* [cm^−1^]: 1662 (C=O), 1599, 1572, 1504, 1457, 1289, 1266, 1223, 1188, 1162, 1139, 1012, 989, 866, 832; ^1^H NMR (400 MHz, CDCl_3_), δ [ppm]: 7.84 (s, 2H), 7.37 (dd, *J* = 8.5, 5.5 Hz, 4H), 7.11 (t, *J* = 8.5 Hz, 4H), 4.18–4.09 (m, 2H), 2.26 (s, 3H), 2.22–2.13 (m, 2H), 2.01–1.92 (m, 2H), 1.84–1.74 (m, 1H), 1.63–1.50 (m, 1H); ^13^C NMR (101 MHz, CDCl_3_), δ [ppm]: 189.3 (C=O), 162.9 (d, *J*_C–F_ = 250.8 Hz), 137.4, 136.4, 132.1 (d, *J*_C–F_ = 8.4 Hz), 131.2 (d, *J*_C–F_ = 3.4 Hz), 115.8 (d, *J*_C–F_ = 21.6 Hz), 57.2, 42.4, 31.1, 17.2; ^19^F NMR (377 MHz, CDCl_3_), δ [ppm]: −110.9 (s, 1F).

**2,4-Bis((*E*)-4-chlorobenzylidene)-9-methyl-9-azabicyclo[3.3.1]nonan-3-one** (**4**): Compound **4** was prepared according to the general procedure 1 from compound **1** and 4-chlorobenzaldehyde, and purified by flash column chromatography [ø = 3 cm, 80 g SiO_2_, DCM/AcOEt/hexane (20/40/40; *v*/*v*/*v*)] to yield a light yellow solid (0.280 g, 77%). R_F_ [MeOH/DCM (5/95; *v*/*v*)] = 0.69; M_p_ = 154.0–155.0 °C; FTIR (ATR), *ῦ* [cm^−1^]: 1660 (C=O), 1597, 1569, 1488, 1458, 1294, 1261, 1226, 1177, 1134, 1079, 979; ^1^H NMR (400 MHz, CDCl_3_), δ [ppm]: 7.79 (s, 2H), 7.37 (d, *J* = 8.5 Hz, 4H), 7.29 (d, *J* = 8.5 Hz, 4H), 4.14–4.06 (m, 2H), 2.23 (s, 3H), 2.18–2.11 (m, 2H), 1.97–1.90 (m, 2H), 1.79–1.73 (m, 1H), 1.58–1.47 (m, 1H); ^13^C NMR (101 MHz, CDCl_3_), δ [ppm]: 189.1 (C=O), 137.3, 137.1, 134.9, 133.5, 131.3, 128.9, 57.1, 42.3, 31.1, 17.1.

**2,4-Bis((*E*)-4-bromobenzylidene)-9-methyl-9-azabicyclo[3.3.1]nonan-3-one** (**5**): Compound **5** was prepared according to the general procedure 1 from compound **1** and 4-bromoenzaldehyde, and purified by crystallization from toluene to yield a yellow solid (0.278 g, 57%). R_F_ [MeOH/DCM (5/95; *v*/*v*)] = 0.70; M_p_ = 210.0–211.0 °C (decomposition); FTIR (ATR), *ῦ* [cm^−1^]: 1065 (C=O), 1588, 1513, 1460, 1408, 1342, 1265, 1142, 1100, 1084, 1007, 986, 929, 901, 863; ^1^H NMR (400 MHz, CDCl_3_), δ [ppm]: 8.28 (d, *J* = 8.3 Hz, 4H), 7.78 (s, 2H), 7.71 (d, *J* = 8.3 Hz, 4H), 4.14–4.01 (m, 2H), 2.19 (s, 3H), 2.11–2.01 (m, 2H), 1.89–1.79 (m, 2H), 1.77–1.69 (m, 1H), 1.40–1.22 (m, 1H). ^13^C NMR (101 MHz, CDCl_3_), δ [ppm]: 182.1, 140.2, 135.1, 130.9, 123.6, 56.3, 30.0.

**9-Methyl-2,4-bis((*E*)-4-(trifluoromethyl)benzylidene)-9-azabicyclo[3.3.1]nonan-3-one** (**6**): Compound **6** was prepared according to the general procedure 1 from compound **1** and 4-(trifluorometylo)benzaldehyde, and purified by flash column chromatography [ø = 3 cm, 60 g SiO_2_, AcOEt/hexane (40/60; *v*/*v*)] to yield a light yellow solid (0.345 g, 74%). R_F_ [MeOH/DCM (5/95; *v*/*v*)] = 0.50; M_p_ = 199.0–200.0 °C; FTIR (ATR), *ῦ* [cm^−1^]: 1662 (C=O), 1602, 1581, 1494, 1446, 1298, 1262, 1228, 1187, 1144, 1085, 1026, 983; ^1^H NMR (400 MHz, CDCl_3_), δ [ppm]: 7.86 (s, 2H), 7.68 (d, *J* = 8.3 Hz, 4H), 7.48 (d, *J* = 8.2 Hz, 4H), 4.13–4.07 (m, 2H), 2.25 (s, 3H), 2.21–2.14 (m, 2H), 1.99 1.93 (m, 2H), 1.85–1.80 (m, 1H), 1.61–1.52 (m, 1H); ^13^C NMR (101 MHz, CDCl_3_), δ [ppm]: 189.3 (C=O), 139.0, 138.6, 136.8, 130.6 (q, *J*_C–F_ = 32.8 Hz), 130.1, 125.6 (q, *J*_C–F_ = 3.7 Hz), 123.8 (q, *J*_C–F_ = 272.2 Hz), 57.2, 42.3, 31.2, 17.1.

**9-Methyl-2,4-bis((*E*)-4-nitrobenzylidene)-9-azabicyclo[3.3.1]nonan-3-one** (**7**): Compound **7** was prepared according to the general procedure 1 from compound **1** and 4-nitrobenzaldehyde, and purified by flash column chromatography [ø = 3 cm, 50 g SiO_2_, DCM/AcOEt/hexane (10/30/60; *v*/*v*/*v*)] to yield an orange solid (0.328 g, 78%). R_F_ [MeOH/DCM (5/95; *v*/*v*)] = 0.67; M_p_ = 173.0–174.0 °C; FTIR (ATR), *ῦ* [cm^−1^]: 1664 (C=O), 1603, 1574 (NO_2_), 1482, 1436, 1400, 1297, 1263, 1181, 1141, 1070, 1007; ^1^H NMR (400 MHz, CDCl_3_), δ [ppm]: 7.77 (s, 2H), 7.53 (d, *J* = 8.4 Hz, 4H), 7.23 (d, *J* = 8.4 Hz, 4H), 4.13–4.06 (m, 2H), 2.23 (s, 3H), 2.19–2.09 (m, 2H), 1.96–1.90 (m, 2H), 1.80–1.74 (m, 1H), 1.58–1.48 (m, 1H); ^13^C NMR (101 MHz, CDCl_3_), δ [ppm]: 189.2, 137.5, 137.2, 133.9, 131.9, 131.5, 123.3, 57.1, 42.3, 31.1, 17.1.

**4-Bis((*E*)-4-methoxybenzylidene)-9-methyl-9-azabicyclo[3.3.1]nonan-3-one** (**8**): Compound **8** was prepared according to the general procedure 1 from compound **1** and 4-methoxybenzaldehyde, and purified by flash column chromatography [ø = 3 cm, 50 g SiO_2_, EtOH/AcOEt (5/95; *v*/*v*)] to yield a light yellow solid (0.325 g, 84%). R_F_ [MeOH/DCM (5/95; *v*/*v*)] = 0.41; M_p_ = 194.0–195.0 °C; FTIR (ATR), *ῦ* [cm^−1^]: 1661 (C=O), 1593, 1508, 1453, 1294, 1251, 1020; ^1^H NMR (400 MHz, CDCl_3_), δ [ppm]: 7.86 (s, 2H), 7.37 (d, *J* = 8.7 Hz, 4H), 6.93 (d, *J* = 8.7 Hz, 4H), 4.25–4.15 (m, 2H), 3.83 (s, 6H), 2.27 (s, 3H), 2.20–2.11 (m, 2H), 2.04–1.95 (m, 2H), 1.81–1.72 (m, 1H), 1.63–1.52 (m, 1H); ^13^C NMR (101 MHz, CDCl_3_), δ [ppm]: 189.1 (C=O), 160.1, 138.1, 134.4, 132.2, 127.8, 114.1, 57.2, 55.2, 42.5, 31.0, 17.3.

**4-Bis((*E*)-3-methoxybenzylidene)-9-methyl-9-azabicyclo[3.3.1]nonan-3-one** (**9**): Compound **9** was prepared according to the general procedure 1 from compound **1** and 3-methoxybenzaldehyde, and purified by flash column chromatography [ø = 3 cm, 50 g SiO_2_, EtOH/AcOEt (5/95; *v*/*v*] to yield a light yellow solid (0.341 g, 89%). R_F_ [MeOH/DCM (5/95; *v*/*v*)] = 0.43; M_p_ = 164.0–165.0 °C; FTIR (ATR), *ῦ* [cm^−1^]: 1661 (C=O), 1593, 1508, 1454, 1294, 1251, 1215, 1173, 1173, 1136, 1027, 986; ^1^H NMR (400 MHz, CDCl_3_), δ [ppm]: 7.84 (s, 2H), 7.34–7.27 (m, 2H), 7.00–6.95 (m, 2H), 6.93–6.85 (m, 4H), 4.23–4.14 (m, 2H), 3.81 (s, 6H), 2.25 (s, 3H), 2.19–2.11 (m, 2H), 2.03–1.94 (m, 2H), 1.81–1.72 (m, 1H), 1.61–1.50 (m, 1H); ^13^C NMR (101 MHz, CDCl_3_), δ [ppm]: 189.6 (C=O), 159.5, 138.3, 137.1, 136.4, 129.5, 122.4, 115.4, 114.5, 57.2, 55.1, 42.3, 31.2, 17.1.

**2,4-Bis((*E*)-3,4-dimethoxybenzylidene)-9-methyl-9-azabicyclo[3.3.1]nonan-3-one** (**10**): Compound **10** was prepared according to the general procedure 1 from compound **1** and 3,4-dimethoxybenzaldehyde, and purified by flash column chromatography [ø = 3 cm, 80 g SiO_2_, EtOH/DCM/AcOEt (10/10/80; *v*/*v*/*v*)] to yield a light yellow solid (0.322 g, 72%). R_F_ [MeOH/DCM (5/95; *v*/*v*)] = 0.36; M_p_ = 138.0–139.0 °C; FTIR (ATR), *ῦ* [cm^−1^]: 1657 (C=O), 1590, 1511, 1443, 1415, 1241, 1212, 1162, 1128, 1087, 1018, 996; ^1^H NMR (400 MHz, CDCl_3_), δ [ppm]: 7.81 (s, 2H), 7.01–6.97 (m, 2H), 6.92–6.85 (m, 4H), 4.23–4.16 (m, 2H), 3.87 (s, 6H), 3.85 (s, 6H), 2.25 (s, 3H), 2.16–2.09 (m, 2H), 2.03–1.96 (m, 2H), 1.76–1.70 (m, 1H), 1.60–1.51 (m, 1H); ^13^C NMR (101 MHz, CDCl_3_), δ [ppm]: 188.7, 149.8, 148.6, 138.4, 134.5, 127.9, 123.8, 113.4, 111.0, 57.2, 55.7, 55.6, 42.5, 31.0, 17.2.

**9-Methyl-2,4-bis((*E*)-3,4,5-trimethoxybenzylidene)-9-azabicyclo[3.3.1]nonan-3-one** (**11**): Compound **11** was prepared according to the general procedure 1 from compound **1** and 1,2,3-trimethoxybenzaldehyde, and purified by flash column chromatography [ø = 3 cm, 80 g SiO_2_, EtOH/DCM/AcOEt (5/15/80; *v*/*v*/*v*)] to yield a light yellow solid (0.373 g, 73%). R_F_ [MeOH/DCM (5/95; *v*/*v*)] = 0.38; M_p_ = 191.0–192.0 °C; FT IR (ATR), *ῦ* [cm^−1^]: 1655 (C=O), 1596, 1504, 1448, 1415, 1300, 1240, 1212, 1183, 1171, 1004, 937; ^1^H NMR (400 MHz, CDCl_3_), δ [ppm]: 7.78 (s, 2H), 6.61 (s, 4H), 4.22–4.19 (m, 2H), 3.85 (s, 6H), 3.84 (s, 9H), 2.27 (s, 3H), 2.18–2.12 (m, 2H), 2.04–1.98 (m, 2H), 1.79–1.73 (m, 1H), 1.61–1.52 (m, 1H); ^13^C NMR (101 MHz, CDCl_3_), δ [ppm]: 188.8, 153.0, 138.9, 138.7, 135.7, 130.4, 107.6, 60.8, 57.3, 55.9, 42.5, 31.1, 17.1.

**2,4-Bis((*E*)-4-(2-(dimethylamino)ethoxy)-3-methoxybenzylidene)-9-methyl-9-azabicyclo[3.3.1]nonan-3-one** (**12**): Compound **12** was prepared according to the general procedure 1 from compound **1** and aromatic aldehyde **2**. After the desired period of time, the mixture was poured into a separating funnel and distilled water was added (100 mL) to it. The resulting mixture was basified with 1 M NaOH solution to pH 10. The compound was extracted with DCM (4 × 30 mL). The combined organic layers were washed with a saturated aqueous NaCl solution (1 × 30 mL) and then dried with anhydrous Na_2_SO_4_. The dry extract was filtered through a filter paper on a funnel and the solvent was evaporated on a rotary evaporator under reduced pressure. The dry residue was purified by flash column chromatography [ø = 5 cm, 50 g SiO_2_, Et_3_N/EtOH/AcOEt (1/39/60; *v*/*v*/*v*)] to yield a yellow oil (0.358 g, 64%). Compound **12** can also be purified by flash column chromatography on neutral Al_2_O_3_ using gradient elution [EtOH/DCM/AcOEt (10/10/80; *v*/*v*/*v*) → MeOH/DCM (7/93; *v*/*v*)]. R_F_ [Et_3_N/AcOEt/DCM (1/40/60; *v*/*v*/*v*)] = 0.32; FTIR (ATR), *ῦ* [cm^−1^]: 1658 (C=O), 1591, 1508, 1459, 1417, 1255, 1212, 1188, 1126, 1084, 1025, 999; ^1^H NMR (400 MHz, CDCl_3_), δ [ppm]: 7.81 (s, 2H), 6.97 (d, *J* = 8.1 Hz, 2H), 6.91 (d, *J* = 3.6 Hz, 4H), 4.20 (s, 2H), 4.12 (t, *J* = 6.0 Hz, 4H), 3.83 (s, 6H), 2.77 (d, *J* = 5.9 Hz, 4H), 2.31 (s, 12H), 2.26 (s, 3H), 2.14 (s, 3H), 2.01 (s, 3H), 1.74 (d, *J* = 13.7 Hz, 1H), 1.55 (d, *J* = 13.8 Hz, 1H); ^13^C NMR (101 MHz, CDCl_3_), δ [ppm]: 188.79, 149.12, 149.05, 138.47, 134.50, 128.21, 123.79, 113.90, 112.63, 66.91, 57.83, 57.22, 55.68, 45.82, 42.52, 31.00, 17.19.

**2,4-Bis((*E*)-4-(2-(dimethylamino)ethoxy)-3-methoxybenzylidene)-9-methyl-9-azabicyclo[3.3.1]nonan-3-one trihydrochloride (12A):** Dry HCl gas was bubbled through a 0 °C cooled solution of compound **12** (3.420 g, 6.37 mmol) in an anhydrous Et_2_O (100 mL) until it was saturated. During this time, an orange salt precipitate occurred. The resulting suspension was sonicated at the set temperature for 10 min, then the salt was filtered on a fritted funnel, and the precipitate was washed with DCM (1 × 30 mL). The crude product was crystallized from hot isopropanol (200 mL) to obtain the product as a yellow solid mp. 164.0–165.0 °C (3.724 g, 91%).

**2,4-Bis((*E*)-4-hydroxy-3-methoxybenzylidene)-9-methyl-9-azabicyclo[3.3.1]nonan-3-one** (**13**): Compound **1** [0.153 g, 1.0 mmol], 4-hydroxy-3-methoxybenzaldehyde (0.320 g, 2.1 mmol), *p*-toluenesulfonic acid [0.344 g, 2.0 mmol], and 30 mL of the benzene/toluene mixture (1/1; *v*/*v*) were placed in a 50 mL round bottom flask. A Dean–Stark trap was adapted to the flask and the reaction mixture was heated to reflux for 2 days. The progress of the reaction was monitored by thin layer chromatography on silica gel [EtOH/AcOEt/DCM (5/15/80; *v*/*v*/*v*)]. After completion of the granatanone conversion, the reaction mixture was transferred to a separating funnel and 200 mL of distilled water was added. The resulting mixture was acidified with a 5% aqueous HCl solution to a pH of about 2 and then shaken. The organic layer was discarded and the aqueous was neutralized with a 20% aqueous K_2_CO_3_ solution to pH 6 and extracted with DCM (4 × 50 mL). The combined organic layers were dried with anhydrous Na_2_SO_4_, filtered through a funnel filter, and the solvent was evaporated on a rotary evaporator under reduced pressure. The dry residue was purified by flash column chromatography [ø = 5 cm, 50 g SiO_2_, EtOH/DCM/AcOEt (5/15/80; *v*/*v*/*v*)] to yield a yellow solid (0.262 g, 62%). R_F_ [EtOH/DCM/AcOEt (5/15/80; *v*/*v*/*v*)] = 0.26; M_p_ = 162–163 °C (decomposition); FTIR (ATR), *ῦ* [cm^−1^]: 3357, 2933, 1725, 1655, 1585, 1511, 1464, 1429, 1279, 1260, 1211, 1149, 1124, 1084, 1032; ^1^H NMR (200 MHz, CDCl_3_), δ [ppm]: 7.86 (s, 2H), 6.98 (s, 4H), 6.95(s, 2H), 4.27(s, 2H), 3.93 (s, 6H), 2.31 (s, 3H), 2.21-2.00 (m, 4H), 1.76–1.56 (m, 2H); ^13^C NMR (101 MHz, DMSO-d_6_), δ [ppm]: 187.5, 148.2, 147.6, 137.8, 133.6, 126.2, 124.3, 115.9, 114.8, 56.6, 55.5, 30.1, 17.23.

**(1*E*,6*E*)-1,7-bis(4-hydroksy-3-metoksyfenylo)hepta-1,6-dien-3,5-dion (CUR):** An amount of 500 mL of DCM was added to 60 g of commercial turmeric and the suspension was shaken for a few minutes. The obtained extract was filtered through a filter funnel and the solvent was evaporated on a rotary evaporator under reduced pressure. The crude product was purified twice by FCC chromatography [ø = 5 cm, 120 g SiO_2_; MeOH/DCM (3/97; *v*/*v*)]. The fraction was crystallized from DCM at 0 °C to yield a yellow-orange solid (0.092 g) with the melting point of 180.0–181 °C (decomposition). R_F_ [MeOH/DCM (3/97; *v*/*v*)] = 0.65; ^1^H NMR (400 MHz, DMSO-d_6_), δ [ppm]: 9.66 (s, 2H), 7.54 (d, J1 = 15.8 Hz, J2 = 1.7 Hz, 2H), 7.32 (d, J = 1.7 Hz, 2H), 7.15 (dd, J1 = 8.2 Hz, J2 = 1.7 Hz, 2H), 6.82 (d, J = 8.1 Hz, 2H), 6.75 (d, J = 15.8 Hz, 2H), 6.06 (s, 1H) 3.84 (s, 6H).

### 3.2. Instruments

The retardation factor (R_F_) of compounds was calculated by tin layered chromatography (TLC). TLC were performed on aluminum plates coated with silica gel 60 F254 (Merck, Poznan, Poland). A UV-254 nm lamp and a solution of phosphoromolybdenic acid (PMA) were used as TLC stains. Flash column chromatography (FCC) was performed using 230–400 mesh silica gel (Fluka). The symbol ø means the diameter of the chromatography column [cm]. The NMR spectra were recorded on a Bruker Avance II 400 (400 MHz) or Bruker DPX 200 (200 MHz) using 5 mm probes operating at 400 MHz (200 MHz) for ^1^H NMR, and 101 MHz for ^13^C NMR (DMSO-d_6_ or CDCl_3_) and 377 MHz for ^19^F NMR (CDCl_3_) spectra. Chemical shifts were determined relative to tetramethylsilane (TMS) and referenced to the CDCl_3_ signals at δ = 7.26 ppm for ^1^H and 77.0 ppm for ^13^C spectra. The following abbreviations were used to explain the multiplicities: s (singlet), d (dublet), dd (doublet of doublets), t (triplet), q (quarted), and m (multiplet). IR spectra were recorded on a Nicolet 6700 with DTGS detector. The attenuated total reflectance (ATR) method was used. The melting point (MP) of the synthetized compounds was measured on an SRS MPA120 Ez-Melt apparatus with a temperature increase of 0.5 °C/min and this value was reported to clear point. Compound **1** was distilled using a B-585 Kugelrohr apparatus (Büchi, Flawil, Switzerland).

### 3.3. Water Solubility Test

A total of 200 mg of compounds **3**–**13** were added to 100 mL volumetric flasks each and completed to the mark with distilled water. The distilled water used for the test was previously depleted of CO_2_ by bubbling Ar through it and degassing with a water pump. The compounds were sonicated for 4 h at 40–50 °C. The obtained solutions were cooled to 20–21 °C and filtered through a membrane (Nylon, 0.22 μm). A total of 90 mL of the solution was transferred to weighed and dried 150 mL flasks. The water was evaporated on a rotary evaporator under reduced pressure (85 °C, 150 mBa). The residual water was removed by azeotroping with chloroform followed by drying the compounds in vacuo (<20 mBa, RT). The quantity of compounds was determined from the difference in the weight of the flask.

To a 5 mL vial, 2 g of **12A** salt and 2 mL of distilled water were added, and then the resulting mixture was sonicated for 1 min at 40 °C. The resulting solution was cooled to rt and left for 12 h in a closed vial. The next day, the suspension was filtered through a membrane (Nylon, 0.22 μm). An amount of 3 × 100 µL were taken from the filtrate and the solutions were weighed on an analytical balance to give the average filtrate density result of 1.26 g/mL (0.126 g; 0.125 g; 0.126 g). A total of 100 µL of each saturated compound solution was added to three weighed and dried 10 mL flasks. Then, the water was evaporated. From the difference in the weight of the flask, the weight of the compound dissolved in 100 µL of saturated **12A** solution at 20–21 °C (0.039 g; 0.039 g; 0.037 g) was calculated. It was calculated that 0.039 g of salt **12A** was dissolved in 0.087 g of H_2_O.

### 3.4. X-ray Crystallography

Single crystals of compounds **3**, **4**, **5**, **6**, **7**, **8**, **10**, and **11** were mounted on a nylon loop with paraffin oil. The X-ray diffraction data were measured at 100 K on XtalLAB Synergy (Rigaku) with Hybrid Pixel 2-dimensional detector HyPix-6000HE and Cu Kα radiation. All the data were integrated and scaled using CrysAlisPro software package (Rigaku, Neu-Isenburg, Germany). The crystal structures were solved using direct methods in the OLEX2 [71] graphical interface with SHELXT [72] and refined with SHELXL [73]. All non-hydrogen atoms were refined anisotropically by the full-matrix least-squares method. All hydrogen atoms were initially located in electron-density difference maps. Aromatic hydrogen atoms were constrained to idealized positions with C-H = 0.95–1.00 Å, with U*iso*(H) = 1.5 U*eq*(C) for methyl hydrogen atoms, and U*iso*(H) = 1.2 U*eq*(C) for other. Additionally, an analysis of *F*_o_/*F*_c_ data performed with the TwinRotMat program from the PLATON software package [74] detected a missed twinning for the data of compound **7**. Twin refinement of **7** crystal structure was performed using SHELXL, based on experimental data in HKLF5 format generated by TwinRotMat program. The PLATON software was used to validate the final crystallographic data. All crystallographic data can be obtained free of charge from The Cambridge Crystallographic Data Centre via www.ccdc.cam.ac.uk/data_request/cif. The final crystallographic data and refinement statistics for all crystal structures are reported in Table S1.

### 3.5. Peripheral Blood Mononuclear Cells Isolation

The buffy coat was collected at the Regional Center of Blood Donation and Treatment (RCKiK) in Bialystok (Poland). All samples were collected upon the approval of the Ethics Committee of the Medical University of Bialystok. A freshly obtained buffy coat was diluted 10x in cold ethylenediaminetetraacetic acid disodium salt (EDTA) and phosphate buffered saline (PBS) buffer (0.5 M EDTA, Invitrogen in PBS w/o Mg^2+^ and Ca^2+^, Corning, Burlington, Ontario, Canada) followed by peripheral blood mononuclear cell (PBMC) isolation by means of density gradient centrifugation (Pancoll, PAN Biotech). Isolated PBMCs were washed ten times in cold EDTA-PBS for 10 min in 400 g. Residual red blood cells were lysed using Pharm Lyse (Becton Dickinson Bioscience). The cells were immediately used for further experiments.

### 3.6. Endotoxin Tests: Preparation of the Monocarbonyl Analogs of CUR, CUR, and Naproxen

All tested monocarbonyl analogs of CUR were dissolved in a cell culture grade DMSO (Sigma Aldrich, Poznan, Poland) followed by ultrasonic mixing for 3 min at rt before use. Immediately after sonication, the appropriate amount of each compound was transferred to a medium to acquire a working concentration of 3000 μg/mL and mixed. The compounds were diluted in X-VIVO 10 (without Phenol red and Gentamycin, Lonza, Koln, Germany) with supplemented with a 10% fetal bovine serum (FBS, PanBiotech) for a 3-(4,5-dimetylthiazol-2-yl)-2,5-diphenyltetrazolium bromide (MTT) assay, an RPMI 1640 (PanBiotech, Aidenbach, Germany) for an endotoxin chromogenic assay, or an RPMI1640 supplemented with 10% FBS for a cytokine assay. Working concentrations of all analyzed compounds were prepared using twofold serial dilutions in an appropriate medium to obtain series of 11 dilutions (from 1.46 to 1500 − μg/mL).

### 3.7. MTT Assay

An MTT assay (Invitrogen, Eugene, OR, USA) was performed according to the manufacturer’s instructions. Briefly, the MTT reagent was prepared by dissolving 15 mg of MTT in 3 mL of sterile PBS. A solution of sodium dodecyl sulfate (SDS, Invitrogen, Eugene, OR, USA) and HCl was prepared by adding 30 mL of 0.01 M HCl to 1 mg of SDS. PBMCs with the compounds were seeded on 96-well plates (Corning, Kennebunk, ME, USA; 200,000 cells/well) and incubated for 24 h in normal conditions (37 °C, 5% CO_2_, and 96% humidity). Then, 10 μL of the 12 mM MTT was added to each well and incubated for 5 h in normal conditions. Next, 100 μL of the SDS–HCl solution was added to each well, followed by incubation for 13 h at 37 °C in a humidified chamber. Finally, the presence of a formazan derivate was analyzed at 570 nm, using a microplate reader (LedEtect 96). The cells’ viability was measured by comparing the results to the standard curve with known cell numbers (blank, 25,000, 50,000, 100,000, 200,000, and 400,000 cells/well). All measurements were performed in duplicates. The data are presented as the frequency of viable cells.

### 3.8. Endotoxin Measurements

Endotoxin levels of all analyzed compounds were examined using an established endpoint chromogenic assay method (Pierce Chromogenic Endotoxin Quant Kit, Thermo Fisher, Rockford, IL, USA), according to the manufacturer’s protocol. Briefly, 50 μL of compounds, standards, and vehicle (blank) were added in doublets on a pre-heated (37 °C) 96-well plate. Subsequently, 50 µL per well of the reconstituted amebocyte lysate reagent were added and incubated. Next, 100 µL of pre-warmed reconstituted chromogenic substrate solution were added to each reaction well and incubated for 6 min at 37 °C. Finally, 50 µL of stop solution (25% acetic acid) were added to each well and immediately analyzed using a microplate reader at 405 nm (LedEtect 96). Results were calculated according to the standard curve by MicroWin 2000 Software.

### 3.9. Cell Stimulation and Supernatant Collection

Freshly isolated PBMCs were seeded on 48-well plates (Corning, Burlington, ON, Canada) in RPMI1640 medium (PanBiotech Aidenbach, Germany,) supplemented with 10% FBS (PanBiotech, Aidenbach, Germany). Next, PBMCs were stimulated with LPS 1 µg/mL (Invivogene, San Diego, CA, USA) or phorbol 12-myristate 13-acetate (PMA) and ionomycin (2 µg/mL) (Invitrogen, Bleiswijk, Netherlands), or left unstimulated (vehicle control) in the presence or absence of monocarbonyl analogs of CUR 187.5, 93.75, and 46.88 μg/mL for compounds **3**, **6, 11**, and curcumin and 5.86, 2.93, and 1.46 μg/mL for compound **12** and 2.93, 1.46, and 0.73 μg/mL for naproxen. After a 24 h stimulation, the supernatant was collected and biobanked in −80 °C for further analysis.

### 3.10. Cytokine Assay

The levels of IL-1*β*, IL-17, IL-10, IFN-*γ*, and TNF-*α* were determined in cell culture supernatants from the 24 h PBMC stimulation using commercially available ELISA sets (R&D), according to the manufacturer’s instructions. Briefly, 96-well plates (Greiner BioOne, Frickenhausen, Germany) were coated with capture antibodies. After overnight incubation at room temperature, plates were washed with wash buffer (PBS supplemented with Tween 20, Sigma-Aldrich, Poznań, Poland). Next, plates were blocked for 1 hour at room temperature with reagent diluent (0.1% BSA, Sigma Aldrich, Poznań, Poland in PBS, Corning, Manassas, VA, USA) to prevent non-specific binding and washed 3 times with buffer. Specimens and standards were added and incubated for 2 h at rt, followed by three-time washing. Next, a mixture of detection antibodies and streptavidin-horseradish peroxidase conjugate was added and then incubated. Finally, after washing the substrate, the reaction was initiated by adding a substrate solution (TMB, Becton Dickinson Bioscience, Franklin Lakes, NJ, USA), followed by 20 min incubation and stopped using 1 M sulfuric acid solution (Avantor, Gliwice, Poland). Protein levels were analyzed using an automated light absorbance reader (LEDETEC96 system). Results were calculated according to the standard curve by MicroWin 2000 Software (MicroWin). The cytokine detection ranges were: 3.91–250 pg/mL for IL-1*β*; 9.39–600 pg/mL for IFN-*γ*; 15.6–1000 pg/mL for TNF-*α* and IL-17; and 31.3–2000 pg/mL for IL-10.

### 3.11. Statistics

Statistical analyses were performed using GraphPad Prism ver. 8 (GraphPad Software). A Wilcoxon test was used. The differences were considered significant at *p* < 0.05. The results are presented as median (interquartile range, IQR).

## 4. Conclusions

In this work, we aimed to obtain bioactive curcuminoids with improved bioavailability. In our study, we successfully synthesized monocarbonyl analogs of CUR that are based on the pseudopelletierine scaffold, and we confirmed their structures through NMR spectroscopy and X-ray crystallography methods. Most of the obtained compounds are poorly soluble in water. However, the presence of granatanone moiety ensures high solubility in water-based solutions when they are in the form of salt. Next, we evaluated the biological activity of the obtained monocarbonyl analogs of CUR. Our study revealed that these derivatives have limited cytotoxicity. Additionally, we investigated the anti-inflammatory effect of the CUR derivatives on LPS-stimulated peripheral blood mononuclear cells. We found out that compound **12** is the most promising derivative with anti-inflammatory activity. However, further analysis of its effectiveness in the limitation of inflammation needs to be investigated in preclinical in vivo models.

## Data Availability

CCDC numbers 2096435 (**3**), 2097003 (**4**), 2097005 (**5**), 2096436 (**6**), 2097179 (**7**), 2097004 (**8**), 2097006 (**10**), and 2096434 (**11**) contain supplementary crystallographic data for these compounds and can be obtained free of charge from The Cambridge Crystallographic Data Centre via www.ccdc.cam.ac.uk/data_request/cif.

## Supplementary Materials

The following are available online at https://www.mdpi.com/article/10.3390/ijms222111384/s1.
