# Supplementary material for: Monocarbonyl Analogs of Curcumin Based on the Pseudopelletierine Scaffold: Synthesis and Anti-Inflammatory Activity
Source: Int J Mol Sci. 2021 Oct 21;22(21):11384. doi: 10.3390/ijms222111384 (PMC8583854; doi:10.3390/ijms222111384)
Supplement: Supplementary file 1 [file ijms-22-11384-s001.zip › ijms-1375944-supplementary.pdf]

Supplementary Materials

Monocarbonyl Analogues of Curcumin Based on Pseudopelletierine Scaffold: Synthesis and Anti-inflammatory Activity

Damian Pawelski [1,†], Alicja Walewska [2,†], Sylwia Ksiezak [2], Dariusz Sredzinski [3], Piotr Radziwon [3,4], Marcin Moniuszko [2,5], Ramesh Gandusekar [2], Andrzej Eljaszewicz [2,*], Ryszard Lazny [6], Krzysztof Brzezinski [7,*] and Marta E. Plonska-Brzezinska [1,*]

[1] Department of Organic Chemistry, Faculty of Pharmacy with the Division of Laboratory Medicine, Medical University of Bialystok, Mickiewicza 2A, 15-222 Bialystok, Poland; damian.pawelski@umb.edu.pl (D.P.); marta.plonska-brzezinska@umb.edu.pl (M.E.P.-B.)

[2] Department of Regenerative Medicine and Immune Regulation, Medical University of Bialystok, Waszyngtona 13, 15-269 Bialystok, Poland; alicja.walewska@umb.edu.pl (A.W.); sylwia.ksiezak@icloud.com (S.K.); marcin.moniuszko@umb.edu.pl (M.M.); ramesh.gandusekar@umb.edu.pl (R.G.); andrzej.eljaszewicz@umb.edu.pl (A.E.)

[3] Regional Blood Donation and Blood Treatment Center in Bialystok, M. Sklodowskiej-Curie 23, 15-950 Bialystok, Poland; dsredzinski@rckik.bialystok.pl (D.S.); piotr.radziwon@umb.edu.pl (P.R.)

[4] Department of Hematology, Medical University of Bialystok, M. Sklodowskiej-Curie 24A, 15-276 Bialystok, Poland; piotr.radziwon@umb.edu.pl (P.R.)

[5] Department of Allergology and Internal Medicine, Medical University of Bialystok, M. Sklodowskiej-Curie 24A, 15-276 Bialystok, Poland; marcin.moniuszko@umb.edu.pl (M.M.)

[6] Faculty of Chemistry, University of Bialystok, Ciolkowskiego 1K, 15-245 Bialystok, Poland; lazny@uwb.edu.pl

[7] Department of Structural Biology of Prokaryotic Organisms, Institute of Bioorganic Chemistry, Polish Academy of Sciences, Noskowskiego 12/14, 61-074 Poznan, Poland; kbrzezinski@ibch.poznan.pl (K.B.)

* Correspondence: andrzej.eljaszewicz@umb.edu.pl (A.E.); kbrzezinski@ibch.poznan.pl (K.B.); marta.plonska-brzezinska@umb.edu.pl (M.E.P.-B.)

† These authors contributed equally to this work.

Figure S1. ^{1}H NMR: 9-Methyl-9-azabicyclo[3.3.1]nonan-3-one (**1**)
Figure S2. FTIR (ATR): 9-methyl-9-azabicyclo[3.3.1]nonan-3-one (**1**)
Figure S3. ^{1}H NMR: 4-(2-(Dimethylamino)ethoxy)-3-methoxybenzaldehyde (**2**)
Figure S4. ^{13}C NMR: 4-(2-(Dimethylamino)ethoxy)-3-methoxybenzaldehyde (**2**)
Figure S5. FTIR (ATR): 4-(2-(Dimethylamino)ethoxy)-3-methoxybenzaldehyde (**2**)
Figure S6. ^{1}H NMR: 2,4-Bis((*E*)-4-fluorobenzylidene)-9-methyl-9-azabicyclo[3.3.1]nonan-3-one (**3**)
Figure S7. ^{13}C NMR: 2,4-Bis((*E*)-4-fluorobenzylidene)-9-methyl-9-azabicyclo[3.3.1]nonan-3-one (**3**)
Figure S8. ^{19}F NMR: 2,4-Bis((*E*)-4-fluorobenzylidene)-9-methyl-9-azabicyclo[3.3.1]nonan-3-one (**3**)
Figure S9. FTIR (ATR): 2,4-Bis((*E*)-4-fluorobenzylidene)-9-methyl-9-azabicyclo[3.3.1]nonan-3-one (**3**)
Figure S10. ^{1}H NMR: 2,4-Bis((*E*)-4-chlorobenzylidene)-9-methyl-9-azabicyclo[3.3.1]nonan-3-one (**4**)
Figure S11. ^{13}C NMR: 2,4-Bis((*E*)-4-chlorobenzylidene)-9-methyl-9-azabicyclo[3.3.1]nonan-3-one (**4**)
Figure S12. FTIR (ATR): 2,4-Bis((*E*)-4-chlorobenzylidene)-9-methyl-9-azabicyclo[3.3.1]nonan-3-one (**4**)
Figure S13. ^{1}H NMR: 2,4-Bis((*E*)-4-bromobenzylidene)-9-methyl-9-azabicyclo[3.3.1]nonan-3-one (**5**)
Figure S14. ^{13}C NMR: 2,4-Bis((*E*)-4-bromobenzylidene)-9-methyl-9-azabicyclo[3.3.1]nonan-3-one (**5**)
Figure S15. FTIR (ATR): 2,4-Bis((*E*)-4-bromobenzylidene)-9-methyl-9-azabicyclo[3.3.1]nonan-3-one (**5**)
Figure S16. ^{1}H NMR: 9-Methyl-2,4-bis((*E*)-4-(trifluoromethyl)benzylidene)-9-azabicyclo[3.3.1]nonan-3-one (**6**)
Figure S17. ^{13}C NMR: 9-Methyl-2,4-bis((*E*)-4-(trifluoromethyl)benzylidene)-9-azabicyclo[3.3.1]nonan-3-one (**6**)
Figure S18. FTIR (ATR): 9-Methyl-2,4-bis((*E*)-4-(trifluoromethyl)benzylidene)-9-azabicyclo[3.3.1]nonan-3-one (**6**)
Figure S19. ^{1}H NMR: 9-Methyl-2,4-bis((*E*)-4-nitrobenzylidene)-9-azabicyclo[3.3.1]nonan-3-one (**7**)
Figure S20. ^{13}C NMR: 9-Methyl-2,4-bis((*E*)-4-nitrobenzylidene)-9-azabicyclo[3.3.1]nonan-3-one (**7**)
Figure S21. FTIR (ATR): 9-Methyl-2,4-bis((*E*)-4-nitrobenzylidene)-9-azabicyclo[3.3.1]nonan-3-one (**7**)
Figure S22. ^{1}H NMR: 4-Bis((*E*)-4-methoxybenzylidene)-9-methyl-9-azabicyclo[3.3.1]nonan-3-one (**8**)
Figure S23. ^{13}C NMR: 4-Bis((*E*)-4-methoxybenzylidene)-9-methyl-9-azabicyclo[3.3.1]nonan-3-one (**8**)

Figure S24. FTIR (ATR): 4-Bis((*E*)-4-methoxybenzylidene)-9-methyl-9-azabicyclo[3.3.1]nonan-3-one (**8**)
Figure S25. ^{1}H NMR: 4-Bis((*E*)-3-methoxybenzylidene)-9-methyl-9-azabicyclo[3.3.1]nonan-3-one (**9**)
Figure S26. ^{13}C NMR: 4-Bis((*E*)-3-methoxybenzylidene)-9-methyl-9-azabicyclo[3.3.1]nonan-3-one (**9**)
Figure S27. FTIR (ATR): 4-Bis((*E*)-3-methoxybenzylidene)-9-methyl-9-azabicyclo[3.3.1]nonan-3-one (**9**)
Figure S28. ^{1}H NMR: 2,4-Bis((*E*)-3,4-dimethoxybenzylidene)-9-methyl-9-azabicyclo[3.3.1]nonan-3-one (**10**)
Figure S29. ^{13}C NMR: 2,4-bis((*E*)-3,4-dimethoxybenzylidene)-9-methyl-9-azabicyclo[3.3.1]nonan-3-one (**10**)
Figure S30. FTIR (ATR): 2,4-bis((*E*)-3,4-dimethoxybenzylidene)-9-methyl-9-azabicyclo[3.3.1]nonan-3-one (**10**)
Figure S31. ^{1}H NMR: 9-Methyl-2,4-bis((*E*)-3,4,5-trimethoxybenzylidene)-9-azabicyclo[3.3.1]nonan-3-one (**11**)
Figure S32. ^{13}C NMR: 9-Methyl-2,4-bis((*E*)-3,4,5-trimethoxybenzylidene)-9-azabicyclo[3.3.1]nonan-3-one (**11**)
Figure S33. FTIR (ATR): 9-Methyl-2,4-bis((*E*)-3,4,5-trimethoxybenzylidene)-9-azabicyclo[3.3.1]nonan-3-one (**11**)
Figure S34. ^{1}H NMR: 2,4-Bis((*E*)-4-(2-(dimethylamino)ethoxy)-3-methoxybenzylidene)-9-methyl-9-azabicyclo[3.3.1]nonan-3-one (**12**)
Figure S35. ^{13}C NMR: 2,4-Bis((*E*)-4-(2-(dimethylamino)ethoxy)-3-methoxybenzylidene)-9-methyl-9-azabicyclo[3.3.1]nonan-3-one (**12**)
Figure S36. FTIR (ATR): 2,4-Bis((*E*)-4-(2-(dimethylamino)ethoxy)-3-methoxybenzylidene)-9-methyl-9-azabicyclo[3.3.1]nonan-3-one (**12**)
Figure S37. ^{1}H NMR: 2,4-Bis((*E*)-4-hydroxy-3-methoxybenzylidene)-9-methyl-9-azabicyclo[3.3.1]nonan-3-one (**13)**
Figure S38. ^{13}C NMR: 2,4-Bis((*E*)-4-hydroxy-3-methoxybenzylidene)-9-methyl-9-azabicyclo[3.3.1]nonan-3-one (**13**)
Figure S39. FTIR (ATR): 2,4-Bis((*E*)-4-hydroxy-3-methoxybenzylidene)-9-methyl-9-azabicyclo[3.3.1]nonan-3-one (**13**)
Figure S40. ^{1}H NMR (200 MHz, DMSO): CUR.
Figure S41. Analysis of the purity of CUR by HPLC.
Table S1. Crystal data and structure refinement details for **3**, **4**, **5**, **6**, **7**, **8**, **10** and **11** compounds.
Table S2. Solubility (S) in distilled water at 21 ± 1°C.

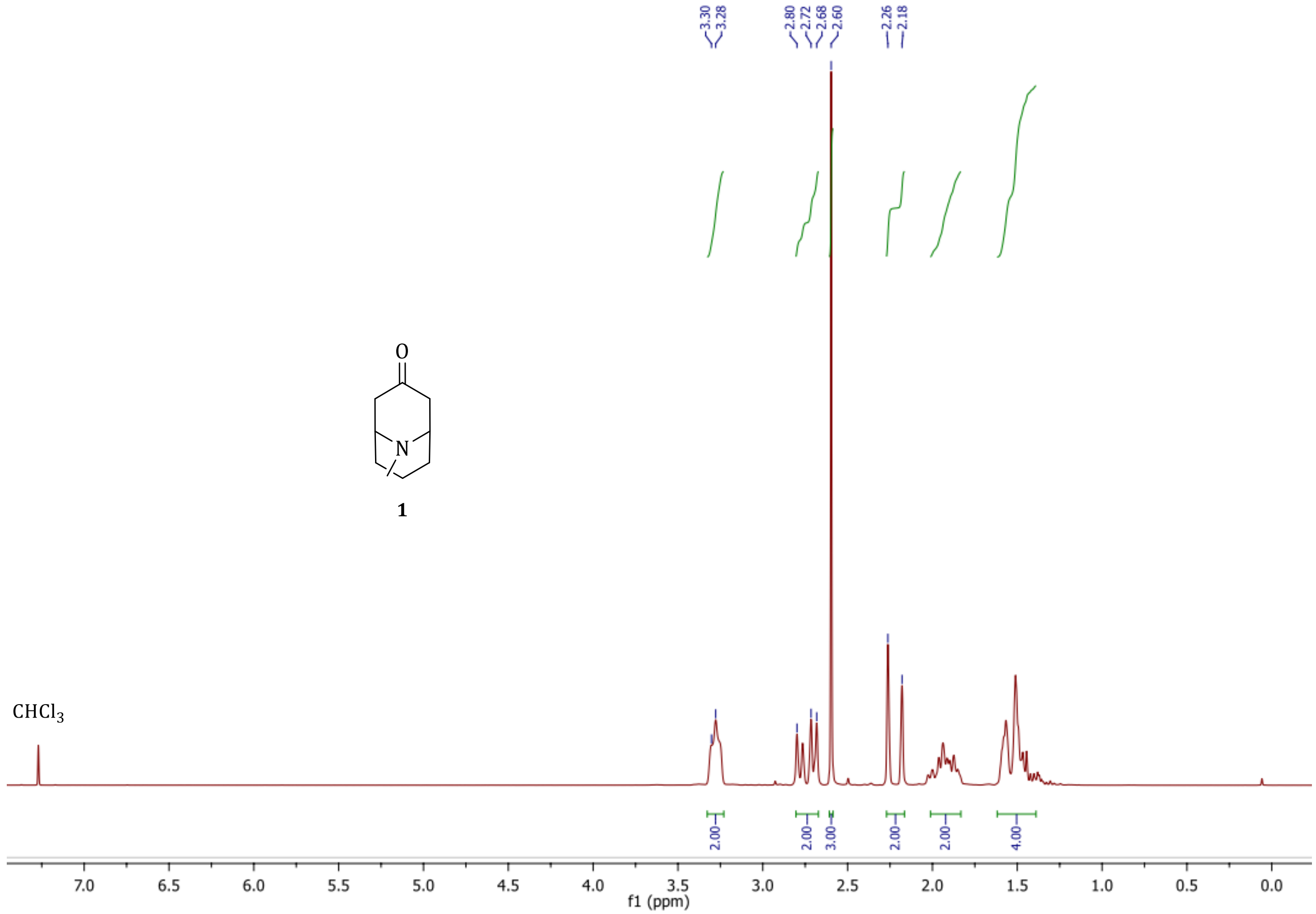

Figure S1. ^{1}H NMR (200 MHz, $CDCl_3$): 9-Methyl-9-azabicyclo[3.3.1]nonan-3-one (**1**)

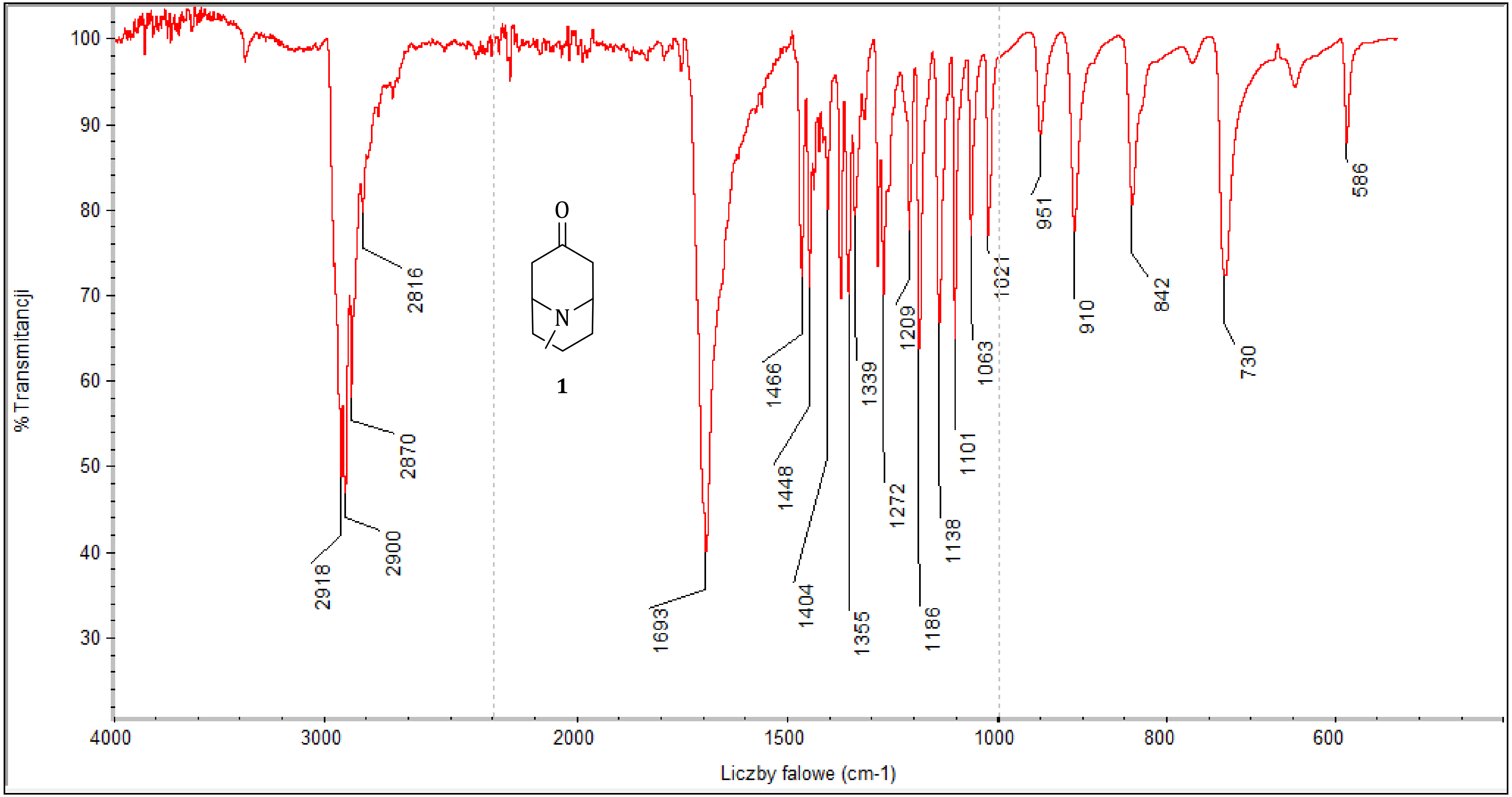

Figure S2. FTIR (ATR): 9-methyl-9-azabicyclo[3.3.1]nonan-3-one (**1**)

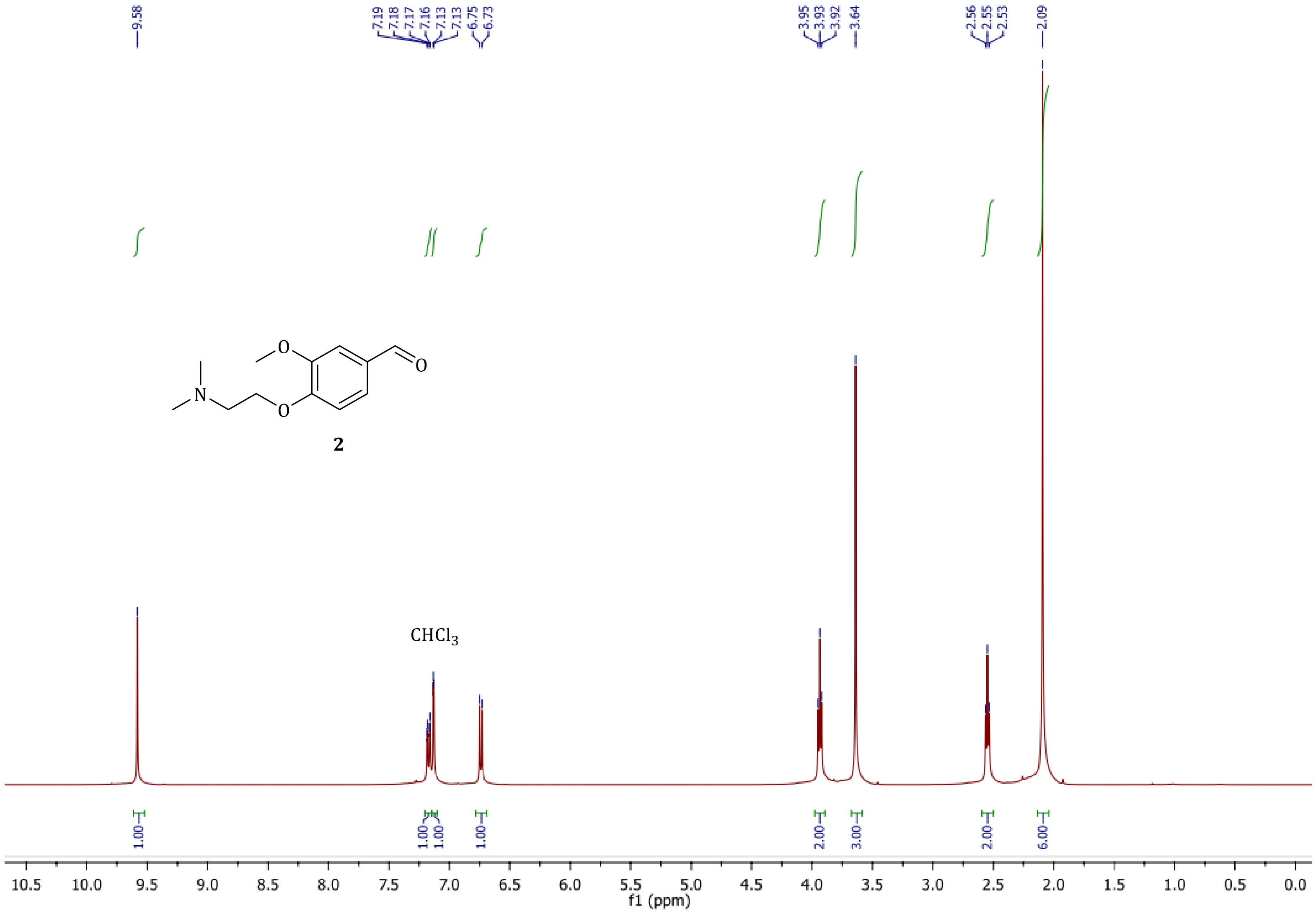

Figure S3. ^{1}H NMR (400 MHz, $CDCl_3$): 4-(2-(Dimethylamino)ethoxy)-3-methoxybenzaldehyde (**2**)

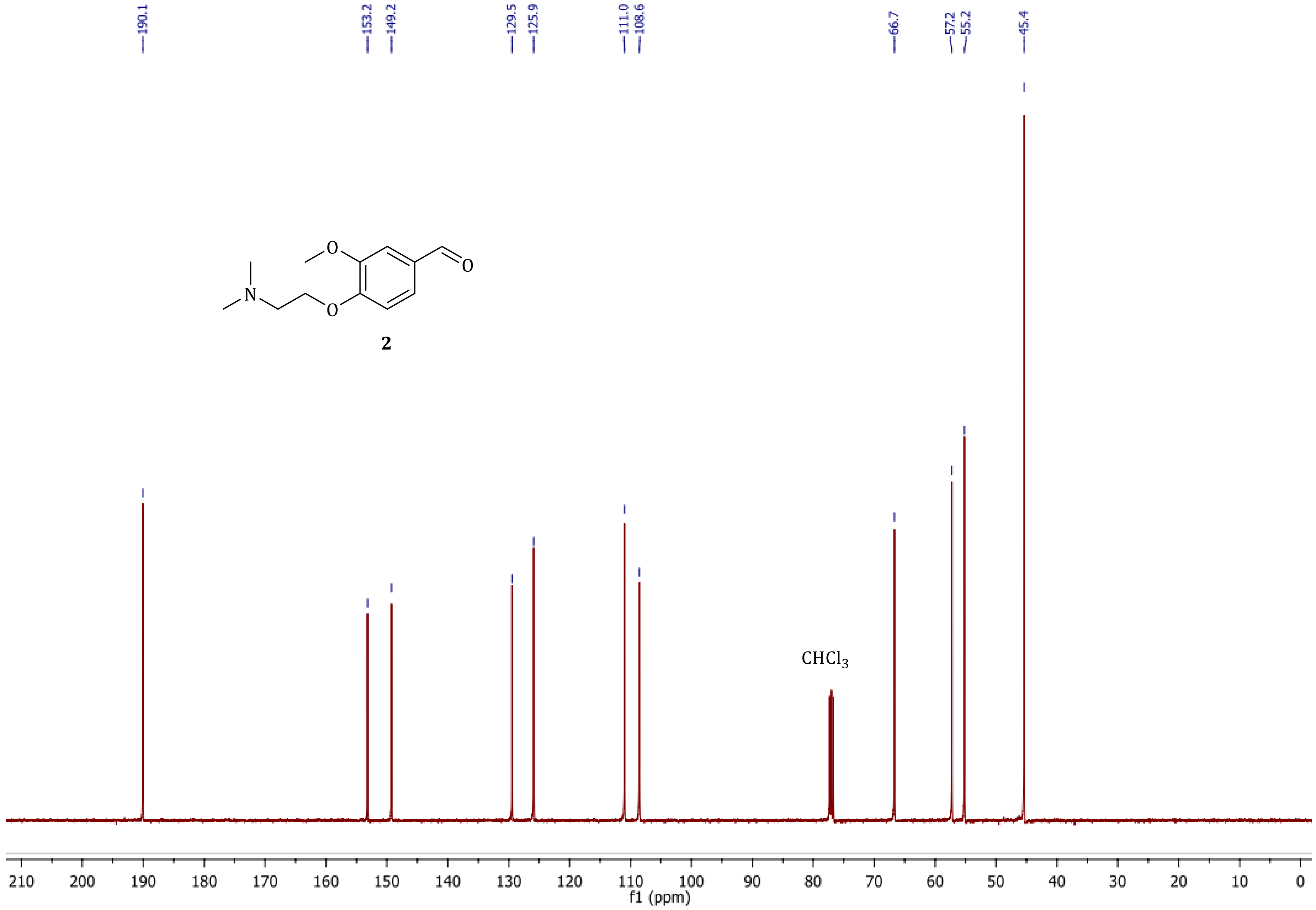

Figure S4. ^{13}C NMR (101 MHz, $CDCl_3$): 4-(2-(Dimethylamino)ethoxy)-3-methoxybenzaldehyde (**2**)

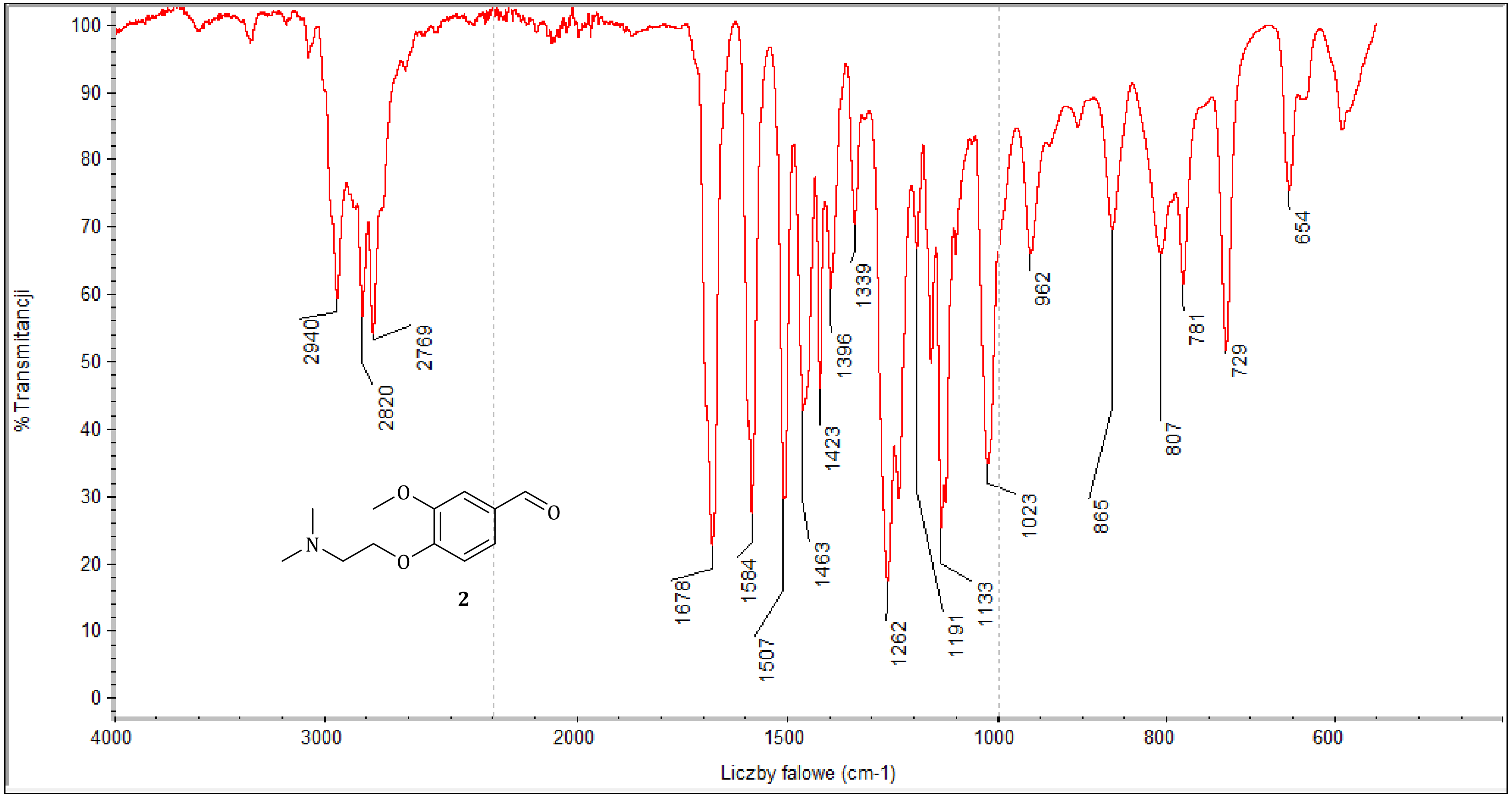

Figure S5. FTIR (ATR): 4-(2-(Dimethylamino)ethoxy)-3-methoxybenzaldehyde (**2**)

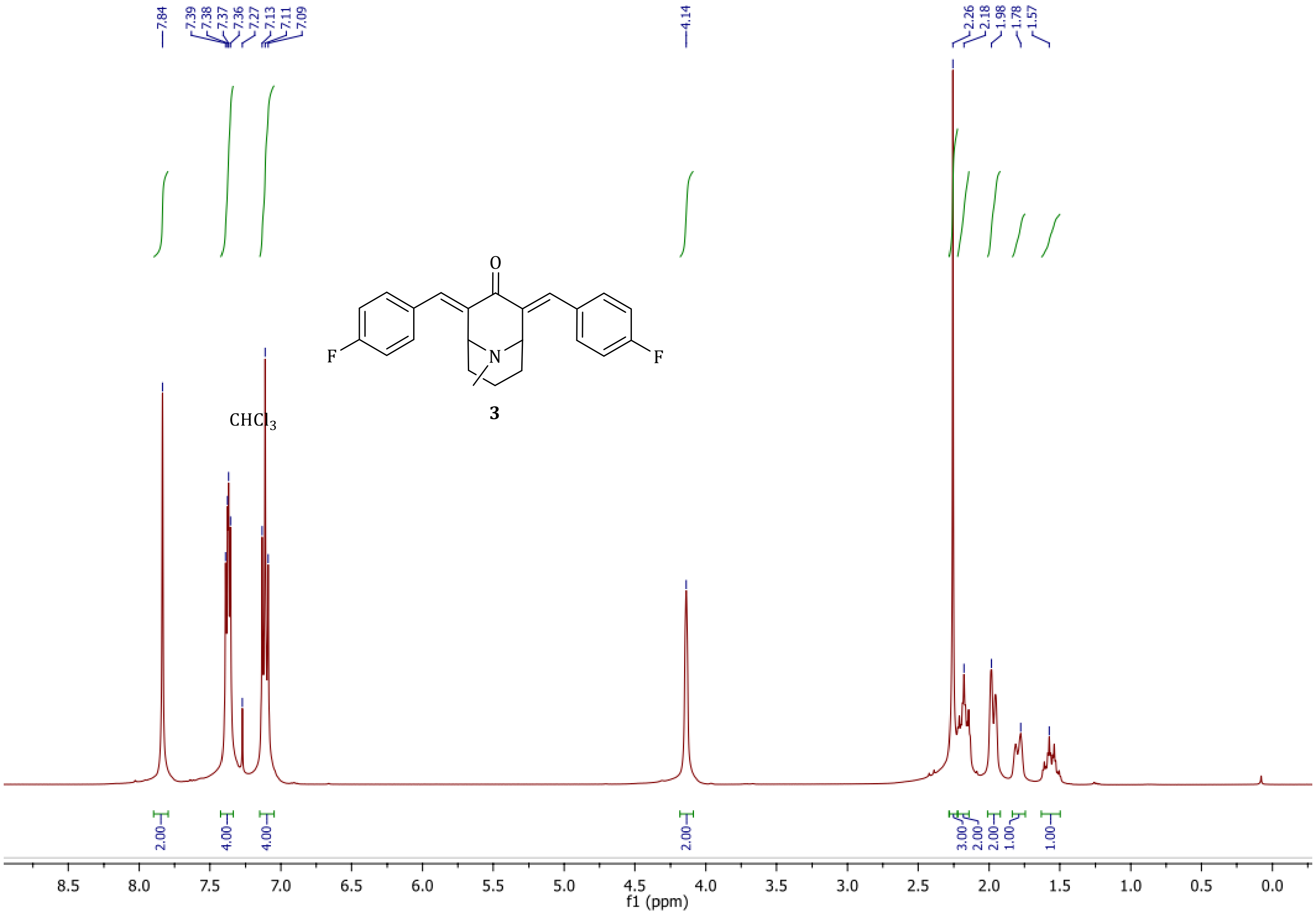

Figure S6. ^{1}H NMR (400 MHz, $CDCl_3$): 2,4-Bis((*E*)-4-fluorobenzylidene)-9-methyl-9-azabicyclo[3.3.1]nonan-3-one (**3**)

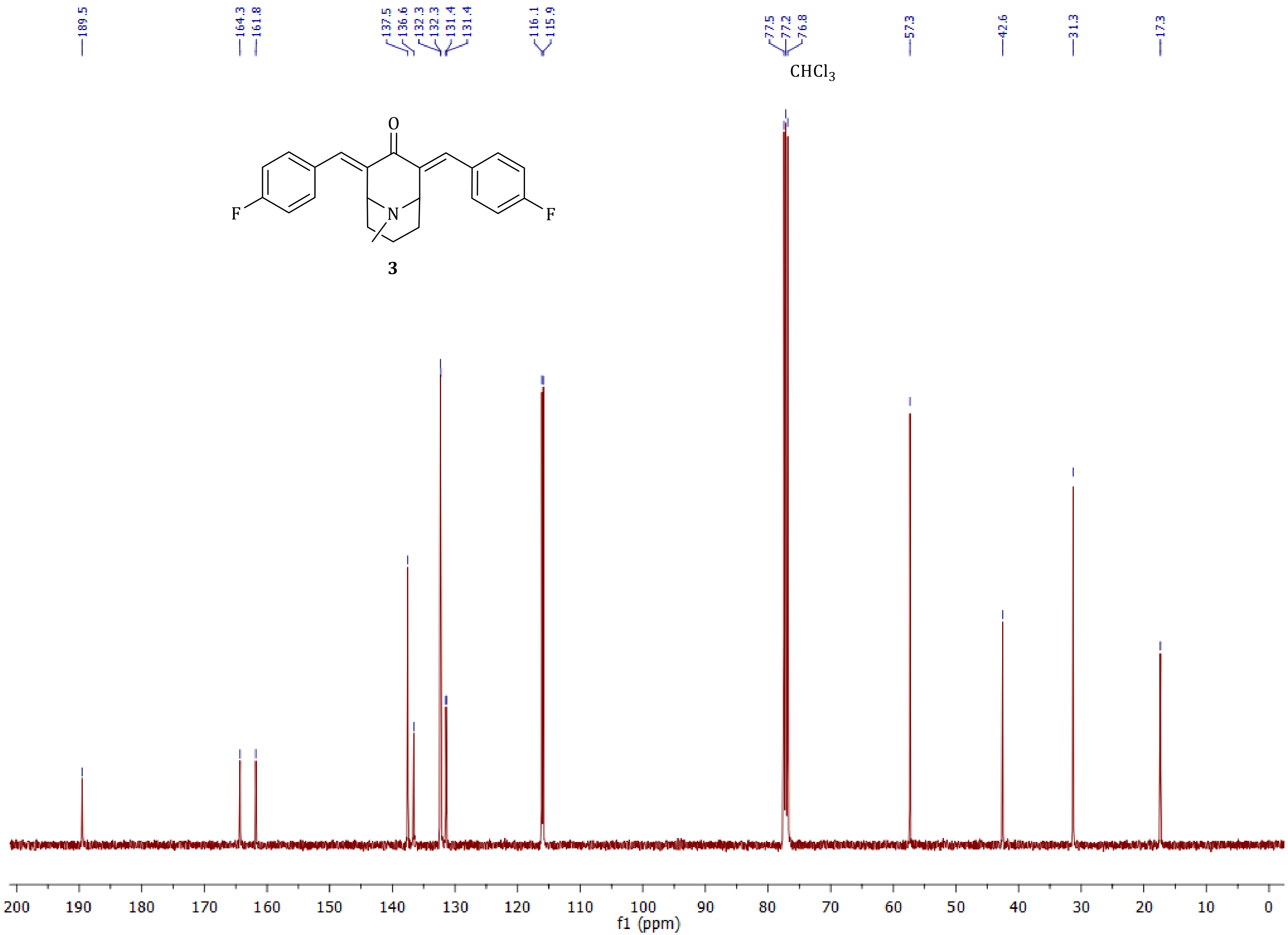

Figure S7. ^{13}C NMR (101 MHz, $CDCl_3$): 2,4-Bis((*E*)-4-fluorobenzylidene)-9-methyl-9-azabicyclo[3.3.1]nonan-3-one (**3**)

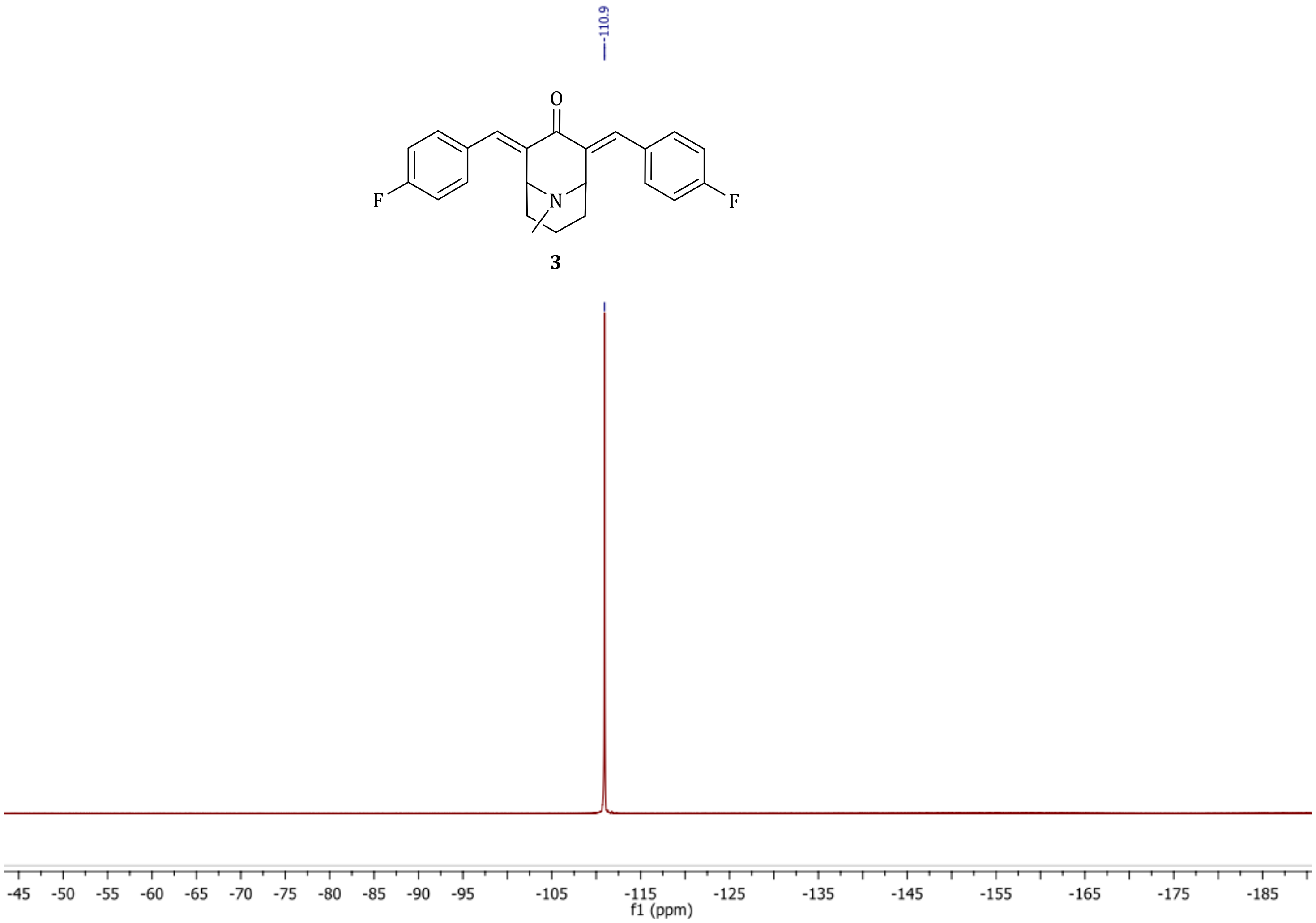

Figure S8. ^{19}F NMR (377 MHz, $CDCl_3$): 2,4-Bis((*E*)-4-fluorobenzylidene)-9-methyl-9-azabicyclo[3.3.1]nonan-3-one (**3**)

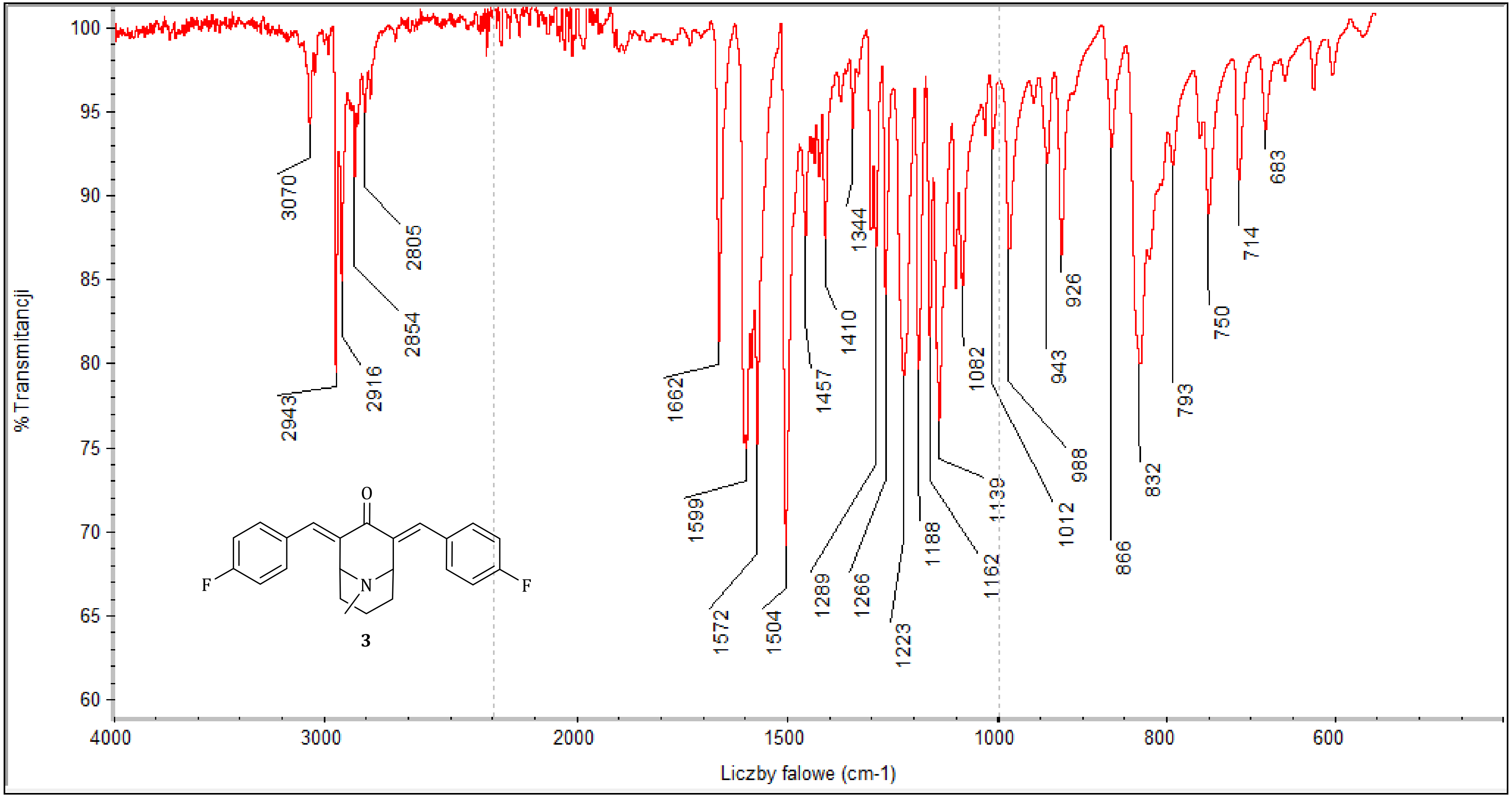

Figure S9. FTIR (ATR): 2,4-Bis((*E*)-4-fluorobenzylidene)-9-methyl-9-azabicyclo[3.3.1]nonan-3-one (**3**)

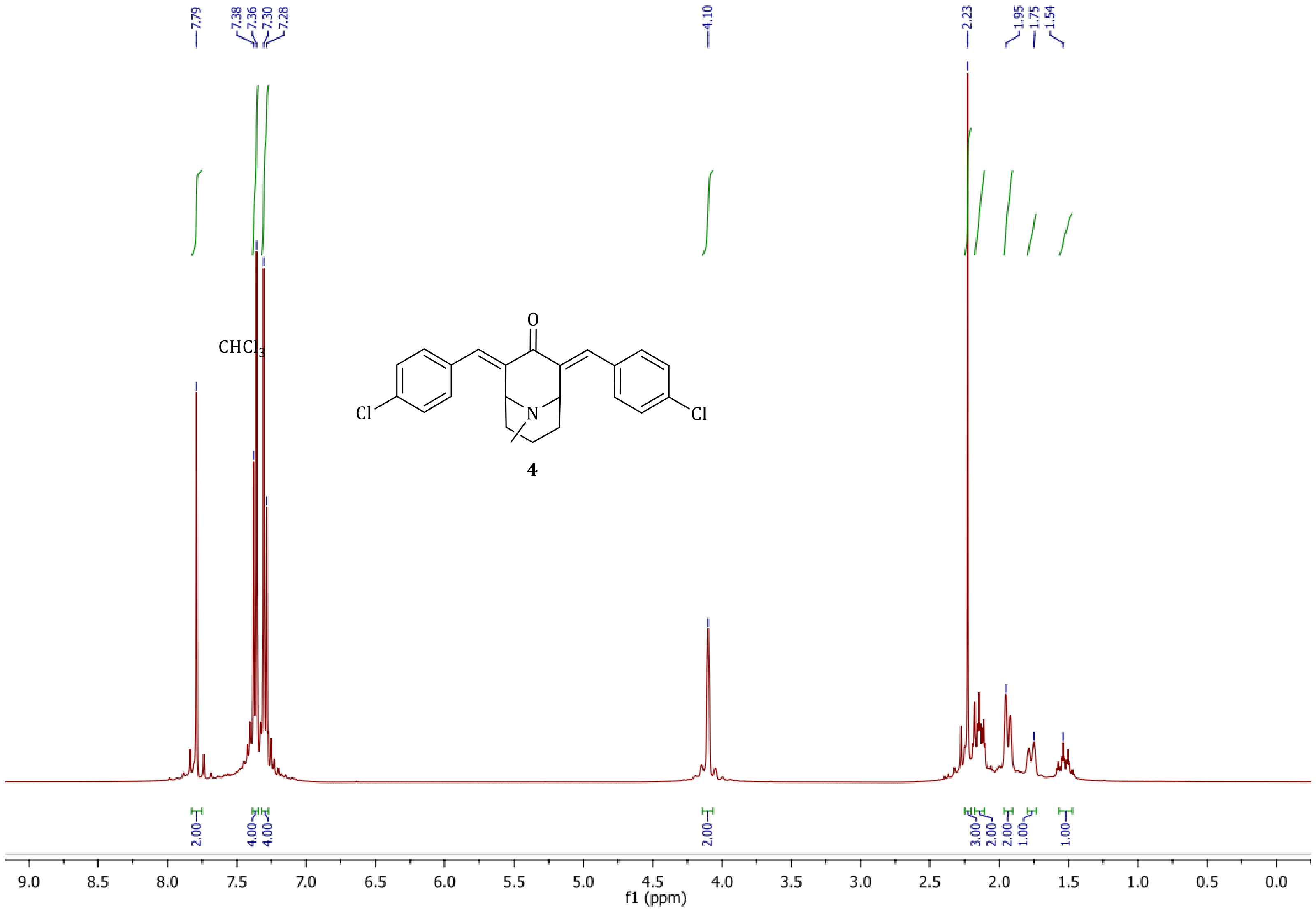

Figure S10. ^{1}H NMR (400 MHz, $CDCl_3$): 2,4-Bis((*E*)-4-chlorobenzylidene)-9-methyl-9-azabicyclo[3.3.1]nonan-3-one (**4**)

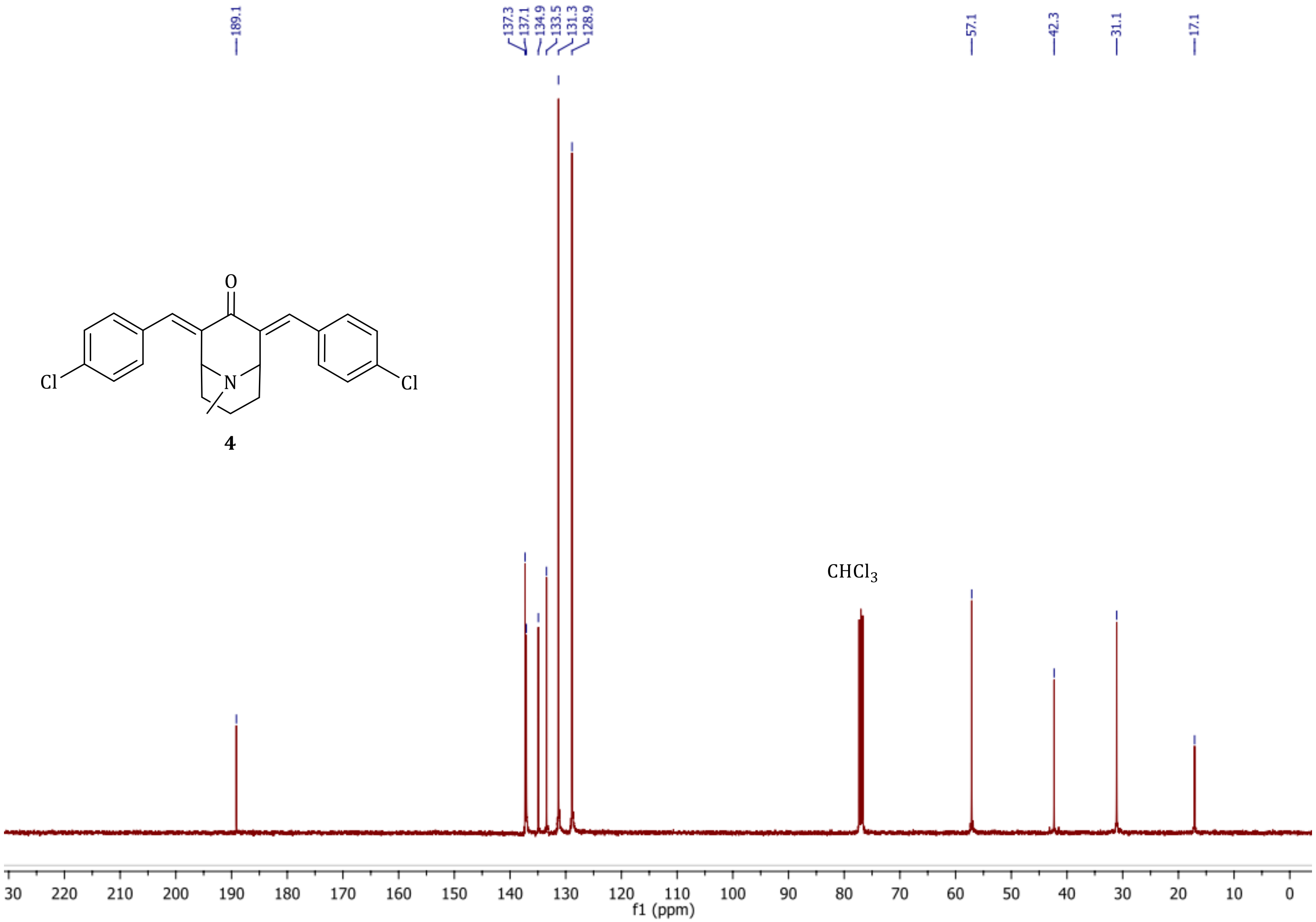

Figure S11. ^{13}C NMR (101 MHz, $CDCl_3$): 2,4-Bis((*E*)-4-chlorobenzylidene)-9-methyl-9-azabicyclo[3.3.1]nonan-3-one (**4**)

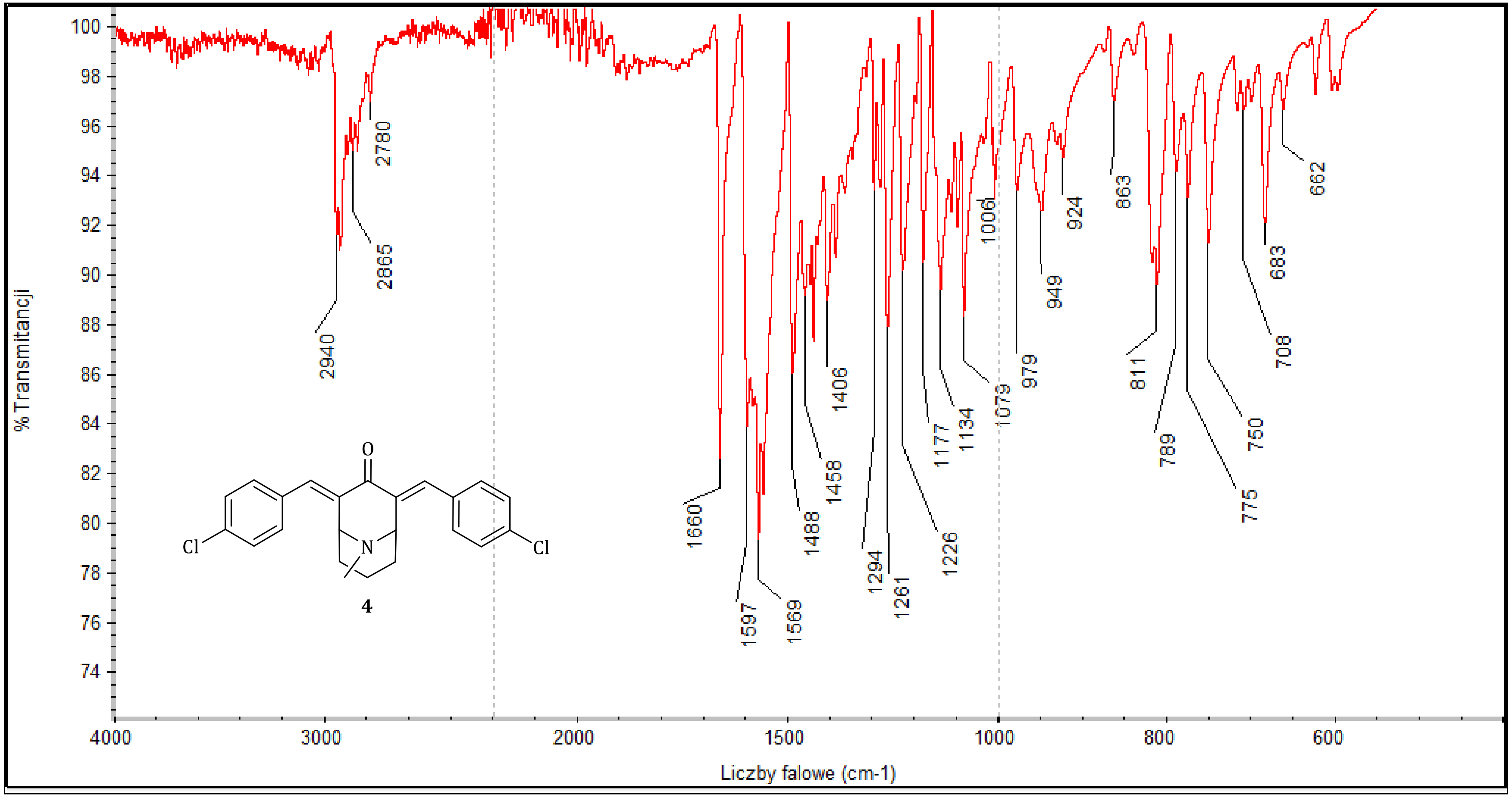

Figure S12. FTIR (ATR): 2,4-Bis((*E*)-4-chlorobenzylidene)-9-methyl-9-azabicyclo[3.3.1]nonan-3-one (**4**)

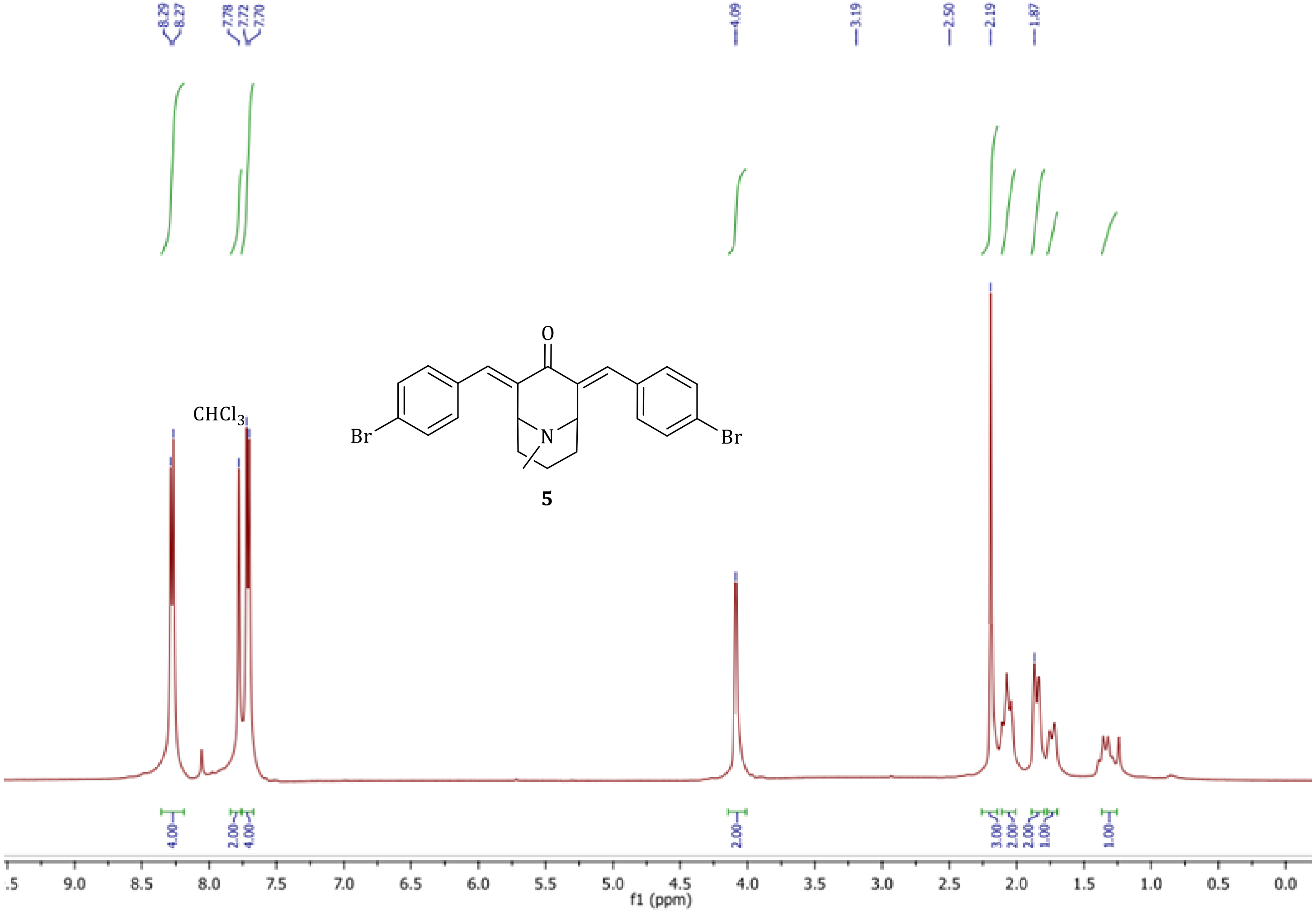

Figure S13. ^{1}H NMR (400 MHz, $CDCl_3$): 2,4-Bis((*E*)-4-bromobenzylidene)-9-methyl-9-azabicyclo[3.3.1]nonan-3-one (**5**)

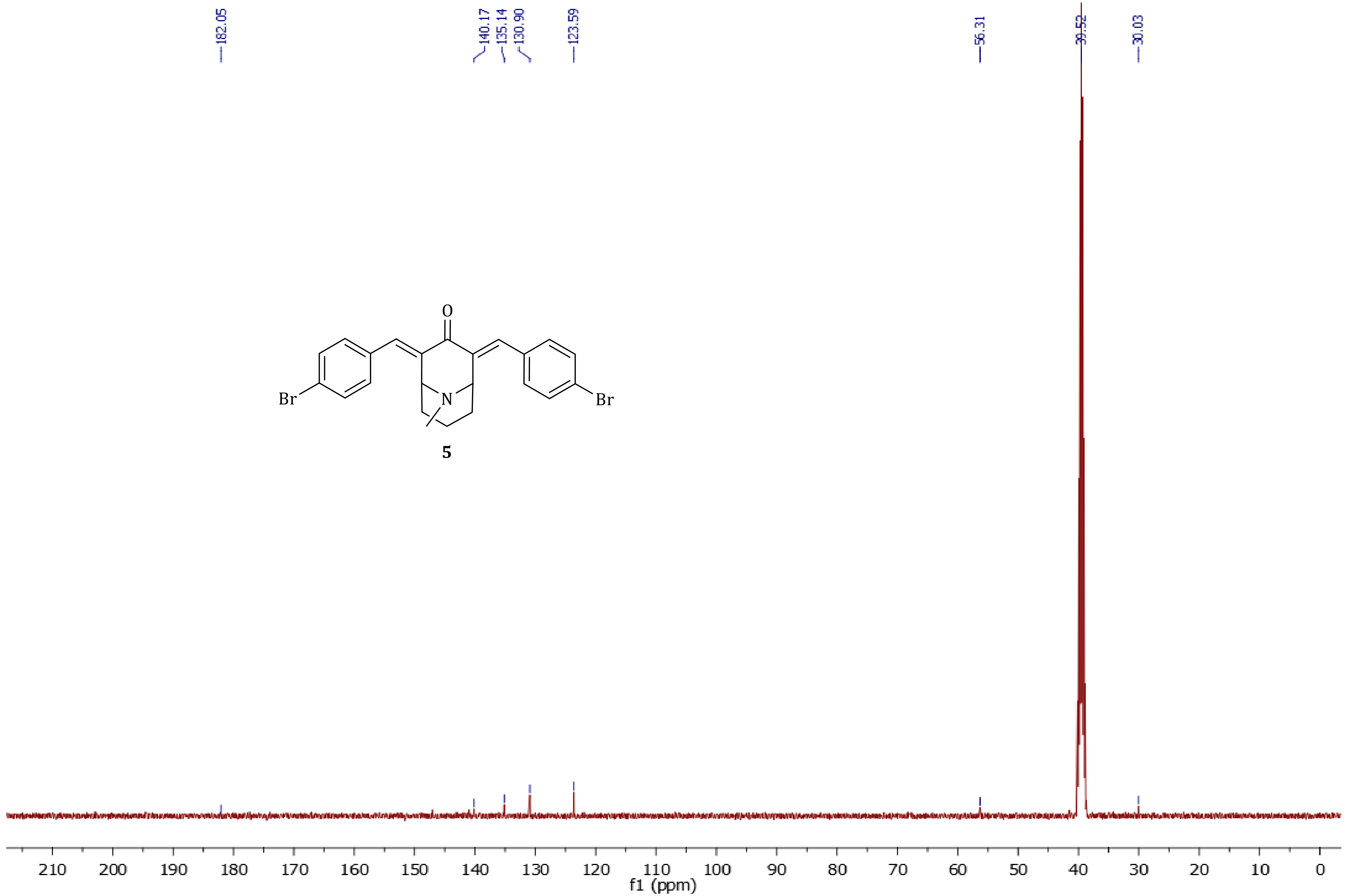

Figure S14. ^{13}C NMR (101 MHz, DMSO-d_6): 2,4-Bis((*E*)-4-bromobenzylidene)-9-methyl-9-azabicyclo[3.3.1]nonan-3-one (**5**)

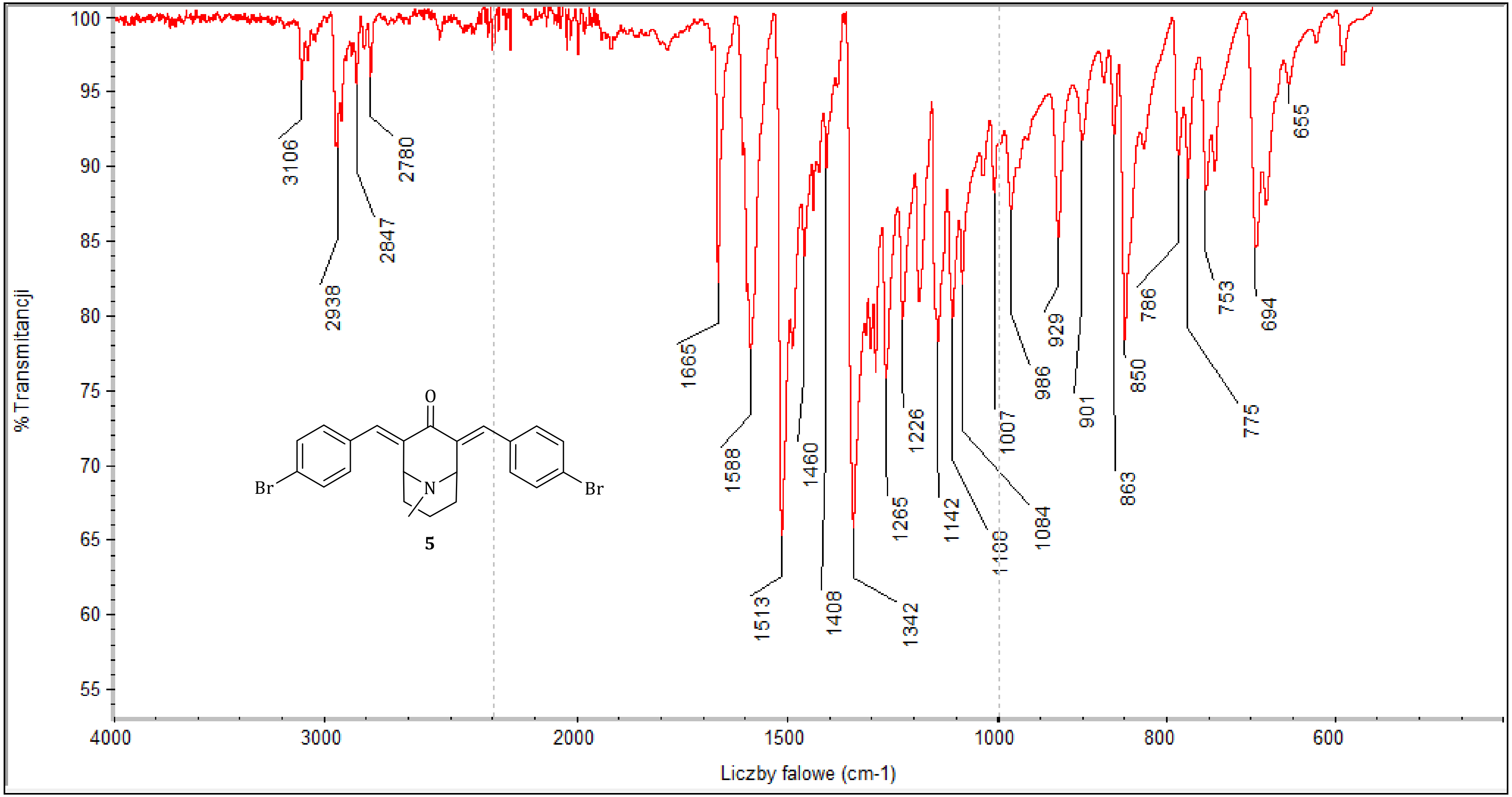

Figure S15. FTIR (ATR): 2,4-Bis((*E*)-4-bromobenzylidene)-9-methyl-9-azabicyclo[3.3.1]nonan-3-one (**5**)

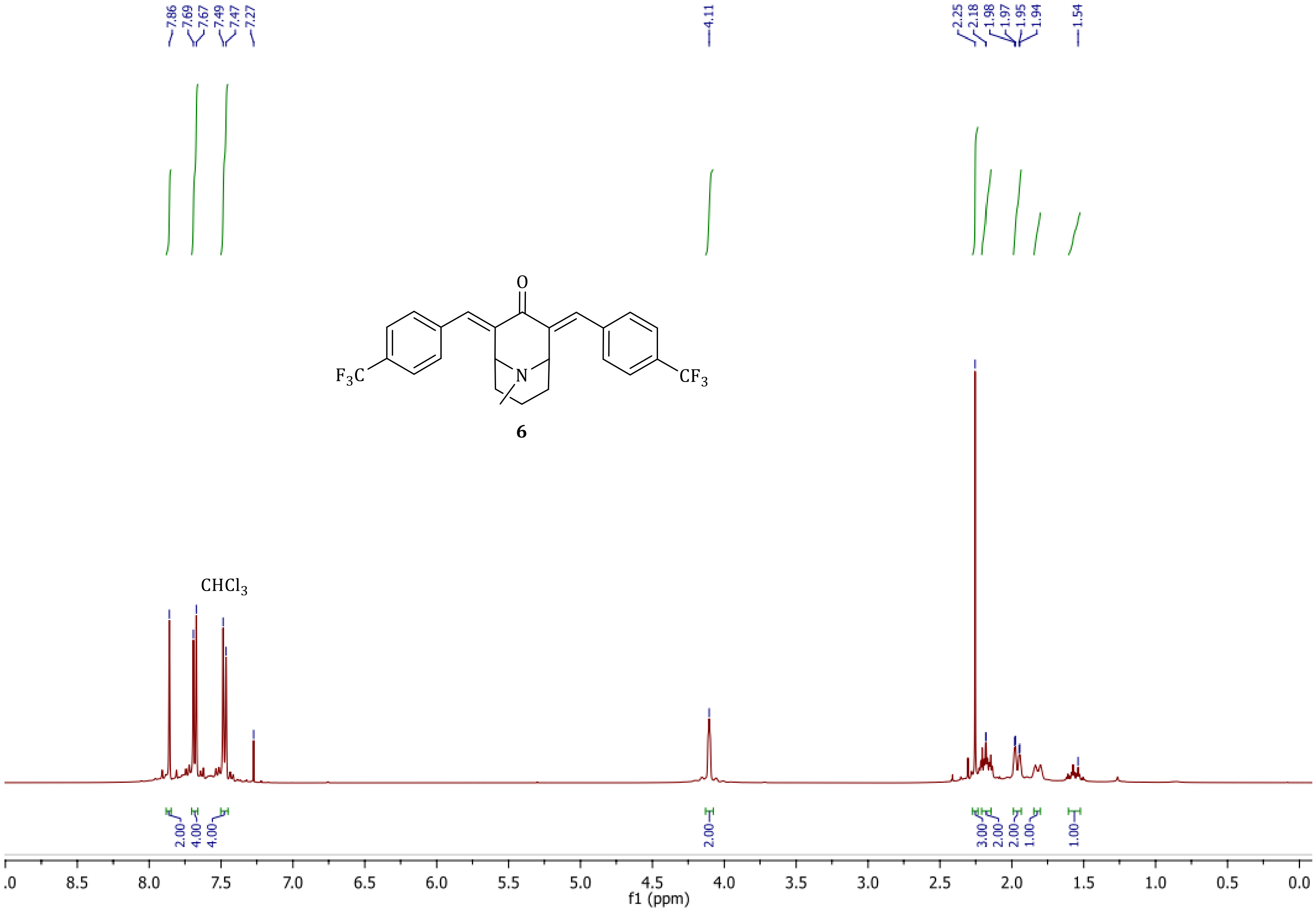

Figure S16. ^{1}H NMR (400 MHz, $CDCl_3$): 9-Methyl-2,4-bis((*E*)-4-(trifluoromethyl)benzylidene)-9-azabicyclo[3.3.1]nonan-3-one (**6**)

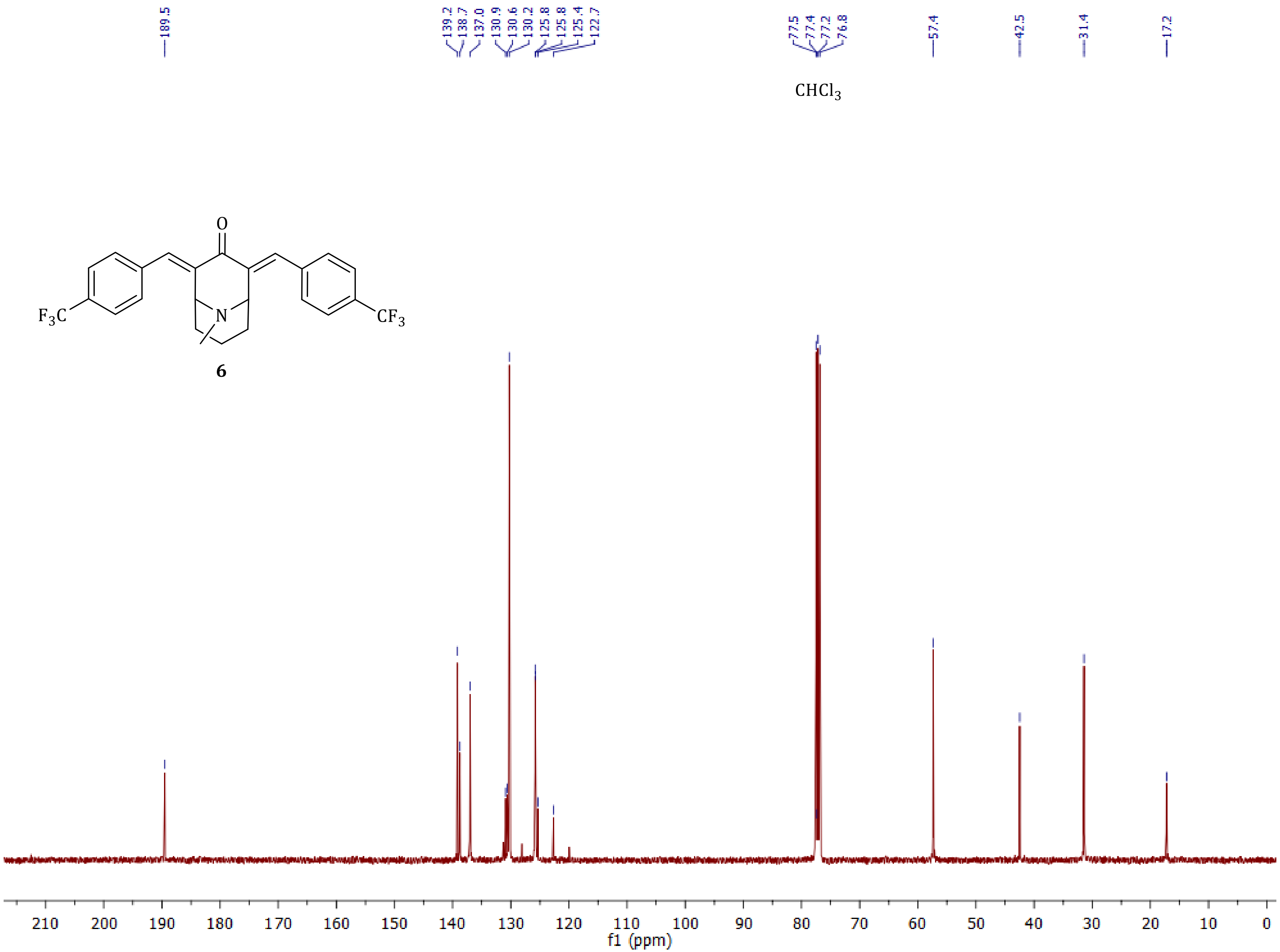

Figure S17. ^{13}C NMR (101 MHz, $CDCl_3$): 9-Methyl-2,4-bis((*E*)-4-(trifluoromethyl)benzylidene)-9-azabicyclo[3.3.1]nonan-3-one (**6**)

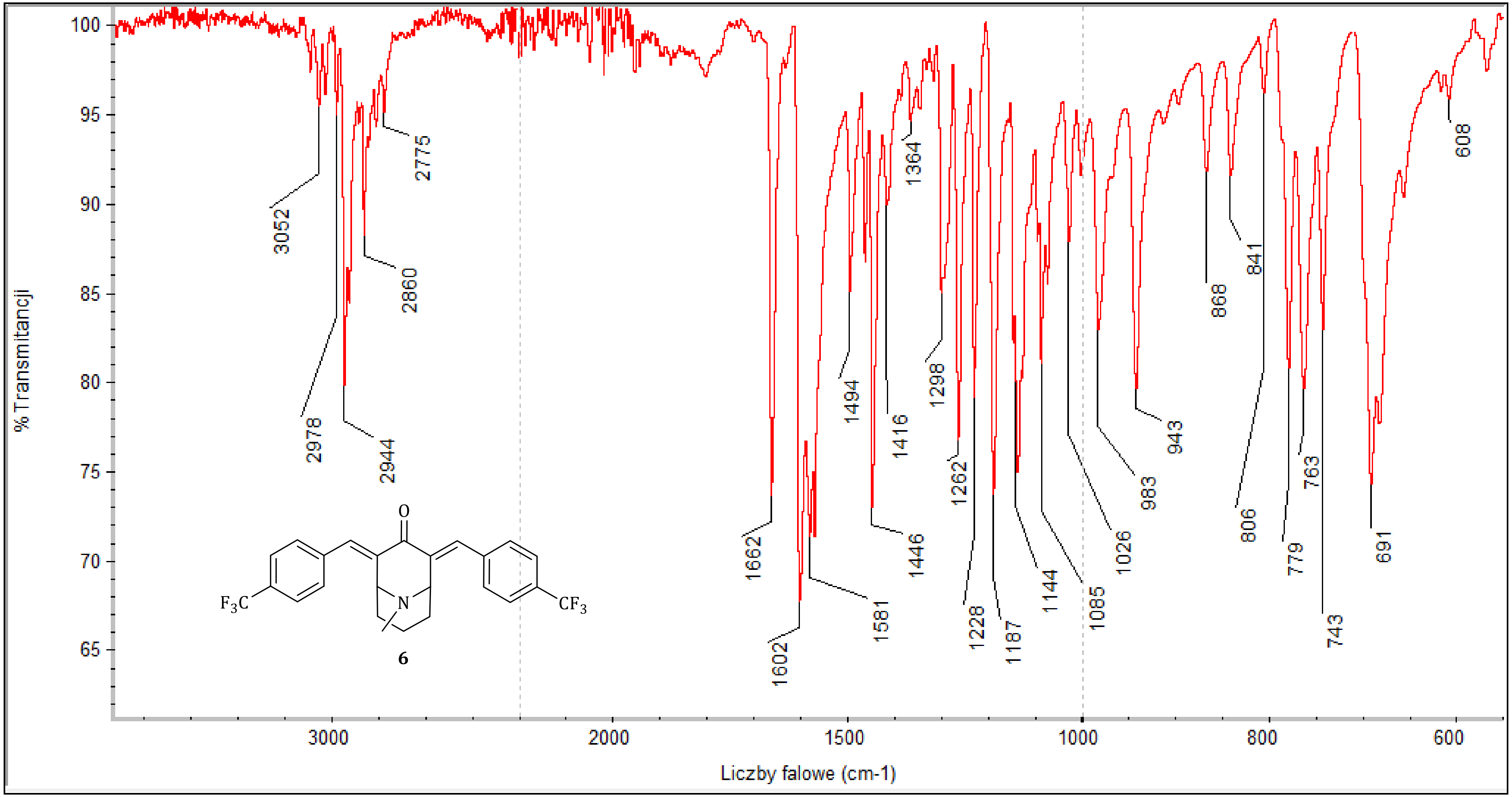

Figure S18. FTIR (ATR): 9-Methyl-2,4-bis((*E*)-4-(trifluoromethyl)benzylidene)-9-azabicyclo[3.3.1]nonan-3-one (**6**)

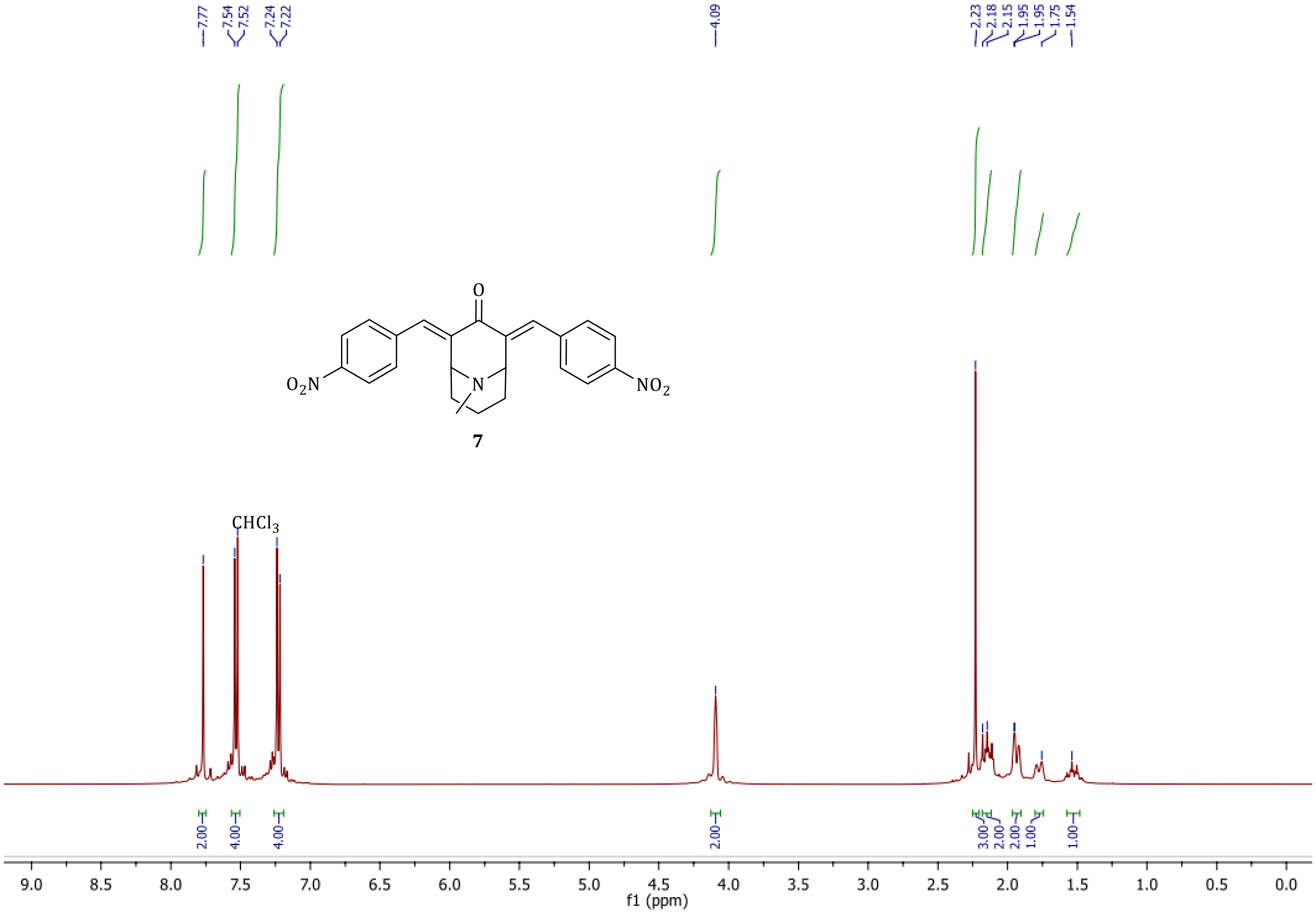

Figure S19. ^{1}H NMR (400 MHz, $CDCl_3$): 9-Methyl-2,4-bis((*E*)-4-nitrobenzylidene)-9-azabicyclo[3.3.1]nonan-3-one (**7**)

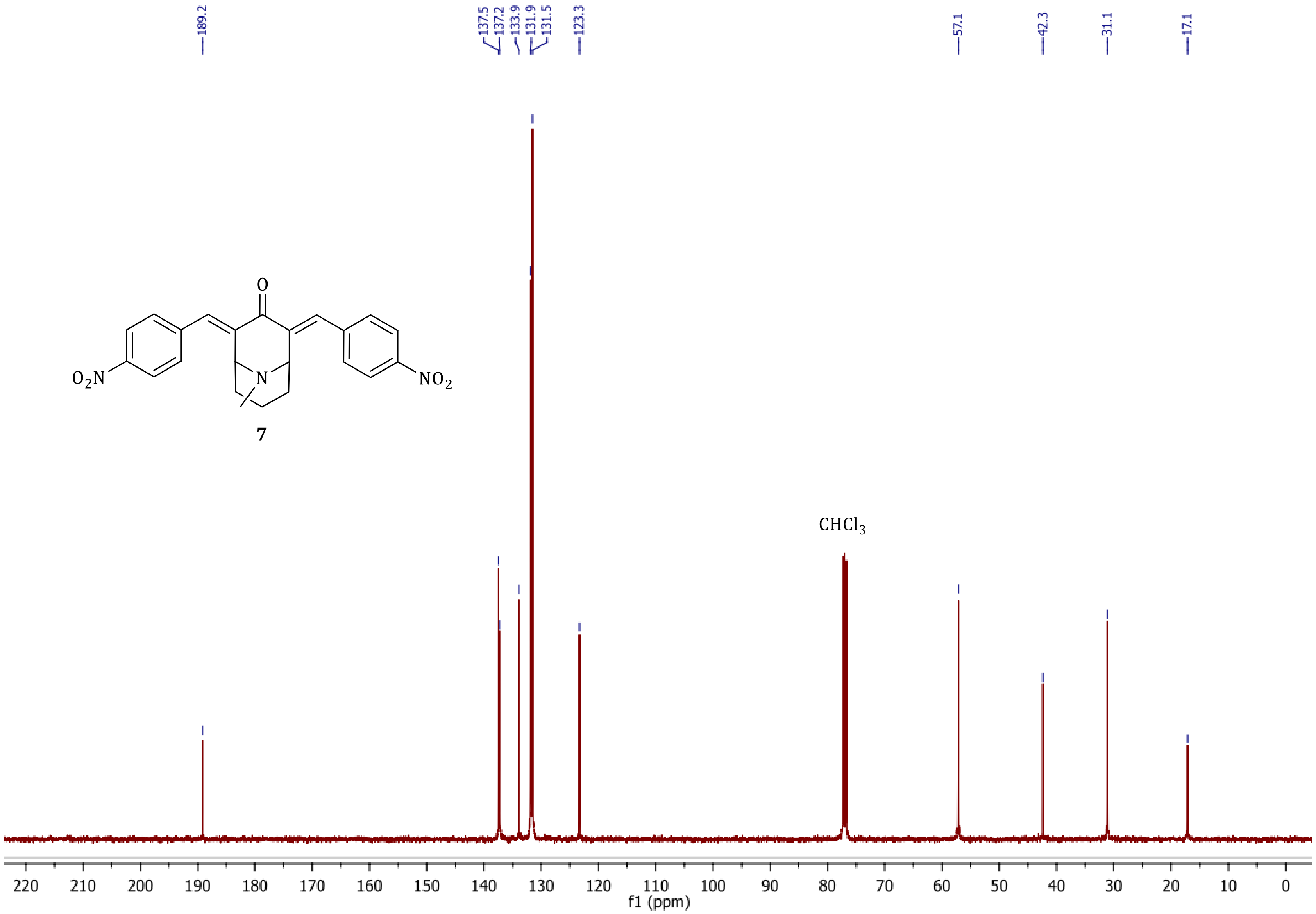

Figure S20. ^{13}C NMR (101 MHz, $CDCl_3$): 9-Methyl-2,4-bis((*E*)-4-nitrobenzylidene)-9-azabicyclo[3.3.1]nonan-3-one (**7**)

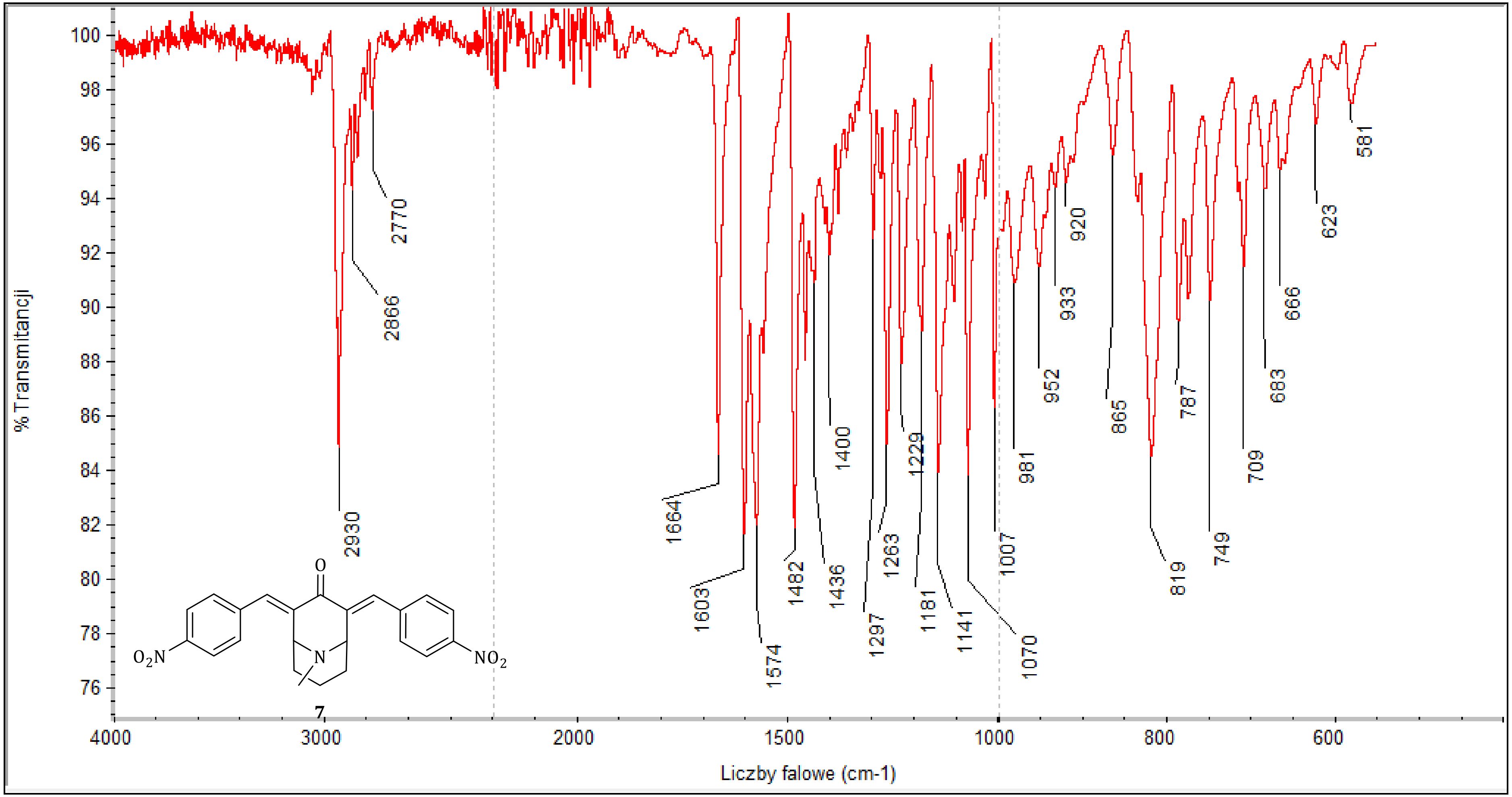

Figure S21. FTIR (ATR): 9-Methyl-2,4-bis((*E*)-4-nitrobenzylidene)-9-azabicyclo[3.3.1]nonan-3-one (**7**)

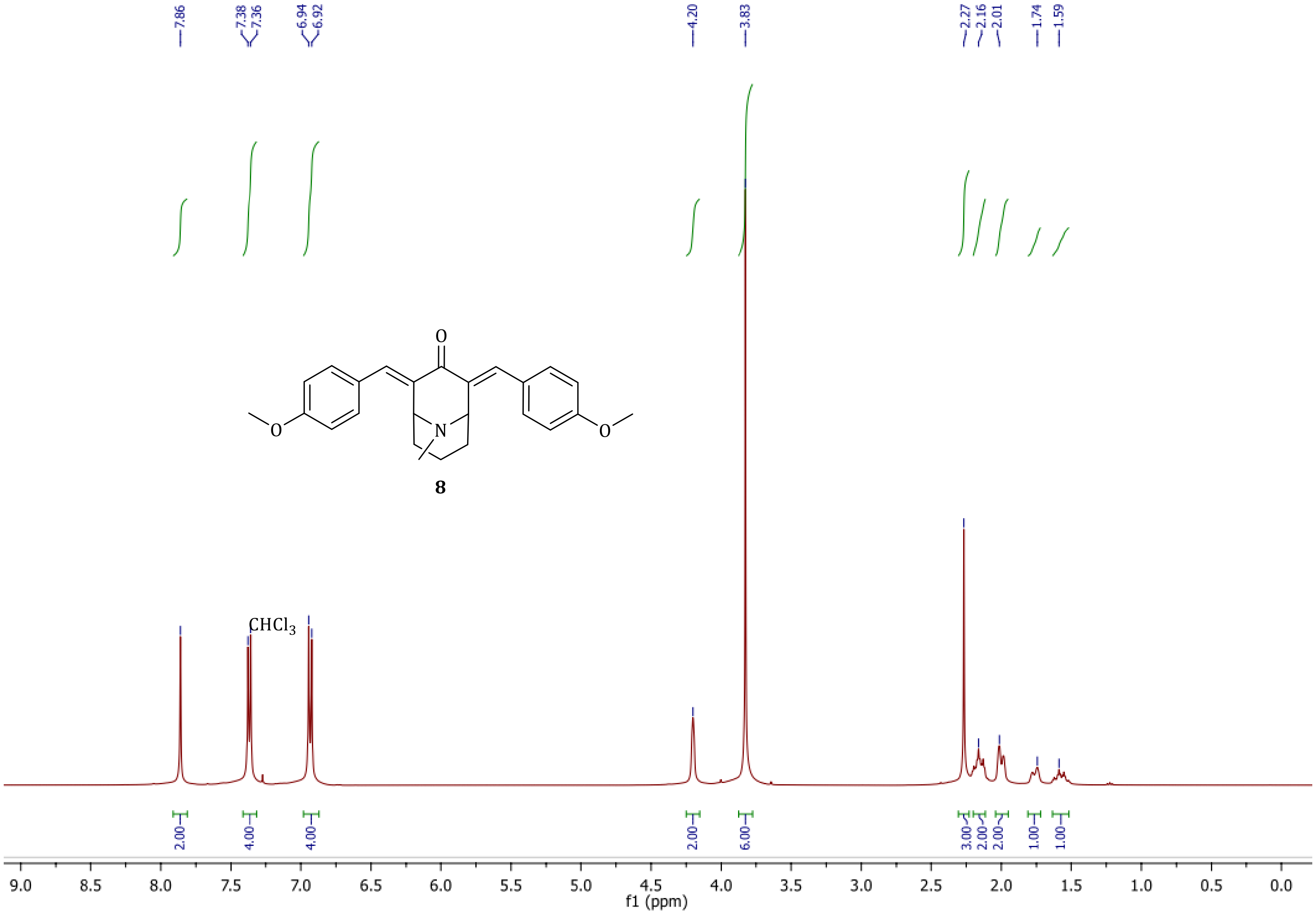

Figure S22. ^{1}H NMR (400 MHz, $CDCl_3$): 4-Bis((*E*)-4-methoxybenzylidene)-9-methyl-9-azabicyclo[3.3.1]nonan-3-one (**8**)

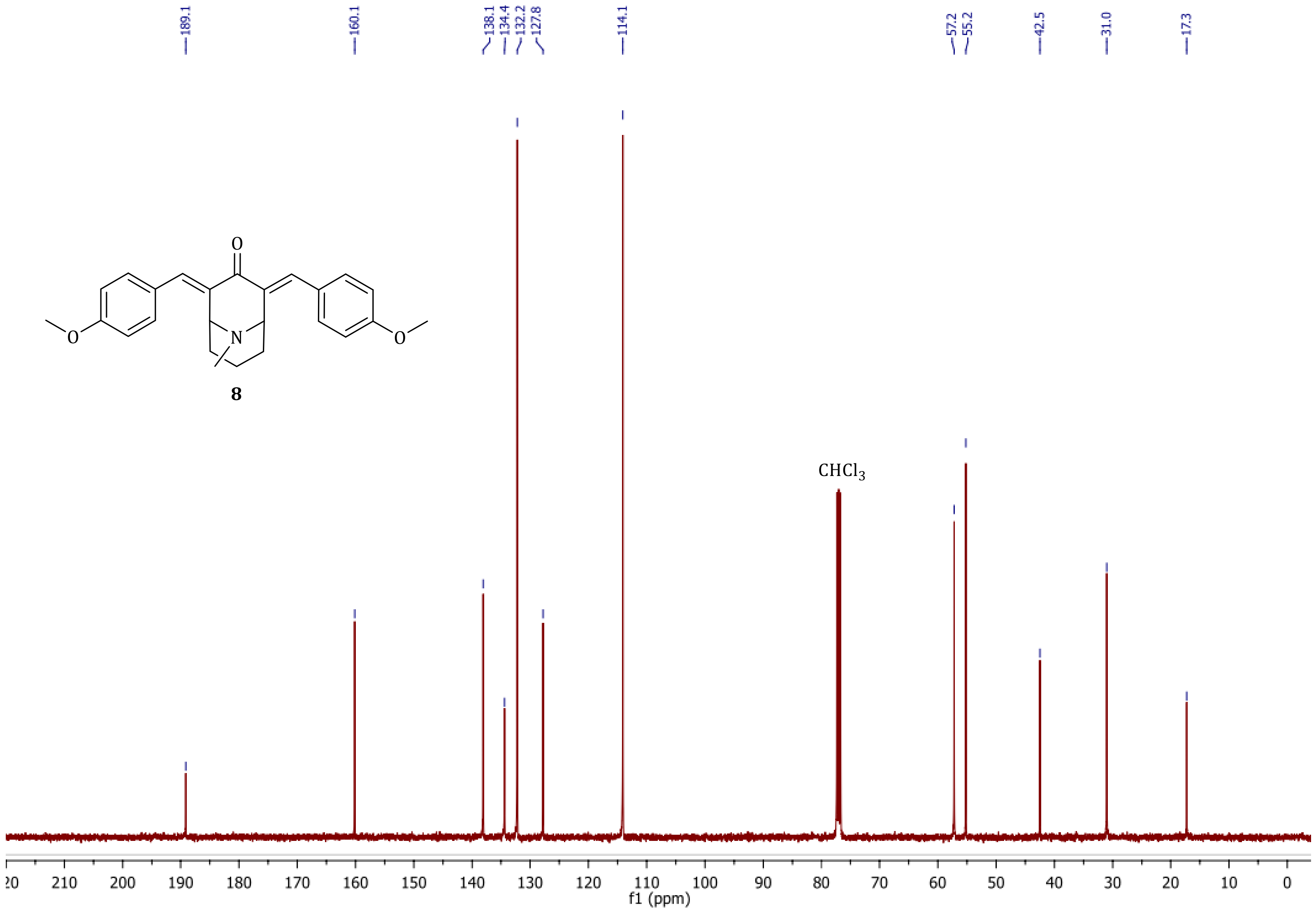

Figure S23. ^{13}C NMR (101 MHz, $CDCl_3$): 4-Bis((*E*)-4-methoxybenzylidene)-9-methyl-9-azabicyclo[3.3.1]nonan-3-one (**8**)

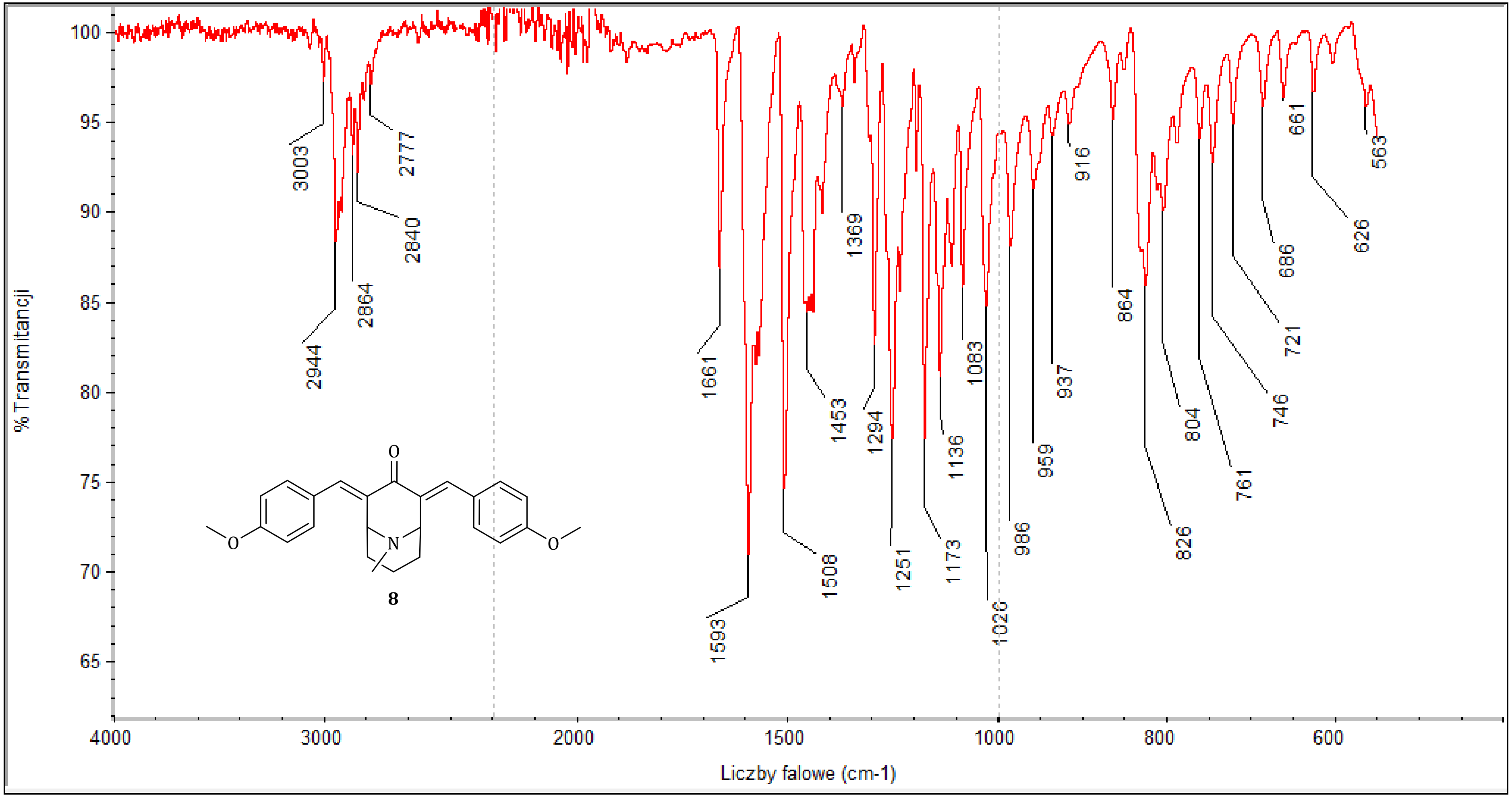

Figure S24. FTIR (ATR): 4-Bis((*E*)-4-methoxybenzylidene)-9-methyl-9-azabicyclo[3.3.1]nonan-3-one (**8**)

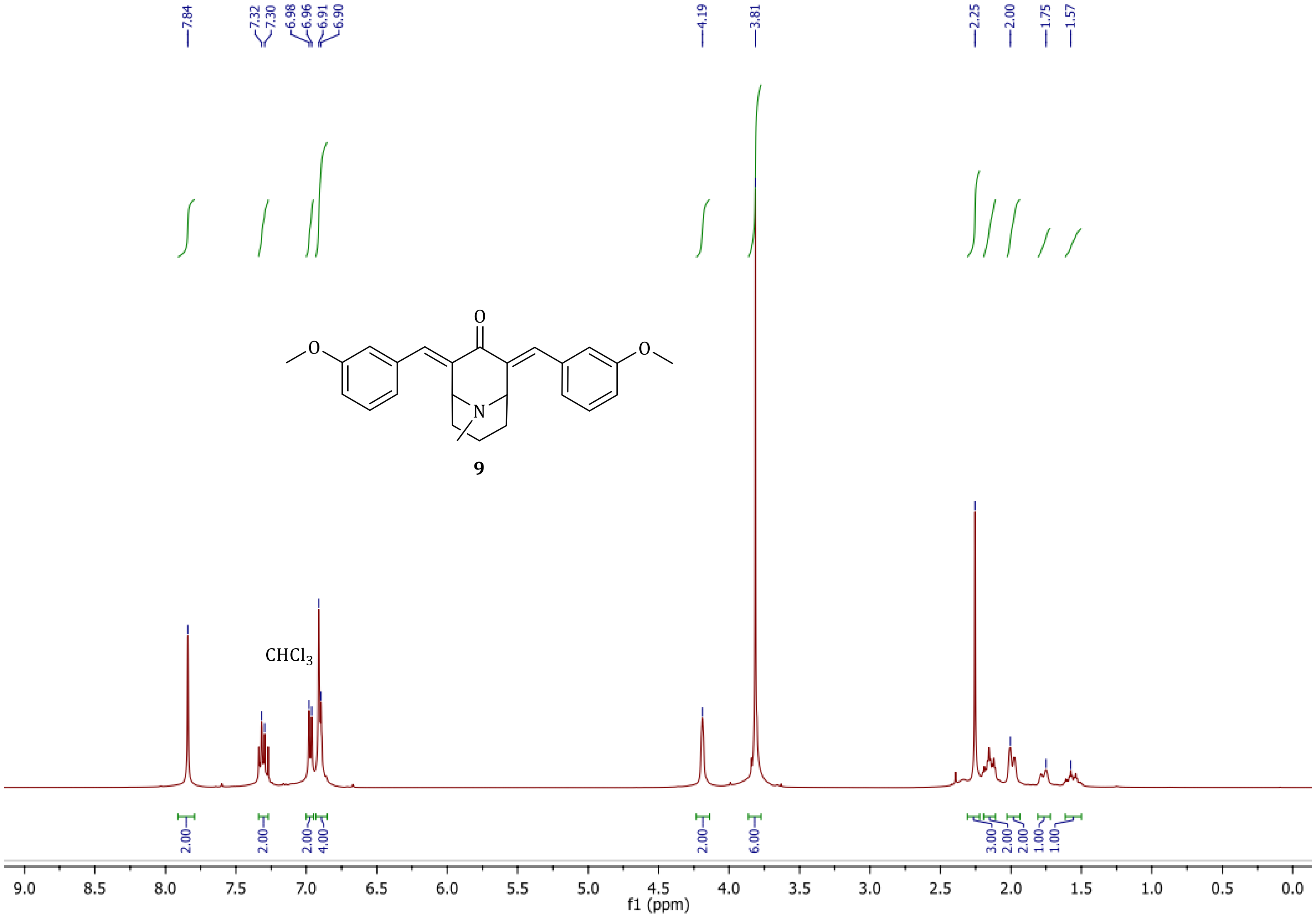

Figure S25. ^{1}H NMR (400 MHz, $CDCl_3$): 4-Bis((*E*)-3-methoxybenzylidene)-9-methyl-9-azabicyclo[3.3.1]nonan-3-one (**9**)

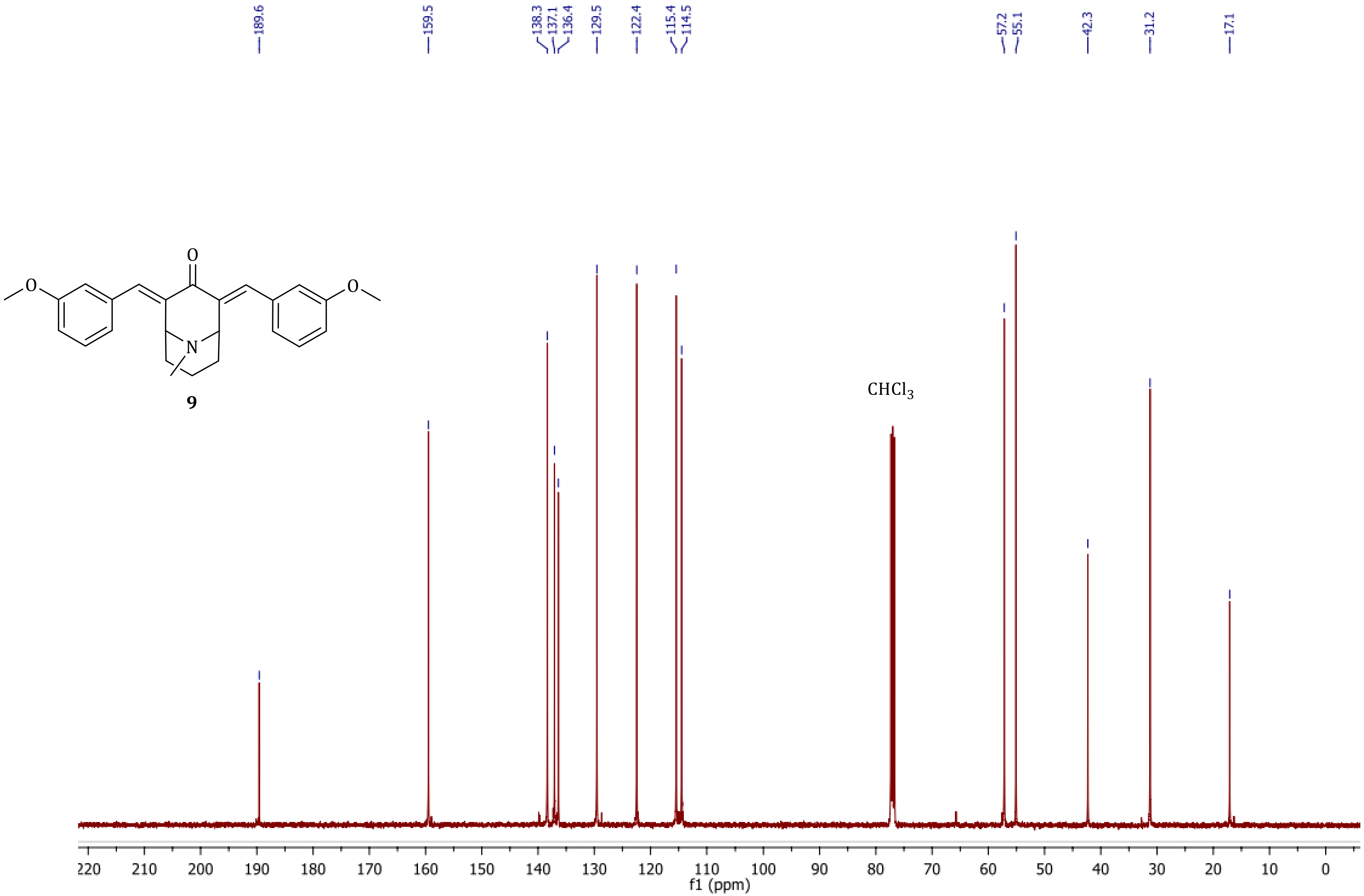

Figure S26. ^{13}C NMR (101 MHz, $CDCl_3$): 4-Bis((*E*)-3-methoxybenzylidene)-9-methyl-9-azabicyclo[3.3.1]nonan-3-one (**9**)

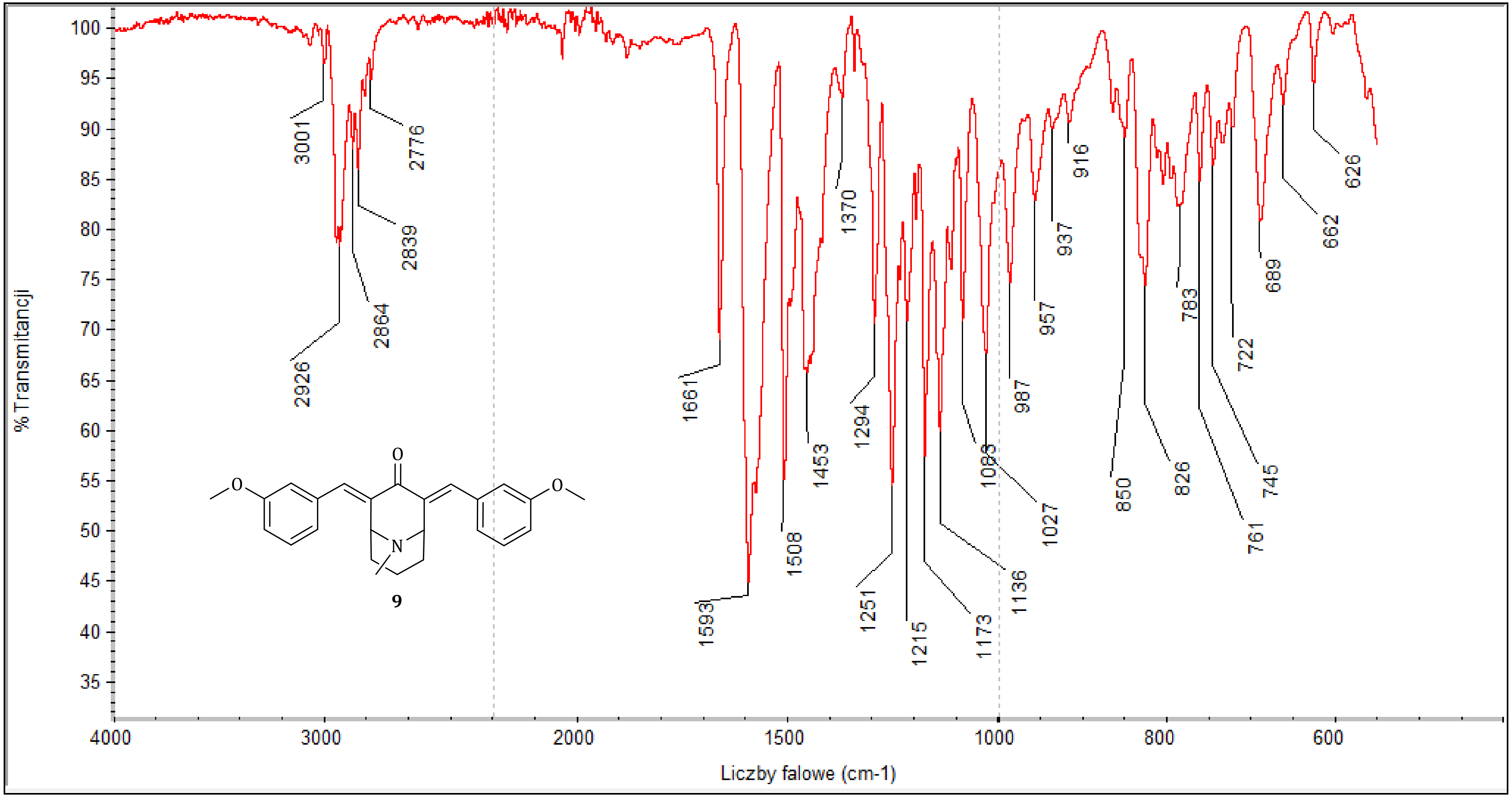

Figure S27. FTIR (ATR): 4-Bis((*E*)-3-methoxybenzylidene)-9-methyl-9-azabicyclo[3.3.1]nonan-3-one (**9**)

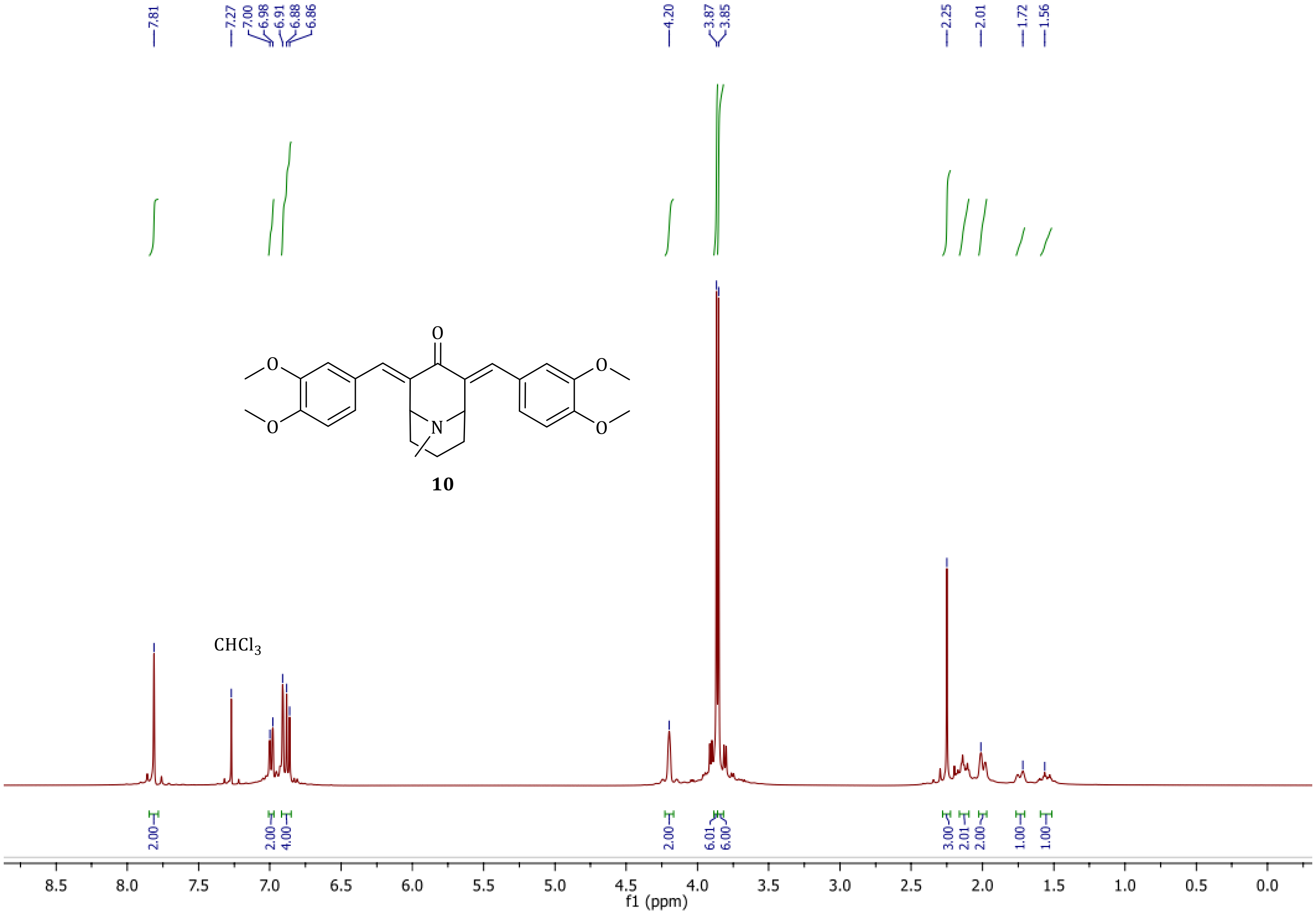

Figure S28. ^{1}H NMR (400 MHz, $CDCl_3$): 2,4-Bis((*E*)-3,4-dimethoxybenzylidene)-9-methyl-9-azabicyclo[3.3.1]nonan-3-one (**10**)

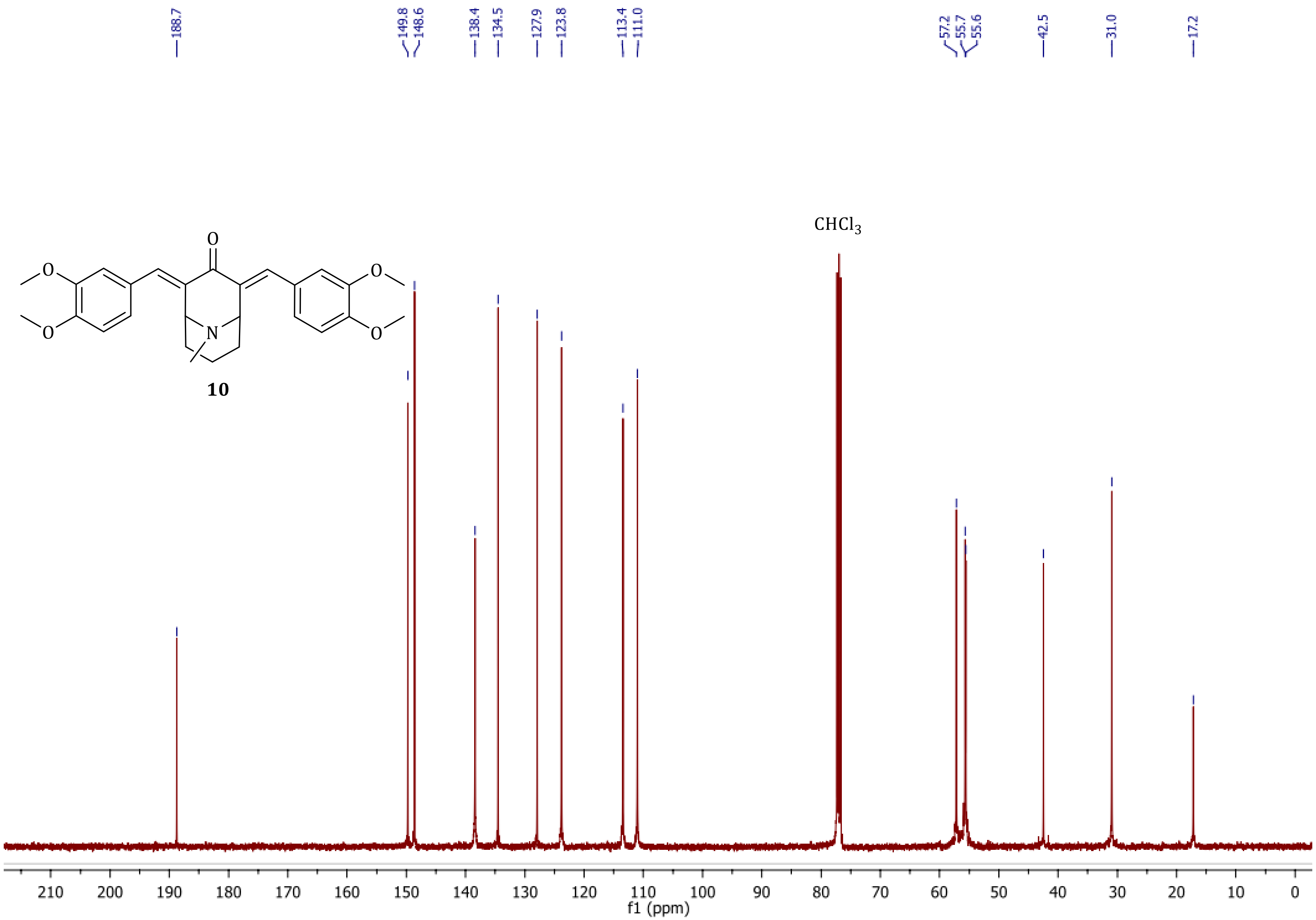

Figure S29. ^{13}C NMR (101 MHz, $CDCl_3$): 2,4-bis((*E*)-3,4-dimethoxybenzylidene)-9-methyl-9-azabicyclo[3.3.1]nonan-3-one (**10**)

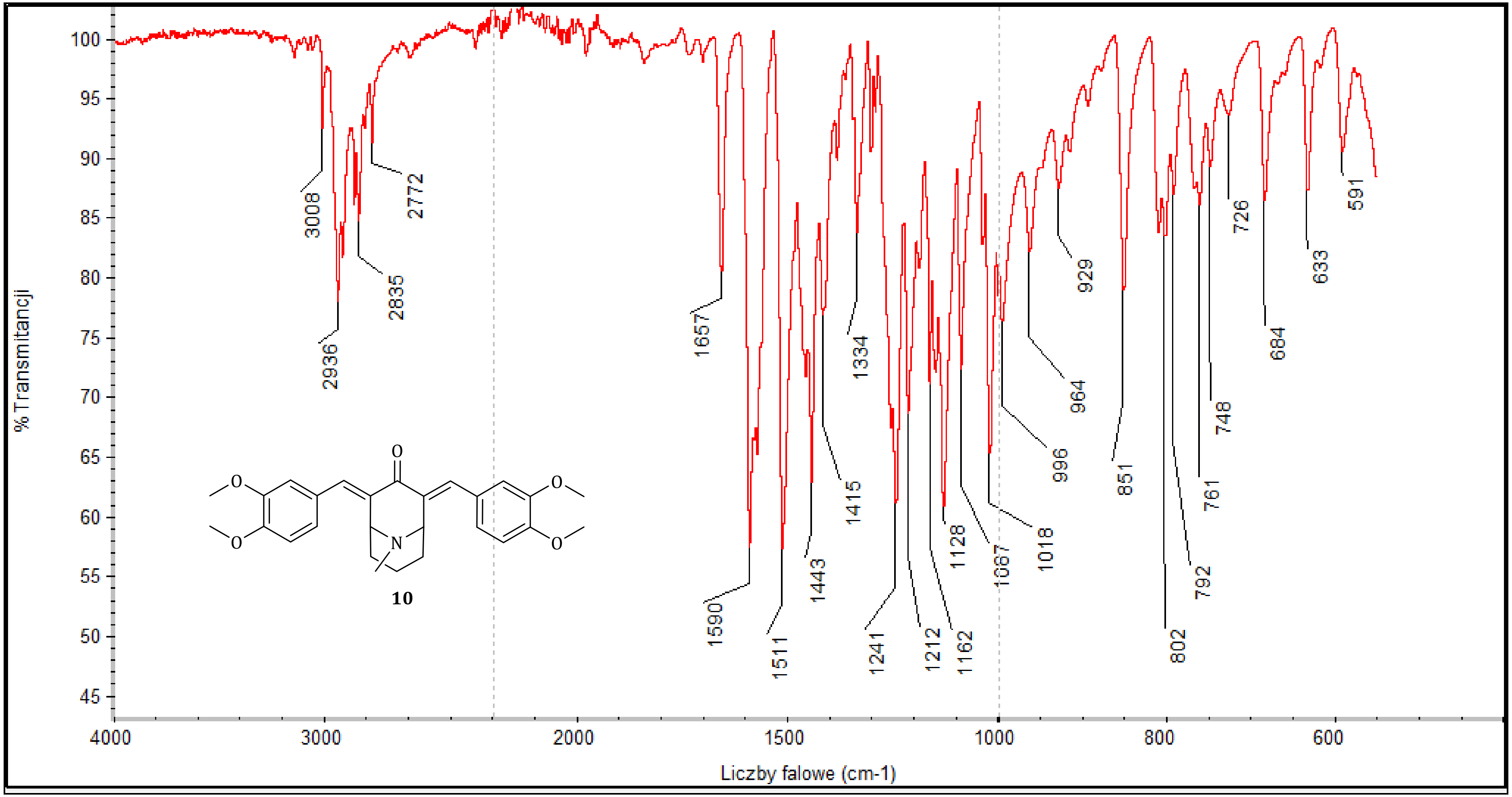

Figure S30. FTIR (ATR): 2,4-bis((*E*)-3,4-dimethoxybenzylidene)-9-methyl-9-azabicyclo[3.3.1]nonan-3-one (**10**)

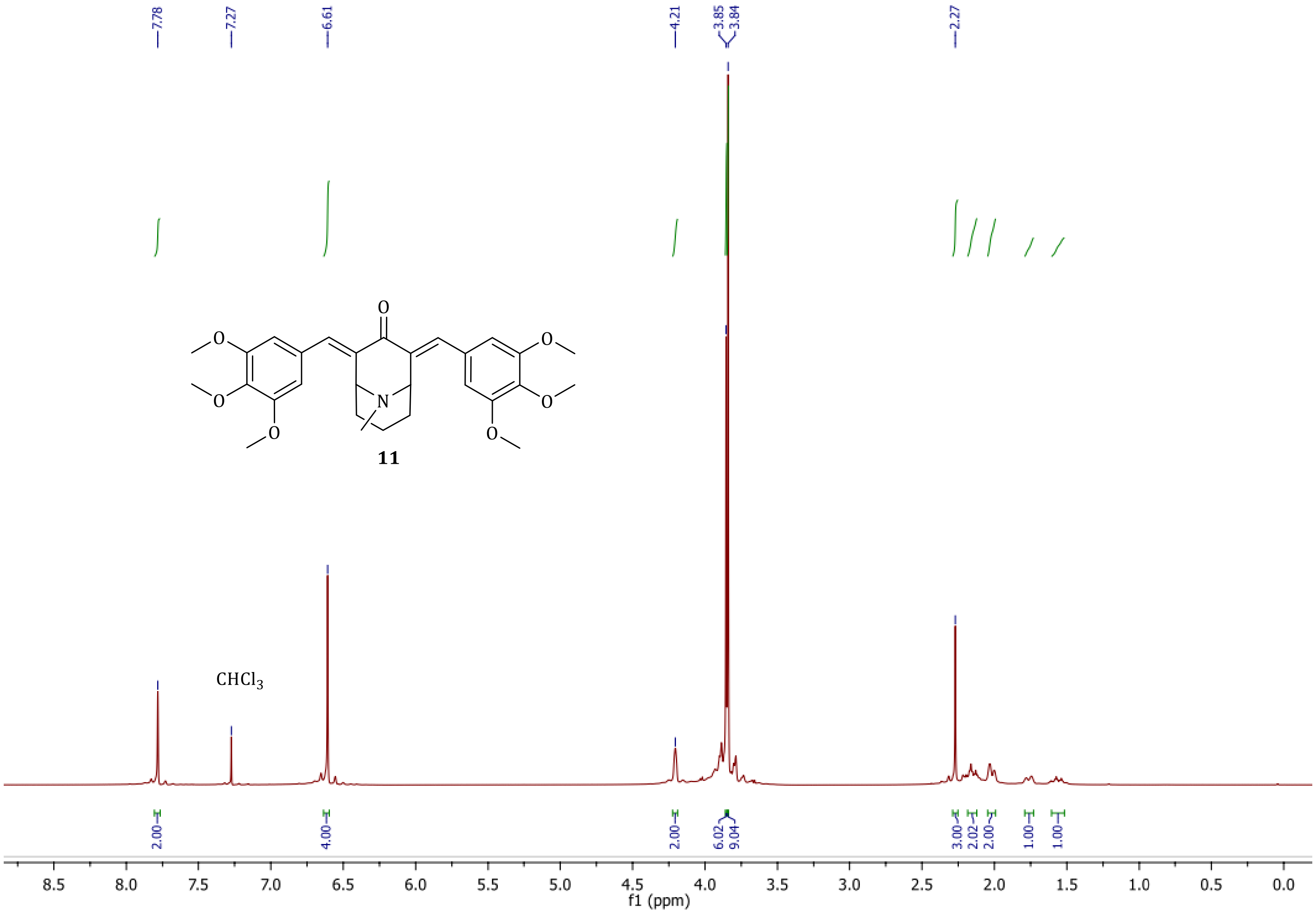

Figure S31. ^{1}H NMR (400 MHz, $CDCl_3$): 9-Methyl-2,4-bis((*E*)-3,4,5-trimethoxybenzylidene)-9-azabicyclo[3.3.1]nonan-3-one (**11**)

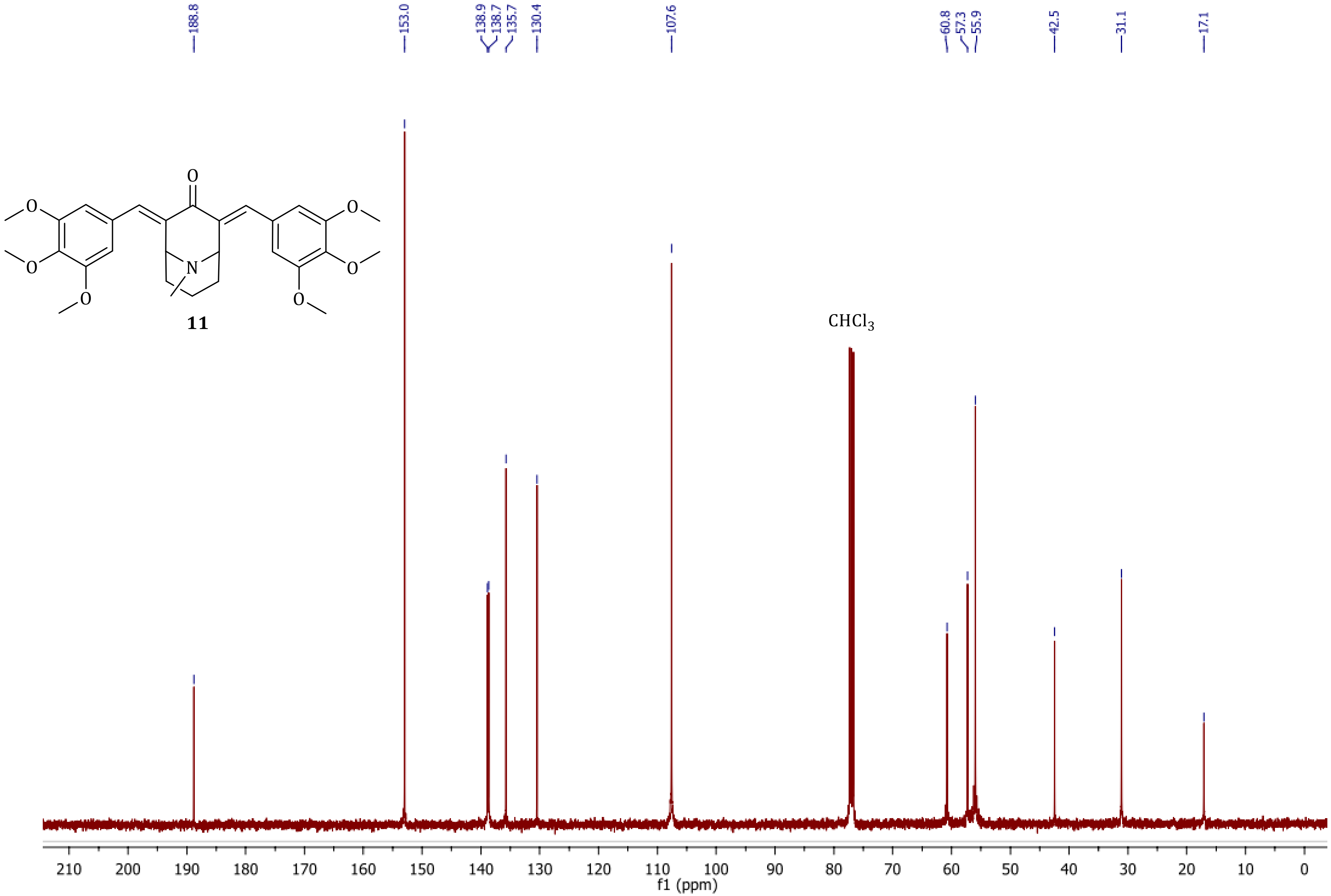

Figure S32. ^{13}C NMR (101 MHz, $CDCl_3$): 9-Methyl-2,4-bis((*E*)-3,4,5-trimethoxybenzylidene)-9-azabicyclo[3.3.1]nonan-3-one (**11**)

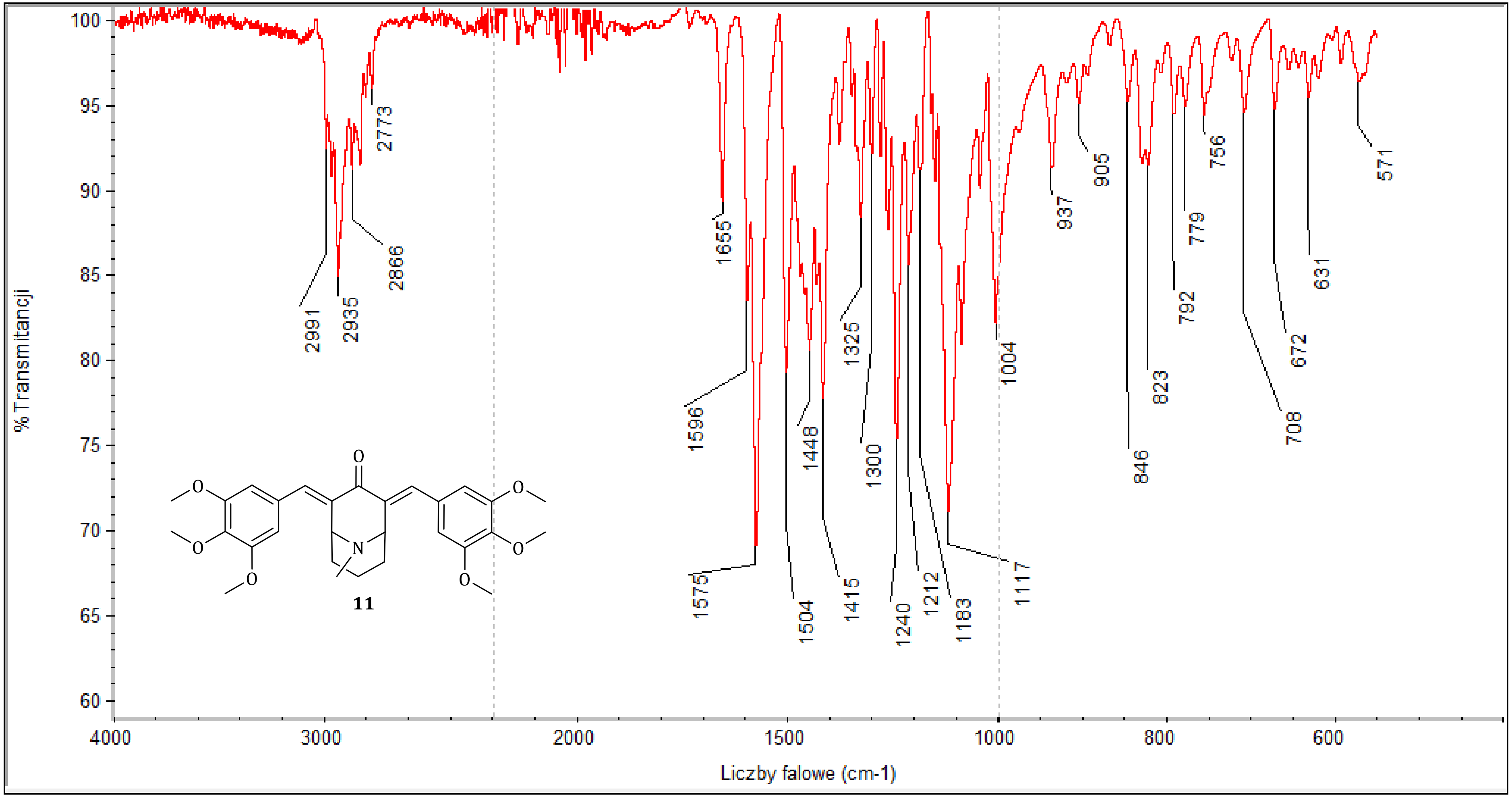

Figure S33. FTIR (ATR): 9-Methyl-2,4-bis((*E*)-3,4,5-trimethoxybenzylidene)-9-azabicyclo[3.3.1]nonan-3-one (**11**)

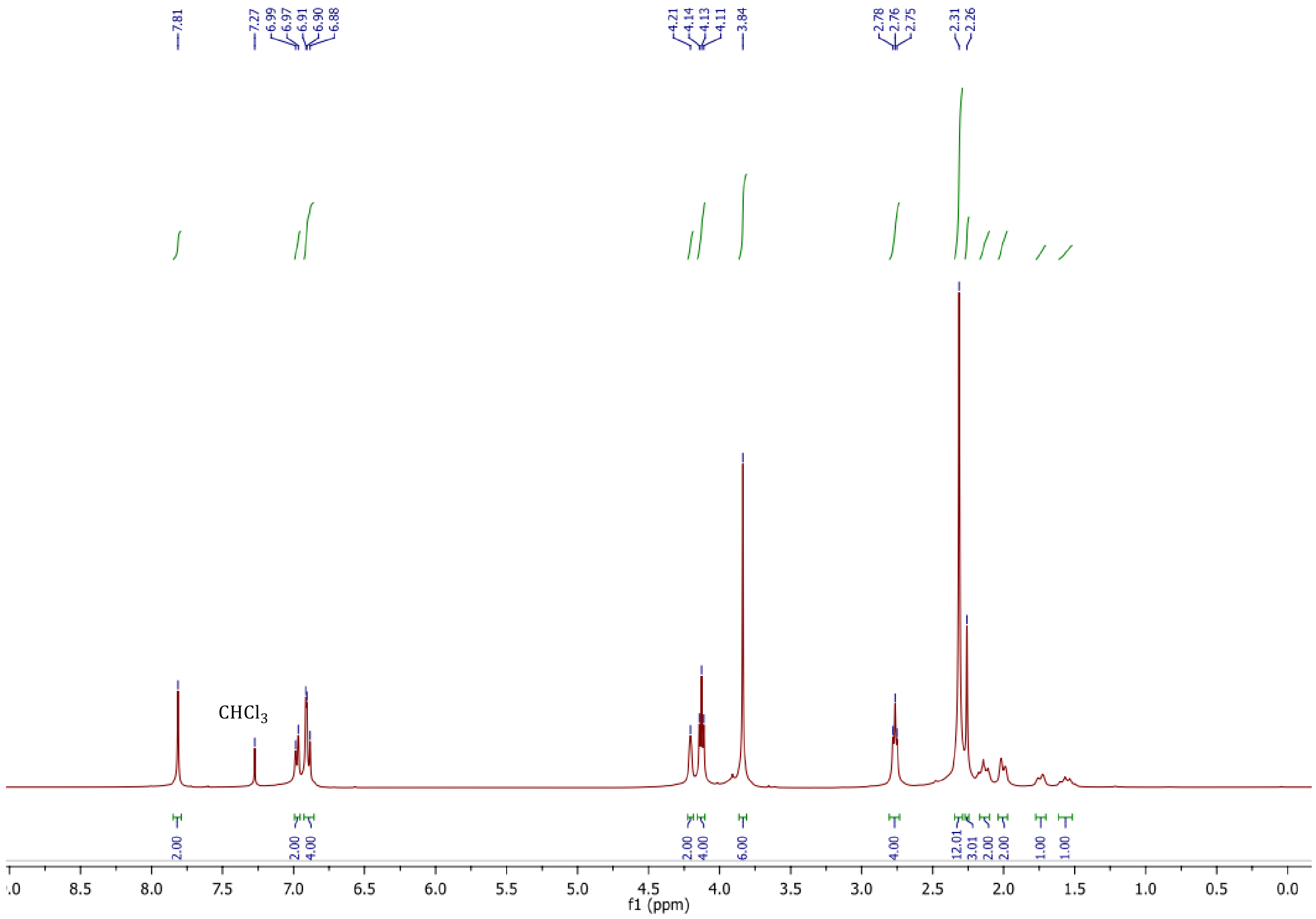

Figure S34. ^{1}H NMR (400 MHz, $CDCl_3$): 2,4-Bis((*E*)-4-(2-(dimethylamino)ethoxy)-3-methoxybenzylidene)-9-methyl-9-azabicyclo[3.3.1]nonan-3-one (**12**)

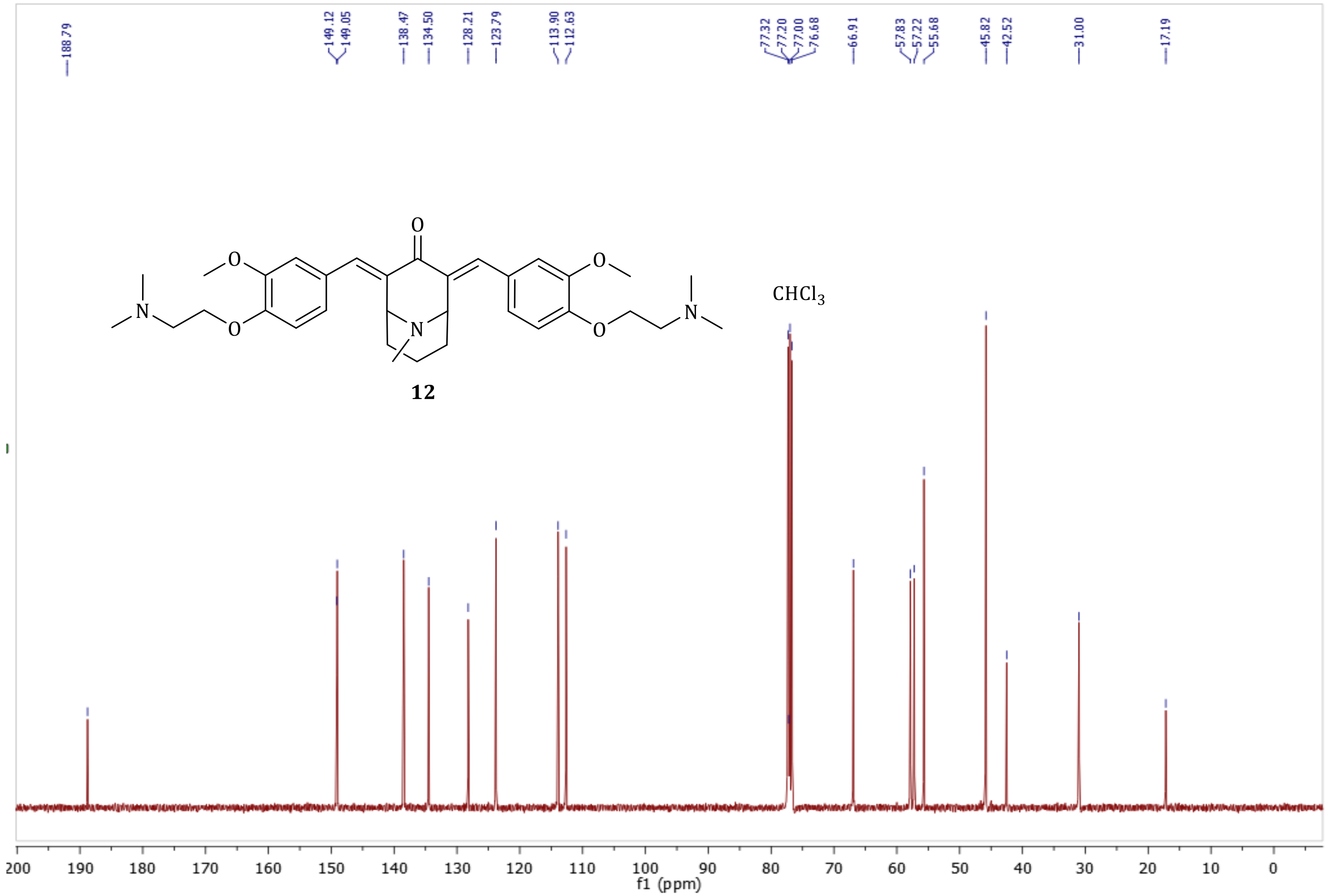

Figure S35. ^{13}C NMR (101 MHz, $CDCl_3$): 2,4-Bis((*E*)-4-(2-(dimethylamino)ethoxy)-3-methoxybenzylidene)-9-methyl-9-azabicyclo[3.3.1]nonan-3-one (**12**)

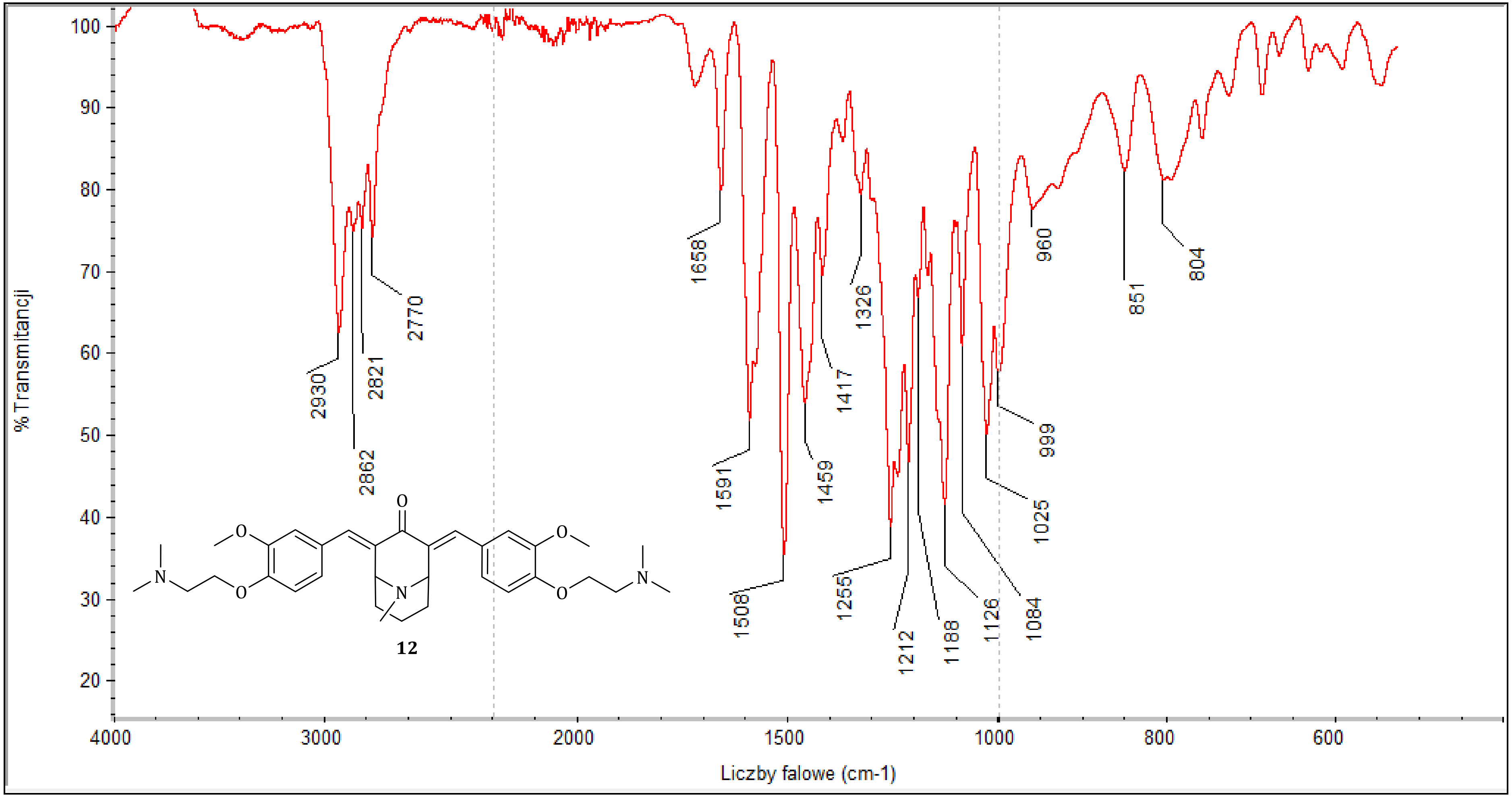

Figure S36. FTIR (ATR): 2,4-Bis((*E*)-4-(2-(dimethylamino)ethoxy)-3-methoxybenzylidene)-9-methyl-9-azabicyclo[3.3.1]nonan-3-one (**12**)

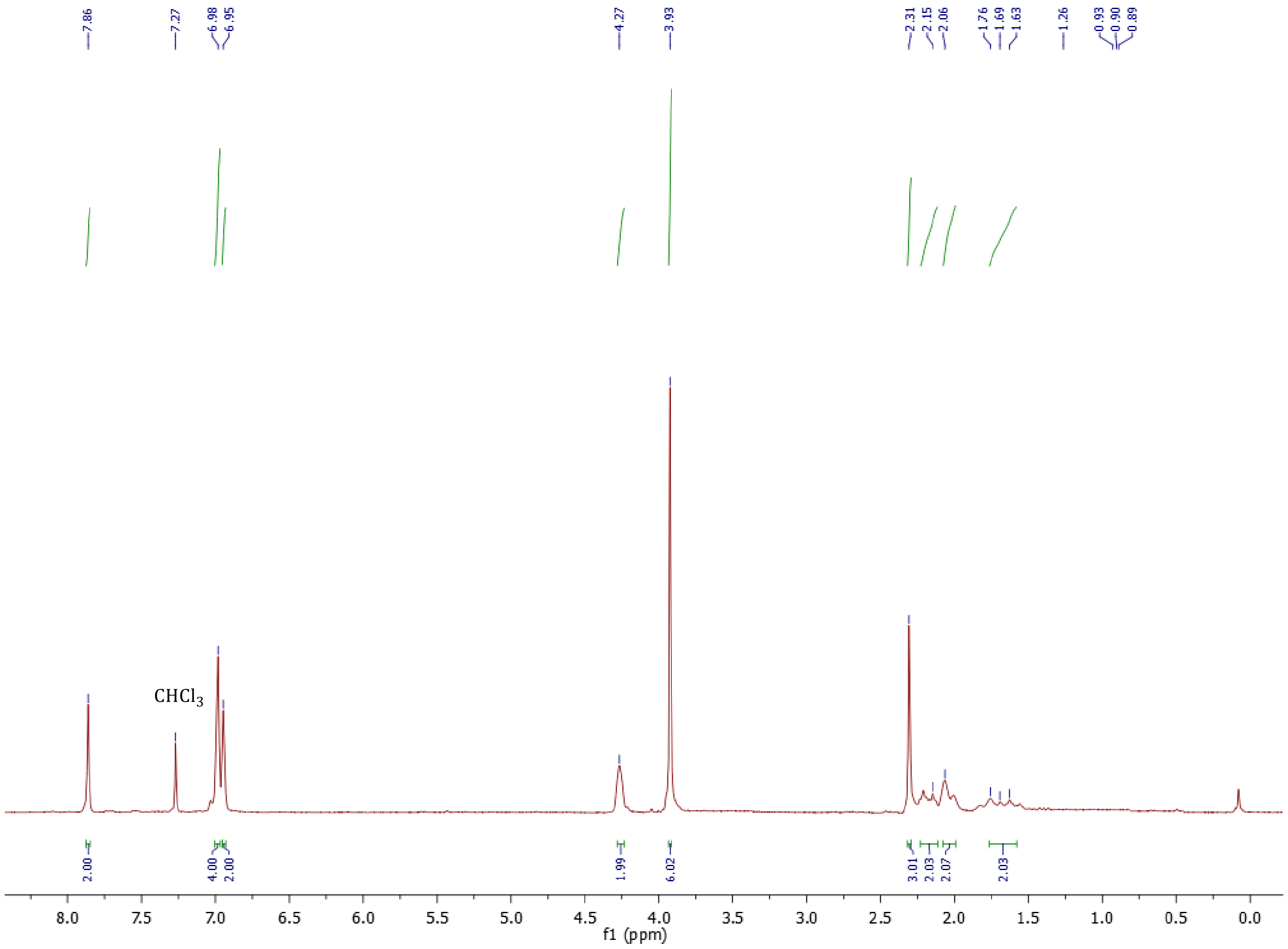

Figure S37. ^{1}H NMR (200 MHz, $CDCl_3$): 2,4-Bis((*E*)-4-hydroxy-3-methoxybenzylidene)-9-methyl-9-azabicyclo[3.3.1]nonan-3-one (**13**)

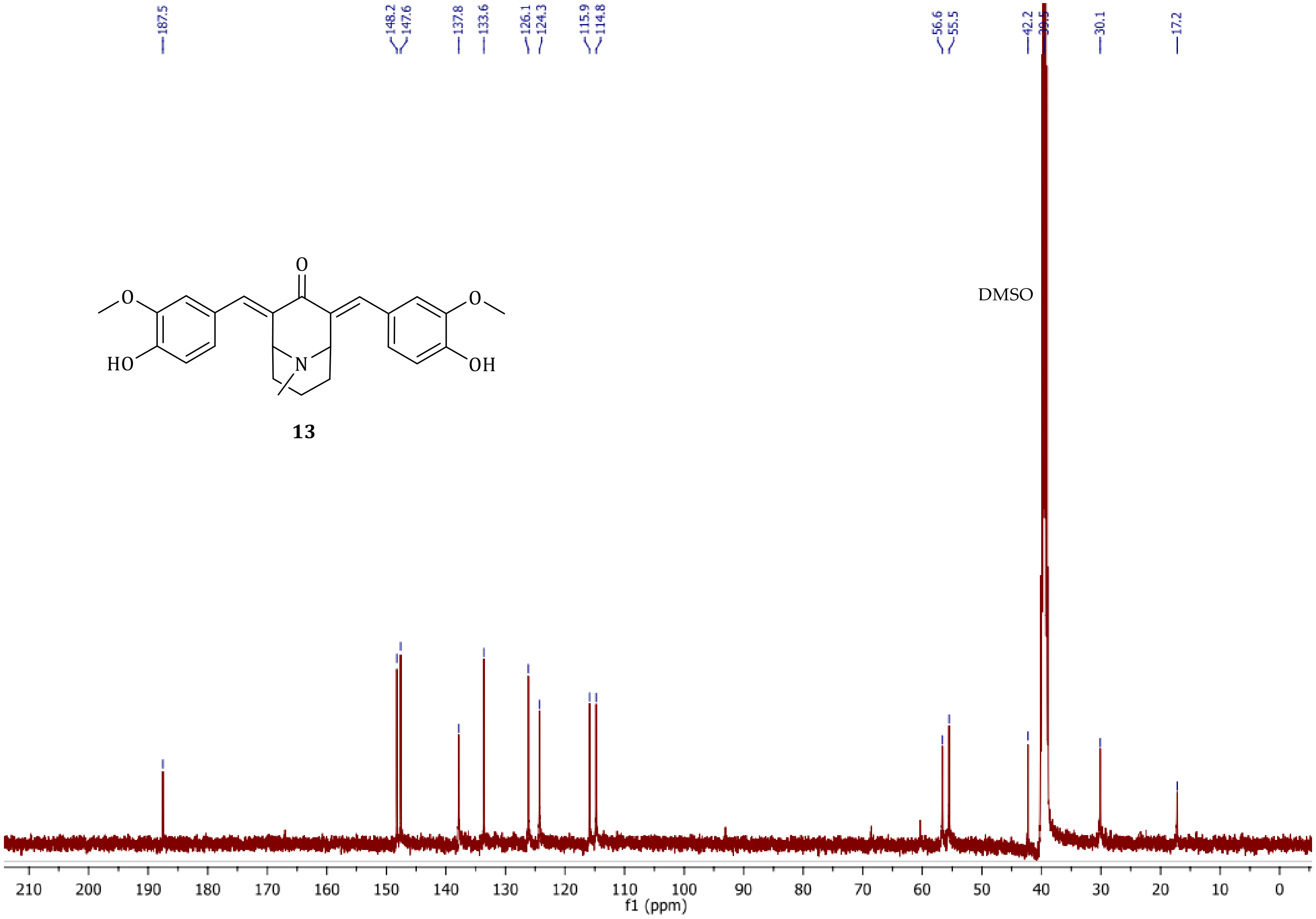

Figure S38. ^{13}C NMR (101 MHz, DMSO-d_6): 2,4-Bis((*E*)-4-hydroxy-3-methoxybenzylidene)-9-methyl-9-azabicyclo[3.3.1]nonan-3-one (**13**)

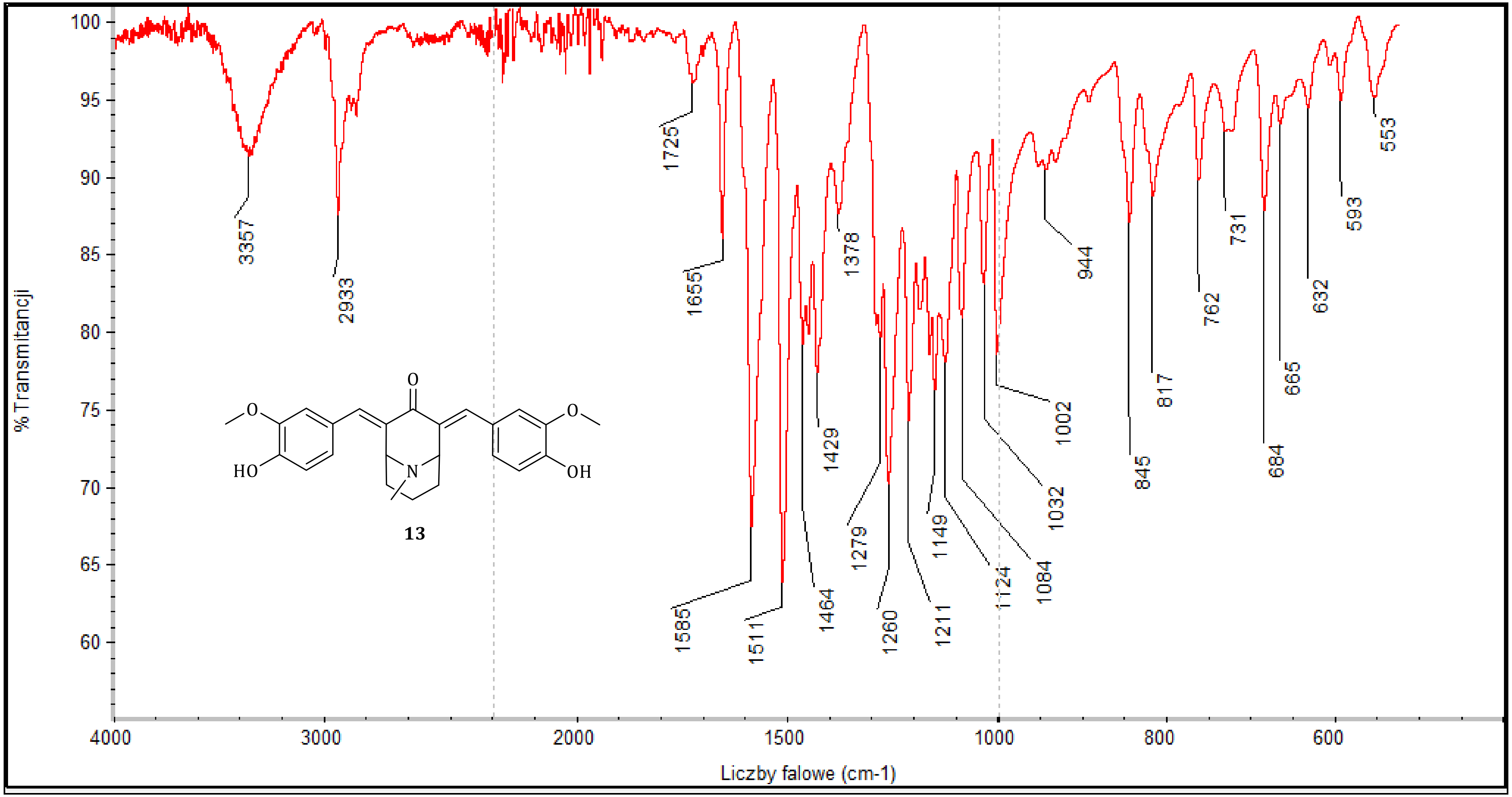

Figure S39. FTIR (ATR): 2,4-Bis((*E*)-4-hydroxy-3-methoxybenzylidene)-9-methyl-9-azabicyclo[3.3.1]nonan-3-one (**13**)

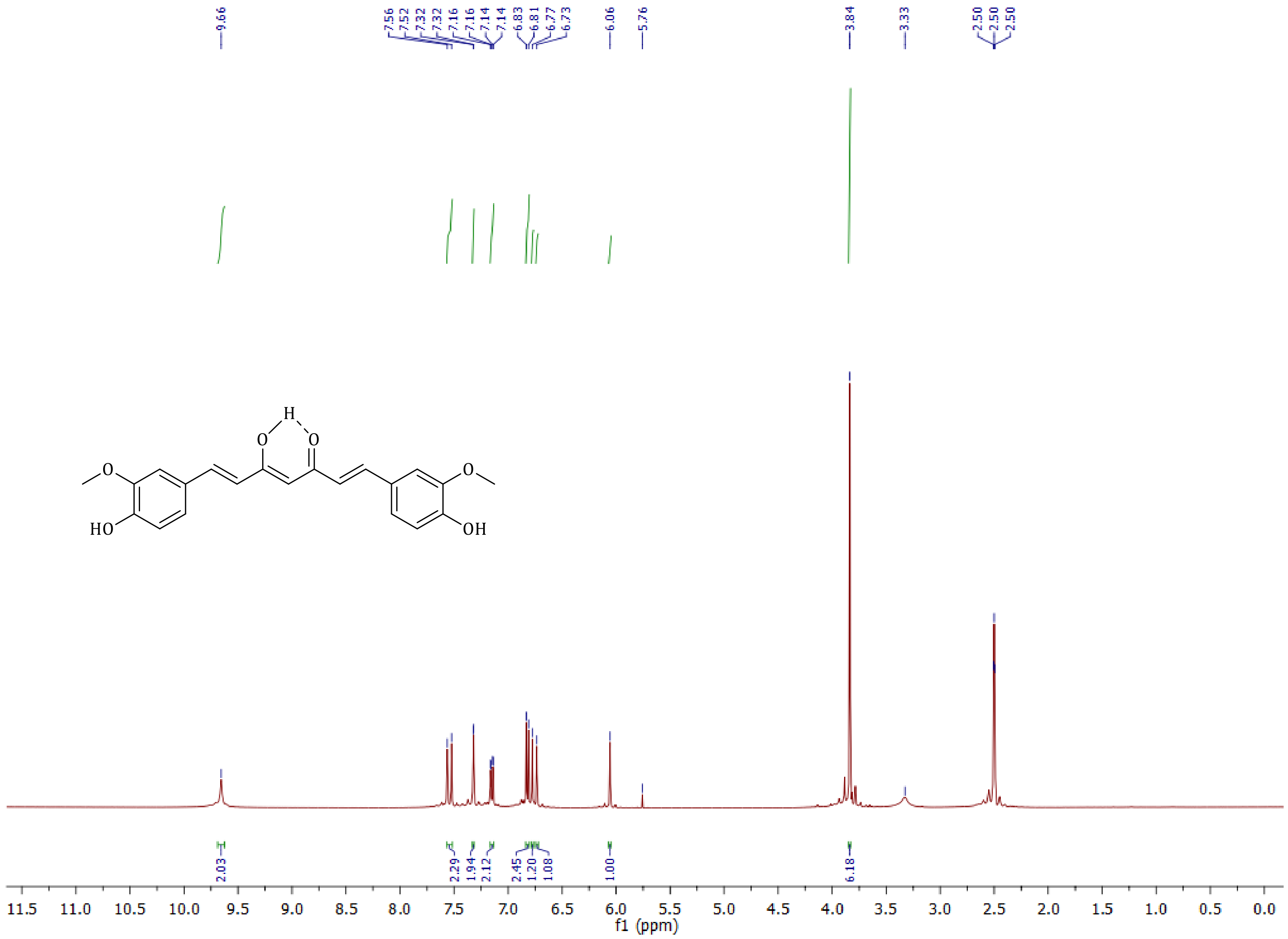

Figure S40. ^{1}H NMR (200 MHz, DMSO): CUR.

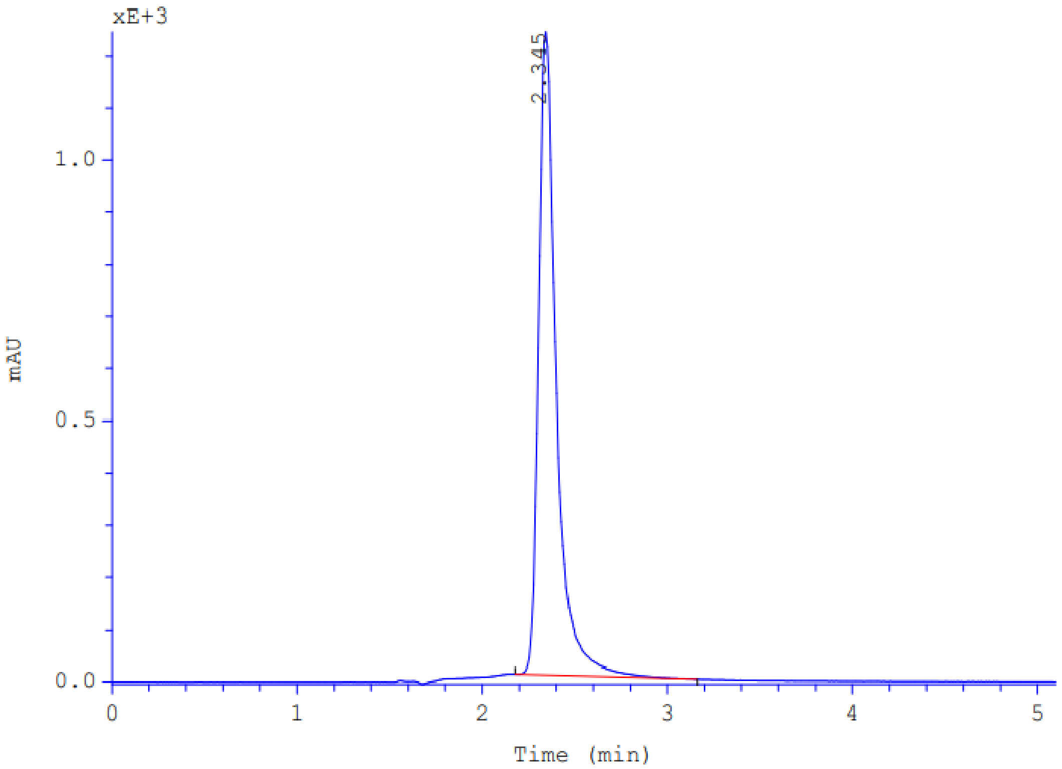

	Ret.time [min]	Start [min]	End [min]	Compound	Amount	Units	Area [mAU*min]	Height [mAU]	Width [min]	Type
1	2.345	2.18	3.16		0		138.47	1232.59	0.095	BB

Figure S41. Analysis of the purity of CUR by HPLC. Parameters: Column: Zorbax Eclipse Plus C18, 4.6 x 150 mm, 5 µm; Eluent: MeOH/H_2O (85/15; v/v), Flow rate: 1 mL/min, 427 nm, Injection volume: 10 µL; Detector: Knauer DAD K-2800; Pump: Knauer Maxi-Star K-1000; Program: EuroChrom, Version 3.05 P5.

Table S1. Crystal data and structure refinement details for compounds: **3**, **4**, **5**, **6**, **7**, **8**, **10** and **11**.

Dataset	3	4	5	6	7	8	10	11
Formula	$C_{23}H_{21}F_2NO$	$C_{23}H_{21}Cl_2NO$	$2(C_{23}H_{21}Br_2NO)$	$C_{25}H_{21}F_6NO$	$C_{23}H_{21}N_3O_5$	$C_{25}H_{27}NO_3$	$C_{27}H_{31}NO_5$	$C_{29}H_{35}NO_7$
Molecular weight	365.41	398.31	974.45	465.43	419.43	389.48	449.53	509.58
Temperature [K]	100(2)	100(2)	100(2)	100(2)	100(2)	100(2)	100(2)	100(2)
Wavelength[Å]	1.54184	1.54184	1.54184	1.54184	1.54184	1.54184	1.54184	1.54184
Crystal system	monoclinic	triclinic	triclinic	monoclinic	triclinic	monoclinic	monoclinic	triclinic
Space group	*C*2/c	*P*-1	*P*-1	$P2_1/n$	*P*-1	$P2_1/c$	$P2_1/c$	*P*-1
Unit cell parameters [Å, °]								
a	33.1187 (6)	6.9779 (2)	11.1114 (2)	16.3392 (2)	7.1824 (3)	15.9091 (2)	25.7000 (3)	10.1179 (5)
b	8.3246 (2)	7.0132 (2)	12.2568 (2)	6.9282 (1)	8.0268 (3)	18.1459 (3)	6.7437 (1)	10.8629 (6)
c	13.4451 (2)	21.6817 (5)	17.2501 (2)	18.8687 (2)	19.3231 (9)	6.9504 (1)	13.7240 (2)	11.9565 (9)
α	90	83.390 (2)	97.579 (1)	90	97.229 (4)	90	90	87.177 (5)
β	95.679 (2)	85.489 (2)	105.099 (1)	98.624 (1)	93.509 (4)	92.436 (1)	102.310 (1)	80.806 (5)
γ	90	63.120 (2)	115.166 (2)	90	116.419 (4)	90	90	78.120 (4)
Volume [Å^3]	3688.61 (11)	939.69 (5)	1972.14 (6)	2111.82 (4)	981.09 (8)	2004.66 (5)	2323.88 (5)	1269.29 (13)
Z	8	2	2	4	2	4	4	2
Absorption coefficient [mm^{-1}]	0.77	3.20	5.31	1.09	0.84	0.67	0.71	0.78
F(000)	1536	416	976	960	440	832	960	544
Crystal size [mm^3]	0.18×0.16×0.06	0.11×0.11×0.03	0.16×0.11×0.06	0.54×0.38×0.07	0.11×0.02×0.02	0.58×0.30×0.04	0.52×0.36×0.05	0.16×0.14×0.02
θ range for data collection [°]	2.7 to 78.5	2.1 to 78.1	2.8 to 78.5	3.3 to 78.2	2.3 to 75.8	2.8 to 75.8	3.5 to 78.5	3.8 to 75.9
Index ranges	−42<=h<=42<br>−10<=k<=1<br>−8<=l<=16	−8<=h<=7<br>−8<=k<=8<br>−27<=l<=27	−14<=h<=14<br>−15<=k<=15<br>−21<=l<=21	−20<=h<=20<br>−8<=k<=8<br>−23<=l<=23	−8<=h<=8<br>−10<=k<=10<br>−7<=l<=23	−16<=h<=17<br>−18<=k<=18<br>−20<=l<=20	−16<=h<=17<br>−18<=k<=18<br>−20<=l<=20	−9<=h<=12<br>−13<=k<=13<br>−15<=l<=14
Reflections collected	20228	19911	31519	23095	11176	19392	24279	14723
Independent reflections, R_{int}	3923, 0.025	3970, 0.076	8277, 0.062	4492, 0.058	3893, 0.057*	4062, 0.065	4831, 0.041	5076, 0.043
Data / restraints / parameters	3923 / 0 / 246	3970 / 0/ 245	8277 / 0 / 490	4492 / 36 / 356	3893 / 0/ 282*	4062 / 0 /266	4831 / 0 /304	5076 / 0 / 342
Goodness-of-fit on F^2	1.06	1.04	1.07	1.05	1.25*	1.04	1.04	1.06
Final *R* indices (all data)	R1 = 0.037<br>wR2 = 0.099	R1 = 0.050<br>wR2 = 0.139	R1 = 0.054<br>wR2 = 0.149	R1 = 0.045<br>wR2 = 0.120	R1 = 0.085*<br>wR2 = 0.254*	R1 = 0.048<br>wR2 = 0.130	R1 = 0.041<br>wR2 = 0.110	R1 = 0.052<br>wR2 = 0.143
Largest diff. peak and hole [e·Å^{-3}]	0.27 and -0.20	0.65 and -0.49	1.43 and -1.03	0.47 and -0.34	0.40 and -0.38*	0.39 and -0.32	0.39 and -0.22	0.74 and -0.34
CCDC number	2096435	2097003	2097005	2096436	2097179	2097004	2097006	2096434

* Values orrespond to an analysis and refinement based on crystallographic data in HKLF 5 format generated by *TwinRotMat* program.

Table S2. Solubility (S) in distilled water at 21 ± 1°C

**Compounds**	**S (g/L)**	**S (mmol/L)**
**3**	0.026	0.070
**4**	0.024	0.060
**5**	0.032	0.065
**6**	0.048	0.103
**7**	0.012	0.029
**8**	0.293	0.751
**9**	0.034	0.088
**10**	0.095	0.212
**11**	0,108	0.211
**12**	1.500	2.661
**12A** (12 x 3 HCl-salt)	448.0	665.9
**13**	1.901	4.508
